# Cereblon versus VHL: Hijacking E3 ligases against each other using PROTACs

**DOI:** 10.1016/j.bmc.2019.02.048

**Published:** 2019-06-15

**Authors:** Miriam Girardini, Chiara Maniaci, Scott J. Hughes, Andrea Testa, Alessio Ciulli

**Affiliations:** a Division of Biological Chemistry and Drug Discovery, School of Life Sciences, University of Dundee, Dow Street, Dundee DD1 5EH, Scotland, United Kingdom

**Keywords:** PROTACs, E3 ubiquitin ligases, Targeted protein degradation, von Hippel-Lindau protein, Cereblon

## Abstract

The von Hippel-Lindau (VHL) and cereblon (CRBN) proteins are substrate recognition subunits of two ubiquitously expressed and biologically important Cullin RING E3 ubiquitin ligase complexes. VHL and CRBN are also the two most popular E3 ligases being recruited by bifunctional Proteolysis-targeting chimeras (PROTACs) to induce ubiquitination and subsequent proteasomal degradation of a target protein. Using homo-PROTACs, VHL and CRBN have been independently dimerized to induce their own degradation. Here we report the design, synthesis and cellular activity of VHL-CRBN hetero-dimerizing PROTACs featuring diverse conjugation patterns. We found that the most active compound 14a induced potent, rapid and profound preferential degradation of CRBN over VHL in cancer cell lines. At lower concentrations, weaker degradation of VHL was instead observed. This work demonstrates proof of concept of designing PROTACs to hijack different E3 ligases against each other, and highlights a powerful and generalizable proximity-induced strategy to achieve E3 ligase knockdown.

## Graphical abstract

**Figure:**
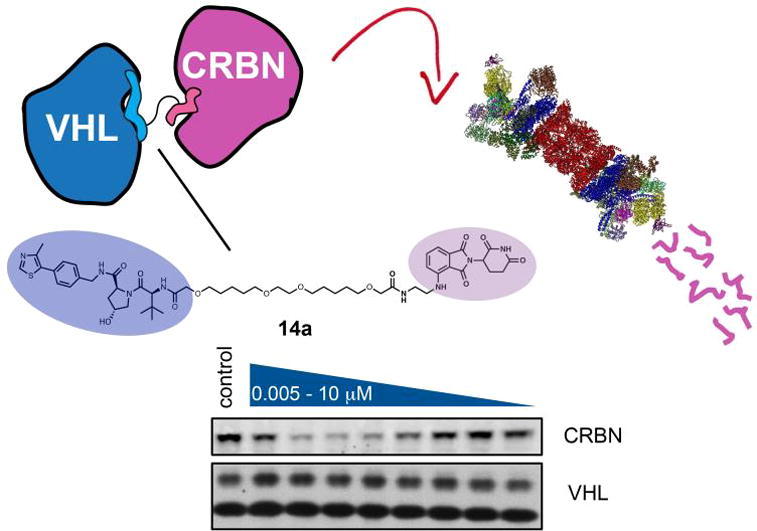

## 1 Introduction

Targeting proteins for degradation by hijacking the ubiquitin-proteasome system with small molecules is a powerful modality of intervention into biology, and an emerging therapeutic strategy.^1^, ^2^, ^3^, ^4^ A primary approach to targeted protein degradation involves the design of PROTACs (PROteolysis-Targeting Chimeras). PROTACs are bifunctional compounds that form a ternary complex with a target protein of interest and an E3 ubiquitin ligase, such that the target protein is ubiquitinated by the hijacked E3 ligase and subsequently degraded by the proteasome.^5^, ^6^ PROTACs are defined by a catalytic, sub-stoichiometric mode of action that can allow for rapid, profound and selective target depletion inside cells, and an extended duration of action, also in vivo.^7^, ^8^, ^9^, ^10^ Because their mode of action differs from that of conventional inhibitors, the concentrations at which PROTACs exert degradation activity are often much lower than expected based on their dissociation constants with the target protein.^11^, ^12^, ^13^, ^14^ Furthermore, PROTAC’s selectivity can be greater than the binding selectivity of the ligands alone, allowing to discriminate between highly similar proteins or isoforms in ways that are not possible with occupancy-based inhibitors.^8^, ^11^, ^12^, ^15^, ^16^, ^17^ Within the past four years, potent and selective PROTACs have been designed to hijack either the von Hippel-Lindau (VHL) or cereblon (CRBN) E3 ligase against a target protein of interest.^18^, ^19^ Targets that have been shown to be degraded by PROTACs include members of bromodomain-containing proteins such as the BET proteins (Brd2, Brd3 and Brd4),^7^, ^8^, ^9^, ^14^, ^15^, ^17^, ^20^, ^21^ amongst other epigenetic protein classes;^22^, ^23^, ^24^, ^25^, ^26^ protein kinases;^10^, ^12^, ^27^, ^28^, ^29^, ^30^, ^31^ as well as non-bromodomain and non-kinase target proteins.^32^, ^33^, ^34^, ^35^ Recent progress in understanding principles of PROTAC mode of action, and demonstration of applicability across different target classes, suggest that PROTACs have the potential to target new protein families, including proteins that are difficult to block using current approaches. Clinical validation of small molecules inducing protein degradation is provided by recent discoveries on the molecular mechanism of thalidomide and related clinical anticancer immunomodulatory drugs (IMiDs) such as lenalidomide and pomalidomide, which induce the proteasomal-dependent degradation of cancer-driving proteins.^36^, ^37^ More recently, a PROTAC compound (ARV-110) that targets the androgen receptor for degradation has been announced as a clinical candidate.^38^

E3 ubiquitin ligases are key players in the ubiquitin-proteasome pathway because they catalyse ubiquitination of substrate proteins.^39^, ^40^, ^41^ As important regulators of cellular ubiquitination, E3 ligases are emerging as attractive drug targets, particularly in cancer.^42^, ^43^, ^44^ However, E3 ligases have proven difficult to target using small molecule inhibitors. So far only few high-quality inhibitors have been developed, mainly against the ligases MDM2,^45^ VHL,^46^ and IAPs.^47^ E3 ligases lack deep binding sites to accommodate endogenous small-molecule cofactors or substrates, as is the case for ATP in protein kinases.^48^ Targeting E3 ligases therefore requires disruption (or modulation) of protein-protein interactions.^49^ E3 ligase inhibitors face particular challenges: first, the difficulty to compete with high-affinity endogenous substrates, which increase in level as a result of E3 blockade;^50^ and second, the observation that small molecules that bind to E3 ligases may modulate the surface of the targeted E3 in such a way that new substrate proteins are recruited for degradation, as shown for the E3 ligases CRBN,^37^, ^51^, ^52^ and DCAF15.^53^, ^54^

**Figure 1:**
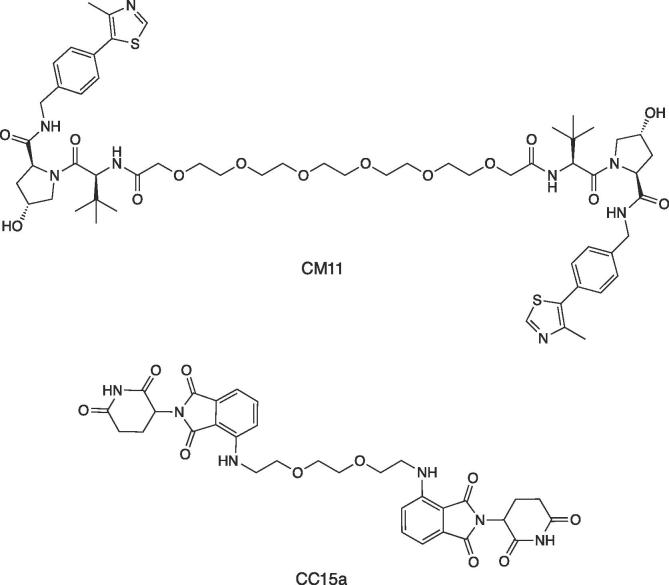
Previously published homo-PROTACs CM11 and CC15a, which induce self-degradation of the E3 ligases VHL and CRBN, respectively. CM11 and CC15a are symmetric homodimers of VHL ligand and CRBN ligand pomalidomide, respectively.

We hypothesized that the E3 ligases themselves might be hijacked against one another using a PROTAC approach, thus inducing E3 ligase degradation as opposed to E3 blockade. In 2017, we disclosed the first report of a small molecule dimerizer of an E3 ligase as a means to induce its own degradation, an approach that we called “homo-PROTAC”.^11^ We designed bifunctional molecules made up of the same ligand for the ubiquitously expressed VHL protein, connected via a linker, that would induce VHL dimerization as the key step to trigger VHL ubiquitination and subsequent degradation. The best degrader, the symmetric homo-PROTAC CM11 (Figure 1), dimerized VHL in vitro with high avidity (cooperativity) of ∼20-fold, leading to potent, complete and prolonged degradation of VHL in different cell lines. With CM11, we confirmed the hypothesized mechanism and qualified a novel chemical probe degrader for VHL.^11^ Subsequently, the same idea was applied by Krönke, Gütschow and co-workers, who reported homo-PROTACs for the CRBN ligase, and showed compound 15a (CC15a in Figure 1) to be the most active compound.^55^ As an extension of our homo-PROTAC approach, we envisaged that two different E3 ligases could be brought together using hetero-bifunctional PROTACs made of a ligand handle for one ligase and another handle for a different ligase.^56^ We hypothesized that with such compounds the two E3 ligases might be hijacked against one another, leading to two potential scenarios: 1) both ligases being degraded in cell; 2) one of the two being preferentially degraded – resulting in one ligase ‘winning’ over the other one. In the present study, we describe the design, synthesis and cellular activity of VHL-CRBN heterodimerizing PROTACs, and interrogate the outcome of hijacking these two E3 ligases against each other.

## 2 Results and discussion

### 2.1 Design of a library of CRBN-VHL PROTACs

**Figure 2:**
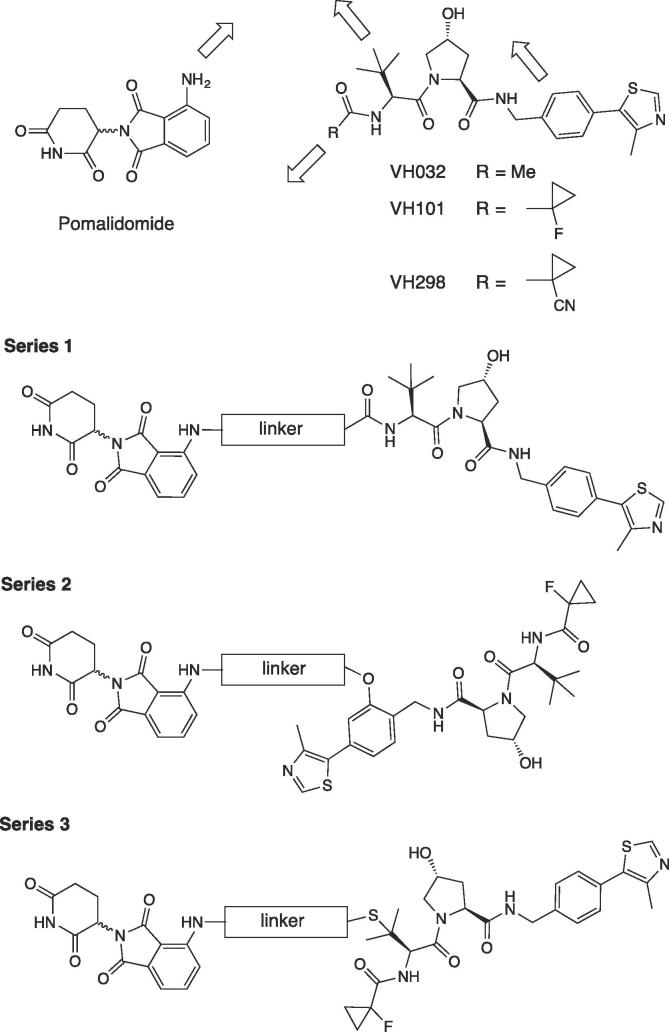
Design of VHL-CRBN conjugates explored in this work.

**Figure 3:**
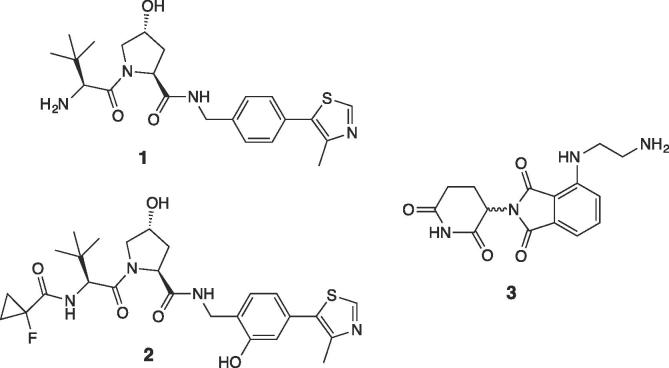
Chemical structure of VHL ligands **1** and **2**, and CRBN ligand **3**.

In order to better explore potentially different relative orientations between the two E3 ligases, we began by designing three series of heterodimerizers characterized by different attachment points on the VHL ligase handle (Figure 2): 1) out of the terminal acetyl group of VHL ligand VH032.^50^, ^57^ Amidation of a terminal *tert*-Leu of the VHL ligand (compound **1**, Figure 3) is a widely-explored conjugation strategy for PROTACs, including our homo-PROTAC CM11;^11^ 2) via a phenolic substituent out of VH101, a more potent VHL ligand in which the cyano-cyclopropyl group of chemical probe VH298 is replaced with a fluoro-cyclopropyl group, as shown in our published SAR of VHL ligands.^46^ Successful conjugation of this optimized VHL ligand (compound **2**, Figure 3) was recently reported by our laboratory in Brd9 degrader VZ185;^23^ 3) via a thioether linkage out of the *tert*-butyl group of the VHL ligand, in which the *tert*-Leu group is replaced with a penicillamine group, as we previously incorporated in Brd4-selective degrader AT1.^15^ Unlike AT1, in which the VHL ligand handle bears a terminal acetyl group, here we decided to keep the terminal fluoro-cyclopropyl group as in VH101. As CRBN handle, we chose pomalidomide because of its greater cellular stability compared to other IMiDs.^58^ To derivatize pomalidomide we appended an ethylenediamine spacer out of the phthalimide ring (compound **3**, Figure 3), to provide a synthetically convenient attachment point for amide conjugation of a linker.^23^, ^29^

The linker plays a crucial role in PROTAC design and activity. Small changes in both length and physicochemical nature e.g. alkylic versus polyethylene glycol (PEG) as well as mixtures thereof, are known to impact degradation activity and selectivity in often unpredictable ways.^21^, ^22^, ^31^ We therefore decided to explore different linkers, focusing on varying lengths and ratio between carbon and oxygen atoms, as we and others have found that these modifications can have a profound impact on PROTAC structure-activity relationships.^21^, ^22^, ^23^, ^31^ As a result, the designed compounds explore diversity in the derivatization point, linker length and chemical properties.

### 2.2 First series of PROTACs.

**Scheme 1:**
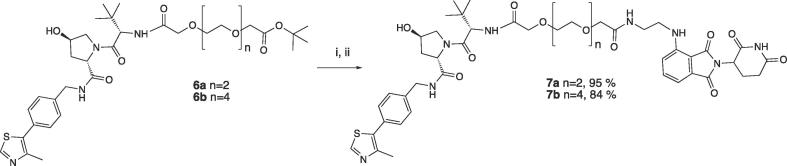
Synthesis of PROTACs **7a-b**. Conditions: i. 1:1 TFA/DCM, r.t.; ii. HATU, HOAt, **3**, DIPEA, DMF, r.t.

The first series of VHL-CRBN PROTACs (Figure 2) comprises compounds **7a,b** and **14a-e**. Compounds **6a** and **6b**, bearing respectively a 2 and 4 PEG unit linkers, were synthesized as previously reported.^11^ Briefly, triethylene or pentaethylene glycol were first converted to monobenzyl ethers and then reacted with *tert*-butyl bromoacetate under biphasic conditions to yield linkers **4a-b** in good yields (SI Scheme 1). After deprotection of the benzyl group by catalytic hydrogenation, the primary alcohol was oxidised to carboxylic acid and subsequently coupled with VHL ligand **1**, as described,^11^ to afford compounds **6a-b** (SI Scheme1). Deprotection of the *tert*-butyl group in acidic condition followed by coupling with CRBN ligand **3** afforded the final PROTACs **7a-b** in 95% and 84% yield, respectively (Scheme 1).

**Scheme 2:**
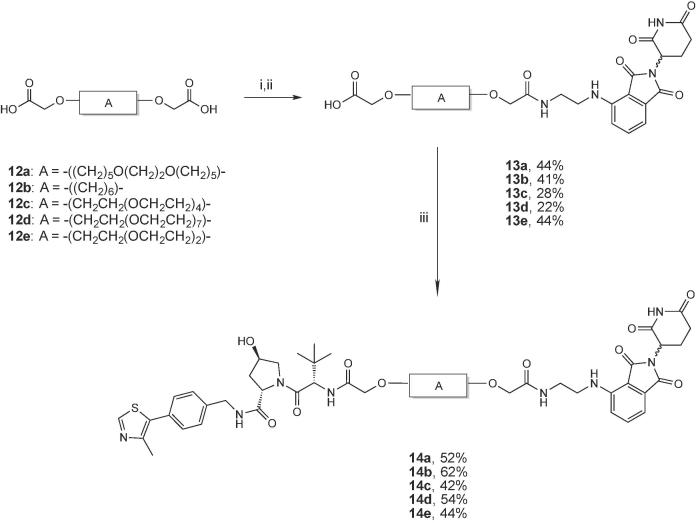
Synthesis of PROTACs **14a-e**. Reagents and conditions: i. NHS, DCC, DCM, r.t, overnight; ii. **3**, DIPEA, DMF, r.t; iii. **1**, COMU, DIPEA, DMF.

For the synthesis of PROTACs **14a-e**, symmetric linkers **12a-e** bearing two terminal carboxylate groups were designed with different length and composition. Compounds **12a-e** were prepared starting from the corresponding diols **10a-e**. Diol **10b-e** were commercially available, instead **10a** was synthesized in house by adapting a previously reported method.^31^ Briefly, **10a** was obtained after a nucleophilic substitution reaction between the tosyl derivative of a monobenzyl protected 1,5 pentadiol and ethylene glycol in a 2:1 ratio, followed by deprotection of the benzyl group by catalytic hydrogenation (SI Scheme 2). Nucleophilic substitution in phase transfer catalysis of diols **10a-e** followed by deprotection in acidic conditions, based on our previously reported synthetic route,^11^ delivered compounds **12a-e** (SI Scheme 3). Subsequently, mono *N*-hydroxysuccinamide ester derivatives of **12a-e**, obtained via reaction with *N*-hydroxysuccinimide (NHS) and *N*,*N*'-dicyclohexylcarbodiimide (DCC), were reacted with CRBN ligand **3** in a 2:1 ratio to afford **13a-e** (Scheme 2). The NHS activation of the linkers was required in order to better control the reaction and to reduce the formation of 2:1 conjugates between **3** and linkers. After removal of dicyclohexylurea (DCU) side product by filtration, the 1:1 conjugates **13a-e** were subsequently coupled with **1** to obtain the final PROTACs **14a-e** in 42–62% yields (Scheme 2).

**Scheme 3:**
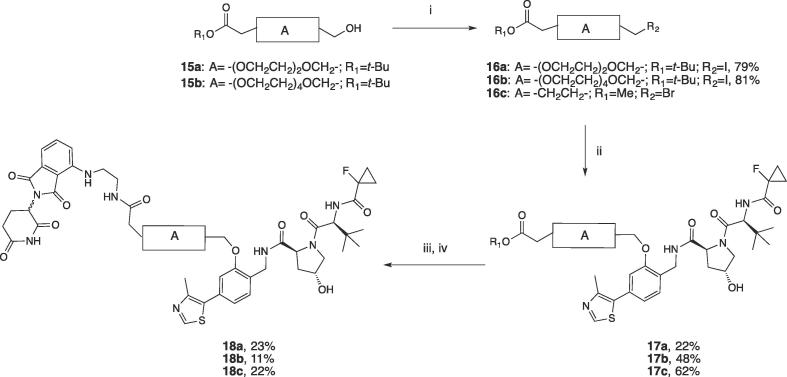
Synthesis of PROTACs **18a-c**. Reagents and conditions: i. Iodine, triphenylphosphine, imidazole, DCM, 0 °C; ii. **2**, K_2_CO_3_, DMF, 70 °C, overnight, iii. for *tert*-butyl deprotection: 1:1 TFA/DCM; for the methyl deprotection: LiOH in water/THF, 2 h, r.t; iv. COMU, **3**, DIPEA, DMF, r.t.

### 2.3 Second series of PROTACs.

Linkers for the second PROTAC series (Figure 2) were designed to contain a carboxylic group protected as *tert*-butyl ester on one side and a leaving group on the other side, which could be coupled with the phenol group on VHL ligand **2**. Linkers **15a-b** were synthesized as previously reported,^31^ and their alkyl iodide derivatives **16a-b** were prepared by reaction of the alcohol group with Ph_3_P·I_2_ reagent prepared *in situ* (Scheme 3). Ligand **2** was reacted with compounds **16a-b** and commercially available methyl 5-bromobutanoate (**16c**) in the presence of K_2_CO_3_ to afford **17a-c**, respectively, in good yields. Final PROTACs **18a-c** were obtained upon deprotection of either the *tert*-butyl group, in case of **17a-b**, or the methyl group for **17c**, and subsequent amide coupling with CRBN ligand **3**, using (1-cyano-2-ethoxy-2-oxoethylidenaminooxy)dimethylamino-morpholino-carbenium hexafluorophosphate (COMU) as coupling reagent and *N*,*N*-diisopropylethylamine (DIPEA) as base (Scheme 3).

### 2.4 Third series of PROTACs.

**Scheme 4:**
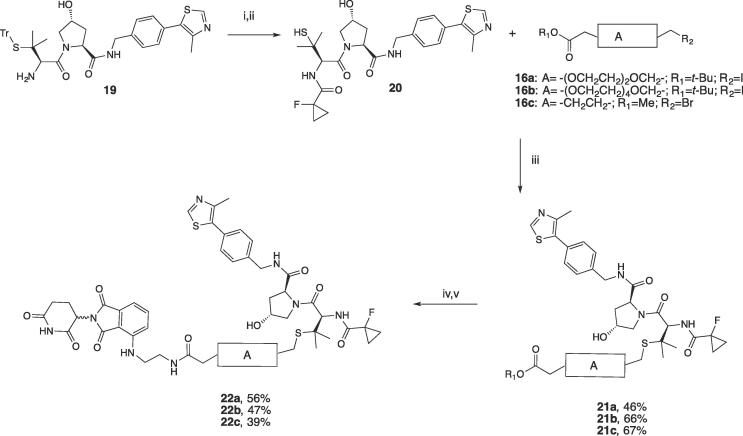
Synthesis of PROTACs **22a-c**. Reagents and conditions: i. 1-fluorocyclopropane-1-carboxylic acid, HATU, HOAt, DIPEA, DMF, r.t., 30 min; ii. TIPS, TFA, DCM, r.t., 2 h; iii. DBU, DMF, 0 °C to r.t, 4 h; iv. For *tert*-butyl deprotection: 1:1 TFA/DCM; for the methyl deprotection: LiOH in water/THF 2 h, r.t; v. COMU, **3**, DIPEA in DMF, r.t.

For the synthesis of this series of PROTACs, VHL ligand **20** was synthesized in two steps: a first coupling reaction of previously reported compound **19** (ref. ^15^) with 1-fluorocyclopropane-1-carboxylic acid, followed by deprotection of the thiol moiety (Scheme 4). Linkers **16a-c** were connected to **20** via a sulphur alkylation reaction in the presence of DBU as the base. Deprotection of the *tert*-butyl ester group of **21a-c**, and subsequent coupling with **3** under the same conditions described above, delivered the final compounds **22a-c** in good yields (Scheme 4).

### 2.5 Evaluation of PROTAC cellular activity.

**Figure 4:**
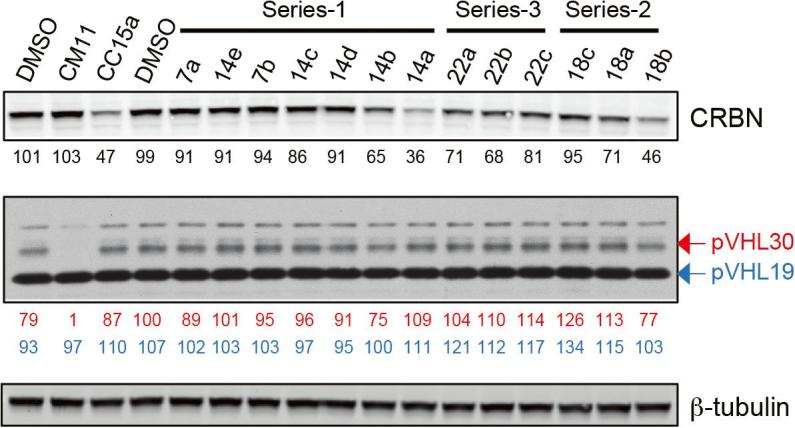
Screening of VHL-CRBN hetero-PROTACs. Western blot analysis of CRBN and VHL levels following 4 h treatment of HeLa cells with 1 µM compound. Values reported below each lane indicate protein abundance relative to the average 0.1% DMSO vehicle, and normalized for loading control.

To profile the degradation activity of our panel of PROTACs, VHL and CBRN protein levels in HeLa cells were quantified by western blot analysis following a 4 h treatment with 1 µM compounds, using CM11 and CC15a as positive controls for VHL and CRBN degradation, respectively (Figure 4). Interestingly, we observed significant degradation of CRBN with a few compounds, while no significant degradation of VHL was observed with any of the compounds tested. The most profound CRBN degradation was observed with PROTAC **14a** (64% protein degradation, as quantified by western blot), followed by compound **18b** which induced CRBN degradation to a lower extent (54% degradation). The same screen was conducted in a different cell line (HEK293), confirming **14a** as the most potent compound at inducing CRBN degradation (data not shown). To provide a more stringent screen the same experiment was conducted by testing compounds at 10 nM in HeLa cells (Figure S1). The purpose of this experiment was to exclude the possibility of dismissing any potent compound as a false negative potentially due to the “hook effect” characteristic of bivalent molecules: whereby unproductive binary complexes preferentially form at high PROTAC concentration, which compete with and eventually suppress the formation of a productive ternary complex.^9^ PROTAC **14a** induced less CRBN degradation (19% protein degradation) at 10 nM compared to 1 µM, as expected. Importantly, CRBN protein levels remaining after treatment at 10 nM were not significantly lower than the levels remaining after the same treatment at 1 µM (cf. Figure S1 with Figure 4), making it unlikely that any of the compounds might be false negatives due to a hook effect. Interestingly, at this lower concentration some compounds appeared to induce up to 50% degradation of pVHL30 (Figure S1). This suggests that depending on the concentration being used this class of compounds could preferentially induced the depletion of one ligase over the other.

**Figure 5:**
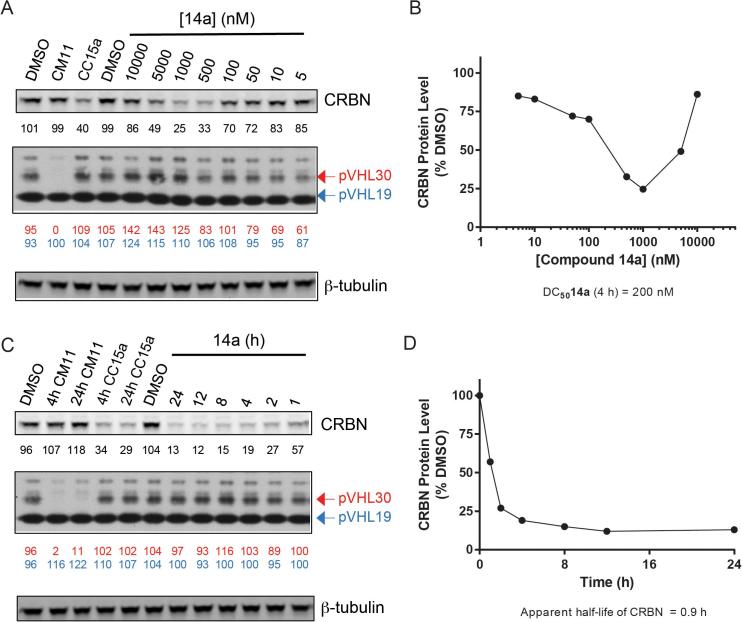
Compound 14a induces rapid depletion of CRBN, but not of VHL. (A) Western blot analysis of CRBN and VHL levels following 4 h treatment of HeLa cells with the indicated concentrations of **14a**. (B) Quantification of CRBN levels following concentration-dependent assessment. (C) Western blot analysis of CRBN and VHL levels following treatment of HeLa cells with 1 µM **14a** for the indicated time points. (D) Quantification of CRBN levels following time-dependent assessment. Values reported below each lane indicate protein abundance relative to the average 0.1% DMSO vehicle, and normalized for loading control. DC_50_ and half-lives were determined as described in the Experimental Section.

Encouraged by the promising and consistent degradation of CRBN observed with PROTAC **14a**, we selected this compound for further characterization. We next profiled the concentration- and time-dependent activity of **14a** in both HeLa (Figure 5) and HEK293 cells (Figure S2). Compound **14a** degraded CRBN with a half-degrading concentration DC_50_ (i.e. the concentration causing 50% reduction of protein level relative to vehicle) of 200 nM, and reached a maximal degradation (D_max_) of 75% after 4 h treatment with 1 µM. A hook effect was observed above 1 µM, indicating that **14a** preferentially forms the 1:1 species (i.e. acts as inhibitor) at higher concentrations. Very similar degradation profile and comparable DC_50_ and D_max_ were found for **14a** in HEK293 (Figure S2). Again, some concentration-dependent depletion of pVHL30 was seen at the lower end of the concentration range (5–50 nM) in HeLa (Figure 5). However interestingly this effect was not observed in HEK293 (Figure S2). From the time-course data, compound **14a** was able to induce rapid degradation, with >50% CRBN levels relative to control depleted already after 1 h; maximal degradation > 80% was attained after 8 h (Figure 5). The **14a**-induced degradation of CRBN was found to be even faster in HEK293, with >80% protein already depleted after 1 h, and 98% degradation achieved after 8 h (Figure S2). Once again the compound displayed selectivity for CRBN, as there was no appreciable VHL degradation at 1 µM over the time points tested in either cell line (Figure 5 and Figure S2).

## 3 Discussion

We described dually targeting CRBN-VHL PROTACs, developed with the aim of investigating the relative ability of CRBN and VHL E3 ligase to induce degradation of one other. Among the three series of compounds developed, we observed preferential degradation of one ligase i.e. CRBN over the other one (VHL) with some of the compounds from two of the series. The most potent PROTAC, compound **14a**, induced CRBN degradation with high potency (DC_50_ of 200 nM) and to profound levels (D_max_ of up to 98%) and rapidly (within 1 h of treatment). Further structure-activity relationships could help to better understand and improve the already high potency and efficiency of CRBN degradation achieved with **14a**.

Our data thus suggests that VHL can ‘win the battle’ with CRBN when the two ligases are brought together by a PROTAC. Future mechanistic studies are warranted to attempt to elucidate the contributors for this preferential unilateral outcome of our ‘double-hijacking’ approach. We also cannot exclude that different combinations of conjugation patterns (via different attachment points for example) and linker lengths and structures of CRBN-VHL PROTACs might be able to discriminate different relative orientation of the ternary complex in such a way that the outcome might become reverse, i.e. VHL being preferentially degraded over CRBN – a hypothesis that will be tested in future work. In this regard, it is interesting to note that minor concentration-dependent depletion of pVHL30 was observed at the lower end of the concentration range (5–50 nM) in HeLa (Figure 5) as well as in the screen at lower compound concentration (10 nM, Figure S2). pVHL30 is the VHL isoform that is preferentially degraded by the homo-PROTAC CM11.^11^ No observable PROTAC-induced degradation of pVHL19 was instead observed with any of our compounds, consistent with the cellular outcome observed with CM11. These observations together suggest an enticing possibility that differential ligase degradation might apply at distinct ranges of concentration of CRBN-VHL dimerizers. Differential absolute concentration between the two E3 ligases, and/or differential binding affinities of each end of the bivalent molecule for its respective ligase, are likely to be amongst the contributing factors that could effectively skew the hook effect towards one ligase versus the other one depending on the PROTAC concentration, ultimately imparting differential protein degradation outcomes. Such an effect could be of relevance in a broader context for other E3 ligase pairs. It is noteworthy that a recent study reported MDM2 PROTAC degraders, designed by linking an MDM2 inhibitor via either a thalidomide-based CRBN ligand or a VHL ligand.^59^ Potent and selective PROTAC-induced degradation of MDM2 was observed for the CRBN-MDM2 heterodimers. However notably, protein level of the hijacked CRBN or VHL ligases were not monitored.^59^ Hetero-bifunctional VHL-CRBN PROTACs were also disclosed in a study recently published by Steinebach et al.^60^ Preferential degradation of CRBN over VHL was also observed by Steinebach et al., with their most potent compound (CRBN-6-5-5-VHL) being a conjugate of pomalidomide and VHL ligand via the terminal acetyl group, as with **14a**, albeit with a different linker structure.^60^

Our study provides proof of principle for dimerizing two different E3 ligases as a novel approach to inducing one ligase to degrade the other one. The outcome of ‘ligase versus ligase’ PROTAC-mediated activity might be unpredictable *a priori*, but could reveal a new mechanism for proximity-mediated hijacking between E3 ligases. Future work is warranted to interrogate many more combinations of E3 ligases and hetero-dimerizer compounds to bring E3 ligases together as a mechanism to induce their intracellular degradation. Given the number of E3 ligases predicted to function in cells (up to 600) this approach could speed up our ability to chemically intervene on E3 ligase themselves using targeted protein degradation, with both biological and therapeutic benefits.

## 4 Experimental Section

### 4.1 Chemistry

Commercially available chemicals were purchased from Apollo Scientific, Sigma-Aldrich, Fluorochem, or Manchester Organics and used without any further purification. Compounds **1**,^8^ **2**,^23^ **3**,^23^ **6b**,^11^ **12e**,^11^ and **19**,^15^ were prepared as previously described.

All reactions were carried out using anhydrous solvents. Analytical thin-layer chromatography (TLC) was performed on precoated TLC plates (layer 0.20 mm silica gel 60 with fluorescent indicator (UV 254: Merck)). The TLC plates were air-dried and revealed under UV lamp (254/365 nm) or permanganate stain. Flash column chromatography was performed using prepacked silica gel cartridges (230–400 mesh, 40–63 mm; SiliCycle) using a Teledyne ISCO Combiflash Companion or Combiflash Retrieve using the solvent mixtures stated for each synthesis as mobile phase. Preparative HPLC was performed on a Gilson preparative HPLC with a Waters X-Bridge C18 column (100 mm × 19 mm; 5 μm particle size, flow rate 25 mL/min). Liquid chromatography–mass spectrometry (LC–MS) analyses were performed with either an Agilent HPLC 1100 series connected to a Bruker Daltonics MicroTOF or an Agilent Technologies 1200 series HPLC connected to an Agilent Technologies 6130 quadrupole spectrometer. For LC-MS the analytical cololum used was a Waters X-bridge C18 column (50 mm × 2.1 mm × 3.5 mm particle size); flow rate, 0.5 mL/min with a mobile phase of water/MeCN + 0.01% NH_4_OH (basic analytical method) or water/MeCN + 0.01% HCOOH (acidic analytical method); 95/5 water/MeCN was initially held for 0.5 min followed by a linear gradient from 95/5 to 5/95 water/MeCN over 3.5 min which was then held for 2 min. The purity of all the compounds was evaluated using the analytical LC–MS system described before, and purity was >95%. ^1^H NMR and ^13^C NMR spectra were recorded on a Bruker Avance II 500 spectrometer (^1^H at 500.1 MHz, ^13^C at 125.8 MHz) or on a Bruker DPX-400 spectrometer (^1^H at 400.1 MHz, ^13^C at 101 MHz). Chemical shifts (δ) are expressed in ppm reported using residual solvent as the internal reference in all cases. Signal splitting patterns are described as singlet (s), doublet (d), triplet (t), multiplet (m), or a combination thereof. Coupling constants (*J*) are quoted to the nearest 0.1 Hz.

#### 4.1.1 General method to obtain di-*tert*-butyl protected carboxylate (A):

To a solution of diol (1 eq.) in dichloromethane (DCM) (4 mL per mmol), *tert*-butyl bromoacetate (8 eq.), TBABr (1.1 eq.) and 37% w/w aqueous NaOH (4 mL per mmol) were added. The biphasic reaction was vigorously stirred at room temperature (r.t.) overnight. The organic phase was separated from the aqueous layer and then the aqueous phase was extracted with DCM (x3). Organic layers were collected, dried over MgSO_4_ and evaporated under reduced pressure. The crude was purified by flash chromatography (using a gradient from 10 to 100% of ethyl acetate in heptane).

#### 4.1.2 General method B:

A solution of the starting material in a 50% v/v trifluoroacetic acid (TFA) in DCM (6 mL per mmol) was stirred at r.t. for 2 h. TLC analysis (10% methanol in DCM) showed complete conversion of the starting material. Then, the reaction mixture was concentrated under reduced pressure and the crude was freeze-dried to obtain the desired product.

#### 4.1.3 General method C:

Potassium *tert*-butoxide (1 eq.) was added to polyethylene glycol (8 eq.) in anhydrous THF (0.2 mL/mmol) at 0 °C. The resulting mixture was stirred at 60 °C for 0.5 h, then it was cooled to r.t. A solution of *tert*-butyl-bromoacetate (1.0 eq.) in anhydrous THF (0.1 mL/mmol) was added to the reaction mixture at r.t. The resulting mixture was stirred at r.t. for 24 h. The reaction was quenched with brine and the aqueous phase was extracted with ethyl acetate. The combined organic phase was evaporated to dryness. The crude material was purified by column chromatography (from 0 to 8% of methanol in DCM) to afford the desired compound.

#### 4.1.4 General method D:

Iodine (1.3 eq.) was added to triphenylphosiphine (1.3 eq.) and imidazole (1.3 eq.) in DCM (7 mL/mmol) at 0 °C. The resulting mixture was stirred at r.t. for 5 min, then was cooled to 0 °C. A solution of alcohol (1.0 eq.) in DCM (3 mL/mmol) was added to the reaction mixture at 0 °C and the resulting mixture was stirred at r.t. for 3 h. TLC analysis (50% ethyl acetate in heptane) showed complete conversion of the starting material. The reaction was quenched with saturated NaHCO_3_ solution and saturated Na_2_SO_3_ solution and the aqueous phase was extracted with ethyl acetate. The combined organic phase was evaporated to dryness. The crude material was purified by column chromatography (from 20 to 75% of ethyl acetate in heptane) to afford the desired compound.

#### 4.1.5 General method E:

The dicarboxylic acid linker (1 eq.) and NHS (1.1 eq.) were dissolved in dry DCM (∼10 mL per mmol). DCC (1.2 eq.) was added and the reaction was left to stir overnight. The DCU was filtered off, the solution was evaporated and the residue dissolved in dry DMF. Compound **3** (0.5 eq.) and DIPEA (3 eq.) were added. The reaction mixture was left to stir at r.t. for 2 h, quenched with ice, dried under high vacuum and purified by HPLC using a gradient from 10% to 80% v/v acetonitrile with 0.01% v/v aqueous solution of formic acid over 15 min to yield the desired compound.

#### 4.1.6 General method F:

To a solution of carboxylic compound (1 eq.) in dry DMF (∼50 mL per mmol), COMU (1 eq.), compound **1** (1.1 eq.) and DIPEA (3 eq.) were added. The reaction mixture was left to stir for 1 h and monitored by LC-MS (acidic method). When completed, ice was added to quench the reaction, the volatiles were evaporated under reduced pressure and the residue purified by HPLC with a gradient from 5% to 90% v/v acetonitrile with 0.01% v/v aqueous solution of formic acid over 15 min to yield the desired compound.

#### 4.1.7 General method G:

To a solution of **20** (1 eq.) and the linker (1.1 eq.) in dry DMF (∼14 mL per mmol), DBU (1.1 eq.) was added at 0 °C under a nitrogen atmosphere. The reaction mixture was stirred at r.t. for 4 h and monitored by LC-MS (acidic method). The reaction was quenched with a 5% v/v aqueous solution of citric acid and the solvent was evaporated under high vacuum. The crude was purified by HPLC using a gradient from 5% to 90% v/v acetonitrile with 0.01% v/v aqueous solution of formic acid over 15 min to yield the desired compound.

#### 4.1.8 General method H:

To a solution of the carboxylic compound (1 eq.) in dry DMF (∼100 mL per mmol), COMU (1 eq.), compound **3** (1.1 eq.) and DIPEA (3 eq.) were added. The reaction mixture was left to stir for 1 h and monitored by LC-MS (acidic method). Then, ice was added to quench the reaction, the volatiles were evaporated under reduced pressure and the residue purified by HPLC using a gradient from 5% to 90% v/v acetonitrile with 0.01% v/v aqueous solution of formic acid over 15 min to yield the desired compound.

#### 4.1.9 General method I:

Compound **2** (1 eq.), K_2_CO_3_ (3 eq.) and the halogenated linker (1.5 eq.) was dissolved in DMF (∼50 mL per mmol) and heated at 70 °C overnight. Complete conversion of the starting material was observed by LC-MS (acidic method). The reaction mixture was taken up with water and extracted with DCM (x3). Organic layers were collected, dried over MgSO_4_, evaporated under reduced pressure and purified by HPLC using a gradient from 5% to 95% acetonitrile with 0.01% v/v aqueous solution of formic acid over 10 min to yield the desired compound.

#### 4.1.10 *Tert*-butyl(*S*)-16-((2*S*,4*R*)-4-hydroxy-2-((4-(4-methylthiazol-5-yl)benzyl)carbamoyl)pyrrolidine-1-carbonyl)-17,17-dimethyl-14-oxo-3,6,9,12-tetraoxa-15-azaoctadecanoate (6a)

To a solution of compound **5a** (59.83 mg, 0.22 mmol, 1 eq.), in 1.5 mL DMF, HATU (81.74 mg, 0.22 mmol, 1 eq.), HOAT (29.26 mg, 0.22 mmol, 1 eq.) were added and the solution was stirred at r.t. for 5 min. Compound **1** (100 mg, 0.215 mmol, 1 eq.) was added and the pH of the reaction mixture was adjusted to > 9 by addition of DIPEA (∼3 eq.). The mixture was stirred at r.t. until no presence of the starting materials was detected by LC-MS (acidic method). Water was added and the mixture was extracted with ethyl acetate (×3). The combined organic phases were washed with brine (×2), dried over MgSO_4_ and evaporated under reduced pressure to give the corresponding crude which was purified by HPLC using a gradient of 20% to 95% v/v acetonitrile in 0.01% aqueous solution of ammonia over 10 min to yield the final compound (72.8 mg, yield: 51%). ^1^H NMR (400 MHz, CDCl_3_) *δ* 8.94 (s, 1H), 7.42–7.36 (m, 1H), 7.32–7.20 (m, 5H), 4.67–4.63 (m, 1H), 4.53–4.42 (m, 3H), 4.31–4.25 (m, 1H), 4.01–3.87 (m, 5H), 3.64–3.55 (m, 18H), 2.47–2.34 (m, 4H), 2.11–2.04 (m, 1H), 1.40 (s, 9H), 0.91 (s, 9H); ^13^C NMR (126 MHz, CDCl_3_) *δ* 171.3, 171.1, 170.5, 170.0, 151.7, 139.1, 129.4, 128.3, 82.0, 71.1, 70.6, 70.4, 70.3, 70.2, 68.9, 58.7, 57.3, 56.8, 43.1, 36.3, 35.1, 28.1, 26.4, 15.1. MS: calculated for: C_34_H_51_N_4_O_9_S_2_ [M + H]^+^: *m*/*z* = 691.3; observed: *m*/*z* = 691.4.

#### 4.1.11 (2*S*,4*R*)-1-((2*S*)-2-(*tert*-butyl)-17-((2-(2,6-dioxopiperidin-3-yl)-1,3-dioxoisoindolin-4-yl)amino)-4,14-dioxo-6,9,12-trioxa-3,15-diazaheptadecanoyl)-4-hydroxy-*N*-(4-(4-methylthiazol-5-yl)benzyl)pyrrolidine-2-carboxamide (7a)

Following general **method B** from compound **6a** (72.3 mg, 0.11 mmol, 1 eq.), the carboxylic acid derivative was obtained as an oil. The compound was used for the next step without further purification. Yield: 99.3 mg, 0.11 mmol (quantitative). MS (ESI) *m*/*z*: [M + H]^+^ calculated for: C_30_H_42_N_4_O_9_S: 634.27; observed: 635.3.

To a solution of the crude carboxylic acid (21.16 mg, 0.028 mmol,1 eq.) in DMF (0.5 mL) was added HATU (10.64 mg, 0.028 mmol, 1 eq.) and HOAT (3.81 mg, 0.028 mmol, 1 eq.). The resulting mixture was stirred at r.t. for 5 min. Compound **3** (10 mg, 0.028 mmol, 1 eq.) was added and the pH of the reaction mixture was adjusted to > 9 by addition of DIPEA (∼3 eq.). The mixture was stirred at r.t. until no presence of the starting materials was detected by LC-MS. The solvent was evaporated under reduced pressure to give the corresponding crude which was purified by HPLC using a gradient of 5% to 95% v/v acetonitrile with 0.01% aqueous solution of formic acid over 15 min to yield the final compound as a yellow solid. Yield: 25 mg, 0.026 mmol (95%). ^1^H NMR (500 MHz, CDCl_3_) *δ* 8.62 (s, 0.5H), 8.61 (s, 0.5H), 7.60–7.54 (m, 1H), 7.48–7.38 (m, 2H), 7.30–7.25 (m, 4H), 7.01 (d, *J =* 7.4 Hz, 1H), 6.90 (d, *J =* 8.4 Hz, 1H), 6.41–6.34 (m, 1H), 4.83–4.78 (m, 1H), 4.59–4.50 (m, 2H), 4.48–4.44 (m, 1H), 4.26–4.21 (m, 1H), 3.97–3.81 (m, 5H), 3.63–3.40 (m, 12H), 2.67 (t, *J =* 4.7 Hz, 3H), 2.44 (d, *J =* 1.8 Hz, 3H), 2.35–2.29 (m, 1H), 2.12–2.05 (m, 1H), 2.03–1.97 (m, 2H), 0.89 (s, 9H); ^13^C NMR (126 MHz, CDCl_3_) *δ*172.2, 171.4, 171.3, 171.3, 170.9, 170.8, 170.3, 170.2, 169.4, 169.2, 169.1, 167.6, 150.4, 150.4, 148.4, 148.4, 146.8, 146.8, 138.5, 138.5, 136.2, 132.5, 132.5, 131.7, 131.7, 130.7, 130.7, 129.4, 129.3, 128.1, 116.7, 111.8, 111.8, 110.3, 110.2, 71.1, 70.9, 70.8, 70.3, 70.2, 70.2, 70.1, 60.4, 59.0, 59.0, 57.0, 56.9, 56.8, 50.7, 48.9, 48.9, 43.1, 41.7, 38.8, 38.6, 36.6, 35.6, 35.5, 31.5, 26.5, 26.3, 22.7, 22.7, 16.0, 14.2. HRMS: calculated for: C_45_H_57_N_8_O_12_S[M + H]^+^: *m*/*z* = 933.3811; observed: *m*/*z* = 933.3826.

#### 4.1.12 *N*_1_-(2-((2-(2,6-dioxopiperidin-3-yl)-1,3-dioxoisoindolin-4-yl)amino)ethyl)-*N*_17_-((*S*)-1-((2*S*,4*R*)-4-hydroxy-2-((4-(4-methylthiazol-5-yl)benzyl)carbamoyl)pyrrolidin-1-yl)-3,3-dimethyl-1-oxobutan-2-yl)-3,6,9,12,15-pentaoxaheptadecanediamide (7b)

To a solution of the Boc-deprotected carboxylic acid derivative of compound **6b** (obtained as described in ref.^11^) (23.88 mg, 0.028 mmol, 1 eq.) in dry DMF (0.5 mL), HATU (10.64 mg, 0.028 mmol, 1 eq.) and HOAT (3.81 mg, 0.028 mmol, 1 eq.) were added. The solution was stirred for 5 min, compound **3** (10 mg, 0.028 mmol, 1 eq.) was added and the pH of the reaction was adjusted to >9 with DIPEA. The mixture was stirred at r.t. until no presence of the starting materials was detected by LC-MS. Water was added and the mixture was extracted with ethyl acetate (×3). The combined organic phases were washed with brine (×2), dried over MgSO_4_ and evaporated under reduced pressure to give the corresponding crude, which was purified by HPLC using a gradient of 20% to 95% v/v acetonitrile with 0.01% aqueous solution of ammonia over 10 min to yield the final compound as a white solid (24 mg, yield: 84%). ^1^H NMR (500 MHz, CDCl_3_) *δ* 8.70 (s, 0.5H), 8.69 (s, 0.5H), 7.64–7.58 (m, 1H), 7.55–7.49 (m, 1H), 7.43–7.40 (m, 1H), 7.32–7.27 (m, 5H), 7.02–6.95 (m, 2H), 4.84–4.77 (m, 1H), 4.66–4.59 (m, 1H), 4.56–4.48 (m, 2H), 4.28–4.23 (m, 1H), 3.95–3.82 (m, 4H), 3.63–3.36 (m, 22H), 2.46–2.44 (m, 4H), 2.41–2.34 (m, 1H), 2.14–2.08 (m, 1H), 0.90 (s, 9H); ^13^C NMR (126 MHz, CDCl_3_) δ: 171.3, 171.2, 171.1, 170.5, 170.3, 169.4, 168.9, 167.6, 150.7, 150.6, 147.9, 146.8, 146.8, 138.7, 138.6, 136.2, 132.5, 132.0, 130.5, 130.4, 129.4, 129.3, 128.2, 117.0, 111.7, 110.1, 70.9, 70.6, 70.5, 70.4, 70.3, 70.2, 70.1, 70.0, 59.0, 58.8, 57.0, 56.9, 48.9, 43.2, 41.9, 38.4, 38.3, 36.5, 35.4, 35.3, 31.5, 26.4, 22.7, 15.8. HRMS calculated for: C_49_H_65_N_8_O_14_S [M + H]^+^: *m*/*z* = 1021.4335; observed: *m*/*z* = 1021.4546 [M + H]^+^.

#### 4.1.13 3,9,12,18-tetraoxaicosanedioic acid (12a)

Starting from compound **11a** (83 mg, 0.17 mmol) and following the **general method B** compound **12a** was obtained in quantitative yield (64 mg). ^1^H NMR (500 MHz, CDCl_3_) δ: 8.06 (s, 2H), 4.09 (s, 4H), 3.59 (s, 4H), 3.56 (t, *J* = 6.5 Hz, 4H), 3.49 (t, *J* = 6.5 Hz, 4H), 1.66–1.58 (m, 8H), 1.47–1.41 (m, 4H); ^13^C NMR (101 MHz, CDCl_3_) δ: 174.4, 72.0, 71.4, 70.1, 67.9, 29.2, 29.1, 22.6.

#### 4.1.14 1-((2-(2,6-dioxopiperidin-3-yl)-1,3-dioxoisoindolin-4-yl)amino)-4-oxo-6,12,15,21-tetraoxa-3-azatricosan-23-oic acid (13a)

Starting from compound **12a** (36 mg, 0.098 mmol,1 eq) and following the **general method E**, the title compound was obtained (14 mg, yield: 44%). ^1^H NMR (500 MHz, CDCl_3_) δ: 7.48 (dd, *J* = 7.2, 8.6 Hz, 1H), 7.09 (d, *J* = 7.1 Hz, 1H), 6.98 (d, *J* = 8.9 Hz, 1H), 4.93–4.89 (m, 1H), 4.04 (s, 2H), 3.93 (s, 2H), 3.55–3.41 (m, 16H), 2.88–2.66 (m, 3H), 2.13–2.06 (m, 1H), 1.64–1.52 (m, 8H), 1.46–1.32 (m, 4H); ^13^C NMR (126 MHz, CDCl_3_) δ: 172.4, 171.7, 171.4, 169.5, 168.8, 167.7, 146.9, 136.4, 132.7, 116.9, 112.1, 110.7, 71.9, 71.3, 71.2, 70.3, 70.2, 68.1, 49.1, 42.3, 38.6, 31.6, 29.4, 29.3, 22.9, 22.7. MS: calculated for C_31_H_44_N_4_O_11_ [M + H]^+^: *m*/*z* = 649.3; observed: *m*/*z* = 649.0

#### 4.1.15 *N*^1^-(2-((2-(2,6-dioxopiperidin-3-yl)-1,3-dioxoisoindolin-4-yl)amino)ethyl)-*N*^20^-((*S*)-1-((2*S*,4*R*)-4-hydroxy-2-((4-(4-methylthiazol-5-yl)benzyl)carbamoyl)pyrrolidin-1-yl)-3,3-dimethyl-1-oxobutan-2-yl)-3,9,12,18-tetraoxaicosanediamide (14a)

Starting from compound **13a** (14 mg, 0.02 mmol, 1 eq.) and following the **general method F**, the title compound was obtained (12 mg, yield: 52%). ^1^H NMR (400 MHz, MeOD) δ: 9.00 (s, 1H), 7.56–7.52 (m, 1H), 7.48–7.41 (m, *J* = 8.5, 20.5 Hz, 4H), 7.14 (d, *J* = 8.5 Hz, 1H), 7.05 (d, *J* = 7.5 Hz, 1H), 5.05 (dd, *J* = 5.2, 12.7 Hz, 1H), 4.70 (d, *J* = 10.3 Hz, 1H), 4.60–4.50 (m, 3H), 4.36 (d, *J* = 16.0 Hz, 1H), 3.96 (d, *J* = 6.9 Hz, 2H), 3.91–3.78 (m, 4H), 3.47 (m, *J* = 7.9, 28.1 Hz, 16H), 2.90–2.64 (m, 3H), 2.48 (s, 3H), 2.26–2.21 (m, 1H), 2.12–2.05 (m, 2H), 1.68–1.33 (m, 12H), 1.03 (s, 9H); ^13^C NMR (101 MHz, MeOD) δ: 174.5, 174.2, 173.4, 172.0, 171.9, 171.6, 171.4, 170.5, 169.1, 153.1, 148.2, 148.0, 140.4, 137.1, 133.8, 133.7, 131.0, 130.3, 129.4, 128.9, 118.0, 112.0, 111.3, 72.7, 72.6, 72.0, 71.0, 70.8, 70.6, 66.8, 60.7, 58.0, 57.9, 43.6, 42.6, 39.3, 38.8, 37.1, 32.1, 30.3, 30.1, 26.8, 23.7, 23.6, 15.5, 15.3. HRMS: calculated for C_53_H_73_N_8_O_13_S [M + H]^+^: *m*/*z* = 1061.5012; observed: *m*/*z* = 1061.5065.

#### 4.1.16 2,2′-(hexane-1,6-diylbis(ox))diacetic acid (12b)

Starting from compound **11b** (185 mg, 0.53 mmol) and following the **general method B** compound **12b** was obtained in quantitative yield (125 mg). ^1^H NMR (400 MHz, DMSO) δ: 12.49 (s, 2H), 3.97 (s, 4H), 3.43 (t, *J* = 6.6 Hz, 4H), 1.55–1.47 (m, 4H), 1.34–1.28 (m, 4H); ^13^C-NMR (101 MHz, DMSO) *δ* 171.6, 70.4, 67.4, 29.0, 25.3.

#### 4.1.17 2-((6-(2-((2-((2-(2,6-dioxopiperidin-3-yl)-1,3-dioxoisoindolin-4-yl)amino)ethyl)amino)-2-oxoethoxy)hexyl)oxy)acetic acid (13b)

To a solution of compound **12b** (15.0 mg, 0.064 mmol, 1 eq.) and following the **general method E**, the title compound was obtained (7 mg, yield: 41%). ^1^H NMR (400 MHz, MeOD) δ: 7.56 (dd, *J* = 7.1, 8.6 Hz, 1H), 7.15 (d, *J* = 8.6 Hz, 1H), 7.07 (d, *J* = 7.1 Hz, 1H), 5.05 (dd, *J* = 5.5, 12.5 Hz, 1H), 4.02 (s, 2H), 3.90 (s, 2H), 3.52–3.45 (m, 8H), 2.91–2.67 (m, 3H), 2.15–2.08 (m, 1H), 1.63–1.55 (m, 4H), 1.38–1.34 (m, 4H); ^13^C NMR (126 MHz, MeOD) δ: 174.6, 174.1, 173.5, 171.5, 170.6, 169.3, 148.2, 137.2, 134.0, 118.1, 112.1, 111.5, 72.8, 72.6, 70.9, 68.7, 42.7, 39.4, 32.2, 30.4, 26.8, 23.8. MS: calculated for C_25_H_33_N_4_O_9_ [M + H]^+^ : *m*/*z* = 532.2 ; observed: *m*/*z* = 533.3.

#### 4.1.18 (2*S*,4*R*)-1-((2*S*)-2-(*tert*-butyl)-18-((2-(2,6-dioxopiperidin-3-yl)-1,3-dioxoisoindolin-4-yl)amino)-4,15-dioxo-6,13-dioxa-3,16-diazaoctadecanoyl)-4-hydroxy-*N*-(4-(4-methylthiazol-5-yl)benzyl)pyrrolidine-2-carboxamide (14b)

Starting from compound **13b** (7 mg, 0.013 mmol, 1 eq) and following the **general method F**, the title compound was obtained (7.7 mg, yield: 62%). ^1^H NMR (400 MHz, MeOD) δ: 8.99 (s, 1H), 7.56–7.49 (m, 1H), 7.44 (dd, *J* = 7.9, 21.3 Hz, 4H), 7.11 (d, *J* = 8.9 Hz, 1H), 7.05 (d, *J* = 7.0 Hz, 1H), 5.04 (dd, *J* = 5.6, 12.6 Hz, 1H), 4.71–4.68 (m, 1H), 4.61–4.33 (m, 4H), 3.95 (d, *J* = 6.3 Hz, 2H), 3.91–3.78 (m, 4H), 3.54–3.42 (m, 8H), 2.85–2.63 (m, 3H), 2.48 (s, 3H), 2.26–2.21 (m, 1H), 2.10–2.07 (m, 2H), 1.63–1.56 (m, 4H), 1.40–1.31 (m, 4H), 1.03 (s, 9H). ^13^C-NMR (101 MHz, MeOD) δ: 174.4, 174.1, 173.3, 171.8, 171.5, 171.3, 170.4, 169.0, 153.0, 148.0, 140.3, 137.0, 133.8, 130.9, 130.2, 128.9, 117.9, 112.0, 111.2, 72.7, 72.5, 70.9, 70.7, 70.5, 60.6, 57.9, 57.8, 43.5, 42.5, 39.2, 38.7, 37.0, 32.0, 30.3, 30.2, 26.7, 26.6, 23.6, 15.2. HRMS: calculated for C_47_H_61_N_8_O_11_S [M + H]^+^: *m*/*z* = 945.4175; observed: *m*/*z* = 945.4270.

#### 4.1.19 3,6,9,12,15,18-hexaoxaicosanedioic acid (12c)

Starting from compound **11c** (300 mg, 0.64 mmol) and following the **general method B** compound **12c** was obtained in quantitative yield (226 mg). Analytical data matched those previously reported.^61^

#### 4.1.20 1-((2-(2,6-dioxopiperidin-3-yl)-1,3-dioxoisoindolin-4-yl)amino)-4-oxo-6,9,12,15,18,21-hexaoxa-3-azatricosan-23-oic acid (13c)

Starting from compound **12c** (23 mg, 0.065 mmol, 1 eq.) and following the **general method E**, the title compound was obtained (5.9 mg, 28%). ^1^H NMR (400 MHz, CDCl_3_) δ: 7.49 (t, *J* = 7.8 Hz, 1H), 7.08 (d, *J* = 6.8 Hz, 1H), 7.03 (d, *J* = 8.4 Hz, 1H), 4.93–4.86 (m, 1H), 4.10 (s, 2H), 3.97 (s, 2H), 3.72–3.45 (m, 24H), 2.89–2.65 (m, 3H), 2.13–2.07 (m, 1H); ^13^C NMR (126 MHz, CDCl_3_) δ :172.5, 171.4, 171.3, 169.4, 168.7, 167.8, 147.0, 136.4, 132.7, 117.0, 111.9, 110.5, 71.0, 70.5, 70.4, 70.3, 70.2, 69.4, 49.1, 42.0, 38.5, 31.6, 22.9. MS: calculated for C_29_H_41_N_4_O_13_[M + H]^+^: *m*/*z* = 653.2 observed: *m*/*z* = 653.3.

#### 4.1.21 *N*^1^-(2-((2-(2,6-dioxopiperidin-3-yl)-1,3-dioxoisoindolin-4-yl)amino)ethyl)-*N*^20^-((*S*)-1-((2*S*,4*R*)-4-hydroxy-2-((4-(4-methylthiazol-5-yl)benzyl)carbamoyl)pyrrolidin-1-yl)-3,3-dimethyl-1-oxobutan-2-yl)-3,6,9,12,15,18-hexaoxaicosanediamide (14c)

Starting from compound **13c** (5.9 mg, 0.0090 mmol, 1 eq.) and following the **general method F**, the title compound was obtained (4 mg, yield: 42%). ^1^H NMR (400 MHz, MeOD) δ: 8.86 (s, 1H), 7.55 (dd, *J* = 7.1, 8.5 Hz, 1H), 7.44 (dd, *J* = 8.2, 18.7 Hz, 4H), 7.14 (d, *J* = 8.5 Hz, 1H), 7.05 (d, *J* = 7.1 Hz, 1H), 5.04 (dd, *J* = 5.6, 12.8 Hz, 1H), 4.70 (s, 1H), 4.61–4.48 (m, 3H), 4.35 (d, *J* = 14.2 Hz, 1H), 4.03 (d, *J* = 4.4 Hz, 2H), 3.95 (s, 2H), 3.90–3.77 (m, 2H), 3.71–3.58 (m, 20H), 3.52–3.49 (m, 4H), 2.89–2.65 (m, 3H), 2.47(s, 3H), 2.26–2.18 (m, 1H), 2.14–2.05 (m, 2H), 1.04 (s, 9H).^13^C-NMR (101 MHz, MeOD) *δ* 174.7, 174.4, 173.6, 172.1, 171.7, 171.5, 170.6, 169.3, 152.8, 149.1, 148.2, 140.3, 137.3, 134.0, 133.4, 131.6, 130.4, 129.0, 118.2, 112.1, 111.5, 72.3, 72.0, 71.7, 71.6, 71.5, 71.3, 71.1, 60.8, 58.2, 58.1, 43.8, 42.5, 39.4, 38.9, 37.1, 32.2, 27.0, 23.8, 15.9. HRMS: calculated for C_51_H_72_N_9_O_15_S [M + NH_4_]^+^: *m*/*z* = 1082.4863; observed: *m*/*z* = 1082.4790.

#### 4.1.22 3,6,9,12,15,18,21,24,27-nonaoxanonacosanedioic acid (12d)

Starting from compound **11d** (100 mg, 0.17 mmol) and following the **general method B** compound **12d** was obtained in quantitative yield (80 mg). ^1^H NMR (400 MHz, D_2_O) δ: 4.32 (4H, s), 3.87–3.78 (32H, m).

#### 4.1.23 1-((2-(2,6-dioxopiperidin-3-yl)-1,3-dioxoisoindolin-4-yl)amino)-4-oxo-6,9,12,15,18,21,24,27,30-nonaoxa-3-azadotriacontan-32-oic acid (13d)

Starting from compound **12d** (4.6 mg, 0.010 mmol, 1 eq.) and following the **general method E**, the title compound was obtained (8.5 mg, yield: 22%). ^1^H NMR (500 MHz, CDCl_3_) δ: 7.49 (dd, *J* = 7.2, 8.5 Hz, 1H), 7.08 (d, *J* = 7.1 Hz, 1H), 7.02 (d, *J* = 8.6 Hz, 1H), 4.89 (dd, *J* = 5.5, 12.3 Hz, 1H), 4.12 (s, 2H), 3.98 (s, 2H), 3.72–3.48 (m, 36H), 2.89–2.69 (m, 3H), 2.12–2.08 (m, 1H); ^13^C NMR (126 MHz, CDCl_3_) δ: 172.1, 171.4, 169.4, 168.6, 167.7, 147.0, 136.4, 132.7, 117.0, 111.9, 110.5, 71.1, 70.8, 70.7, 70.6, 70.5, 70.4, 70.2, 69.3, 49.1, 42.1, 38.6, 31.6, 22.9. MS: calculated for C_35_H_53_N_4_O_16_[M + H]^+^: *m*/*z* = 785.3; observed: *m*/*z* = 785.8.

#### 4.1.24 *N*^1^-(2-((2-(2,6-dioxopiperidin-3-yl)-1,3-dioxoisoindolin-4-yl)amino)ethyl)-*N*^29^-((*S*)-1-((2*S*,4*R*)-4-hydroxy-2-((4-(4-methylthiazol-5-yl)benzyl)carbamoyl)pyrrolidin-1-yl)-3,3-dimethyl-1-oxobutan-2-yl)-3,6,9,12,15,18,21,24,27-nonaoxanonacosanediamide (14d)

Starting from compound **13d** (8.5 mg, 0.0108 mmol, 1 eq.) and following the **general method F**, the title compound was obtained (7.0 mg, yield: 54%). ^1^H NMR (500 MHz, MeOD) δ: 8.87 (s, 1H), 7.55 (dd, *J* = 7.2, 8.6 Hz, 1H), 7.44 (dd, *J* = 8.6, 22.4 Hz, 4H), 7.15 (d, *J* = 8.4 Hz, 1H), 7.06 (d, *J* = 7.0 Hz, 1H), 5.04 (dd, *J* = 5.2, 12.4 Hz, 1H), 4.70 (d, *J* = 9.4 Hz, 1H), 4.60–4.50 (m, 3H), 4.36 (dd, *J* = 4.9, 15.8 Hz, 1H), 4.04 (d, *J* = 6.1 Hz, 2H), 3.96 (s, 2H), 3.89–3.79 (m, 2H), 3.71–3.51 (m, 36H), 2.88–2.66 (m, 3H), 2.47 (s, 3H), 2.24–2.20 (m, 1H), 2.12–2.07 (m, 2H), 1.04 (s, 9H); ^13^C-NMR (126 MHz, MeOD) δ: 174.6, 174.3, 173.5, 172.1, 171.7, 171.4, 170.6, 169.2, 152.8, 149.1, 148.2, 140.3, 137.3, 134.0, 133.4, 131.5, 130.5, 130.4, 129.5, 129.0, 118.2, 112.1, 111.5, 72.3, 72.0, 71.6, 71.5, 71.4, 71.3, 71.1, 71.0, 60.8, 58.1, 43.7, 42.5, 39.4, 38.9, 37.1, 32.2, 27.0, 23.8, 15.8. HRMS: calculated for C_57_H_89_N_9_O_18_S [M + NH_4_]^+^: *m*/*z* = 1214.5650; observed: *m*/*z* = 1214. 5675.

#### 4.1.25 1-((2-(2,6-dioxopiperidin-3-yl)-1,3-dioxoisoindolin-4-yl)amino)-4-oxo-6,9,12,15-tetraoxa-3-azaheptadecan-17-oic acid (13e)

Starting from compound **12e** (26.6 mg, 0.10 mmol, 1 eq.) and following the **general method E**, the title compound was obtained (12.5 mg, 44%). ^1^H NMR (400 MHz, CDCl_3_) δ: 7.47 (dd, *J* = 7.2, 8.5 Hz, 1H), 7.06 (d, *J* = 7.2 Hz, 1H), 7.01 (d, *J* = 8.5 Hz, 1H), 4.90 (dd, *J* = 5.6, 11.7 Hz, 1H), 4.08 (s, 2H), 3.99 (s, 2H), 3.72–3.47 (m, 16H), 2.87–2.70 (m, 3H), 2.13–2.05 (m, 1H); ^13^C NMR (126 MHz, CDCl_3_) δ: 172.6, 171.7, 171.4, 169.5, 169.0, 167.7, 147.0, 136.4, 132.6, 117.1, 111.9, 110.4, 71.1, 71.0, 70.5, 70.3, 69.0, 49.1, 42.1, 38.5, 31.6, 22.9. MS analysis: calculated for C_25_H_33_N_4_O_11_ [M + H]^+^: 565.2 observed: 565.3.

#### 4.1.26 *N*^1^-(2-((2-(2,6-dioxopiperidin-3-yl)-1,3-dioxoisoindolin-4-yl)amino)ethyl)-*N*^14^-((*S*)-1-((2*S*,4*R*)-4-hydroxy-2-((4-(4-methylthiazol-5-yl)benzyl)carbamoyl)pyrrolidin-1-yl)-3,3-dimethyl-1-oxobutan-2-yl)-3,6,9,12-tetraoxatetradecanediamide (14e)

Starting from compound **13e** (12.5 mg, 0.022 mmol, 1 eq.) and following the **general method F**, the title compound was obtained (9.5 mg, yield: 44%). ^1^H NMR (400 MHz, MeOD) δ: 8.87 (s, 1H), 7.54 (dd, *J* = 7.1, 8.4 Hz, 1H), 7.42 (dd, *J* = 8.3, 20.1 Hz, 4H), 7.12 (d, *J* = 8.4 Hz, 1H), 7.04 (d, *J* = 7.1 Hz, 1H), 5.03 (dd, *J* = 5.5, 12.6 Hz, 1H), 4.70 (d, *J* = 7.1 Hz, 1H), 4.61–4.48 (m, 3H), 4.35 (d, *J* = 15.3 Hz, 1H), 4.03 (d, *J* = 3.2 Hz, 2H), 3.94 (s, 2H), 3.88–3.78 (m, 2H), 3.70–3.49 (m, 16H), 2.89–2.66 (m, 3H), 2.46 (s, 3H), 2.25–2.20 (m, 1H), 2.13–2.06 (m, 2H), 1.03 (s, 9H). ^13^C NMR (101 MHz, MeOD) δ: 174.7, 174.4, 173.6, 172.0, 171.7, 171.5, 170.6, 169.3, 152.8, 149.0, 148.2, 140.3, 137.3, 134.0, 133.4, 131.5, 130.4, 129.0, 112.2, 111.5, 72.2, 72.0, 71.5, 71.4, 71.3, 71.1, 60.8, 58.1, 43.8, 42.5, 39.5, 38.9, 37.1, 32.2, 27.0, 23.8, 15.8. HRMS: calculated for C_47_H_61_N_8_O_13_S [M + H]^+^: *m*/*z* = 977.4073; observed: *m*/*z* = 977.4079.

#### 4.1.27 *Tert*-butyl-2-(2-(2-(2-hydroxyethoxy)ethoxy)ethoxy)acetate (15a)

Starting from triethylene glycol (6.9 g, 41 mmol) and following the **general method C**, the title compound was obtained (540 mg, yield: 40%). ^1^H NMR (500 MHz, MeOD) δ: 4.02 (s, 2H), 3.69–3.61 (m, 10*H*), 3.55 (t, *J* = 4.9 Hz, 2H), 1.48 (s, 9H). Analytical data matched those previously reported.^31^

#### 4.1.28 *Tert*-butyl-2-(2-(2-(2-iodoethoxy)ethoxy)ethoxy)acetate (16a)

Starting from compound **15a** (350 mg, 1.33 mmol) and following the **general method D**, the title compound was obtained. Yield: 414 mg (79%). ^1^H NMR (400 MHz, CDCl_3_) δ: 4.06 (s, 2H), 3.81–3.70 (m, 10*H*), 3.28 (t, *J* = 6.9 Hz, 2H), 1.50 (s, 9H). ^13^C NMR (101 MHz, CDCl_3_) δ: 169.7, 81.6, 72.0, 70.7, 70.6, 70.2, 69.1, 69.0, 28.1.

#### 4.1.29 *Tert*-butyl-2-(2-(2-(2-(2-(((2*S*,4*R*)-1-((*S*)-2-(1-fluorocyclopropane-1-carboxamido)-3,3-dimethylbutanoyl)-4-hydroxypyrrolidine-2-carboxamido)methyl)-5-(4-methylthiazol-5-yl)phenoxy)ethoxy)ethoxy)ethoxy)acetate (17a)

Starting from compound **2** (40 mg, 0.075 mmol, 1 eq.), **16a** (42 mg, 0.11 mmol, 1.5 eq.) and K_2_CO_3_ (31 mg, 0.22, 3 eq.), and following the **general method I**, the titled compound was obtained (13 mg, yield: 22%). ^1^H NMR (500 MHz, CDCl_3_) δ: 8.66 (s, 1H), 7.00 (dd, *J* = 3.4, 8.9 Hz, 1H), 6.95 (dd, *J* = 1.6, 7.6 Hz, 1H), 6.88 (d, *J* = 1.6 Hz, 1H), 4.65 (t, *J* = 8.0 Hz, 1H), 4.53–4.44 (m, 4H), 4.24–4.14 (m, 2H), 4.00–3.59 (m, 14H), 2.50 (s, 3H), 2.42–2.36 (m, 1H), 2.13–2.08 (m, 1H), 1.44 (s, 9H), 1.32–1.22 (m, 4H), 0.94 (s, 9H); ^13^C NMR (126 MHz, CDCl_3_) δ: 170.8, 170.7, 170.1 (d, *^2^J* = 20.4 Hz), 169.8, 157.0, 150.4, 148.7, 132.5, 131.9, 130.0, 127.1, 122.2, 113.0, 81.8, 79.5, 70.9, 70.7, 70.4, 69.8, 69.2, 68.1, 58.8, 57.6, 56.7, 39.3, 36.6, 35.7, 28.3, 26.5, 16.3, 13.8 (d, *^2^J* = 5.2 Hz), 13.7 (d, *^2^J* = 5.2 Hz). MS: calculated for C_38_H_56_FN_4_O_10_S [M + H]^+^: *m*/*z* = 779.4 observed: *m*/*z* = 779.7.

#### 4.1.30 (2*S*,4*R*)-*N*-(2-((1-((2-(2,6-dioxopiperidin-3-yl)-1,3-dioxoisoindolin-4-yl)amino)-4-oxo-6,9,12-trioxa-3-azatetradecan-14-yl)oxy)-4-(4-methylthiazol-5-yl)benzyl)-1-((*S*)-2-(1-fluorocyclopropane-1-carboxamido)-3,3-dimethylbutanoyl)-4-hydroxypyrrolidine-2-carboxamide (18a)

Starting from compound **17a** (13 mg, 0.0167 mmol) and following the **general method B** the deprotected carboxylic acid derivative was obtained in quantitative yield (12 mg). Starting from the crude carboxylic acid (0.0166 mmol, 1 eq.) and following the general **method H**, the desired compound was obtained (4 mg, yield: 23%). ^1^H NMR (500 MHz, CDCl_3_) δ: 8.65 (s, 1H), 7.47–7.43 (m, 1H), 7.32–7.29 (m, 1H), 7.06 (dd, *J* = 2.4, 7.2 Hz, 1H), 6.99–6.94 (m, 2H), 6.88–6.86 (m, 1H), 4.90–4.79 (m, 1H), 4.65–4.59 (m, 1H), 4.56 (t, *J* = 9.0 Hz, 1H), 4.49–4.39 (m, 3H), 4.22–4.11 (m, 2H), 3.96–3.43 (m, 18H), 2.84–2.61 (m, 3H), 2.50 (d, *J* = 2.7 Hz, 3H), 2.38–2.30 (m, 1H), 2.14–2.04 (m, 2H), 1.37–1.18 (m, 4H), 0.96 (d, *J* = 1.8 Hz, 9H); ^13^C-NMR (126 MHz, CDCl_3_) δ: 171.3, 171.1, 171.0, 170.9, 170.3 170.1, 169.5, 169.0, 168.8, 167.7, 156.8, 150.4, 148.7, 146.9, 136.3, 132.7, 132.5, 131.8, 130.0, 129.8, 127.1, 127.0, 122.3, 122.2, 116.9, 113.0, 112.9, 112.0, 110.5, 79.6, 71.0, 70.8, 70.7, 70.5, 70.3, 70.2, 69.8, 68.1, 59.0, 58.9, 57.6, 56.8, 49.1, 49.1, 42.1, 39.2, 39.2, 38.7, 38.7, 36.8, 35.7, 35.6, 31.6, 26.5, 23.0, 22.9, 16.3, 13.9 (d, *^2^J* = 10.0 Hz),13.8 (d, *^2^J* = 10.0 Hz). HRMS: calculated for C_49_H_62_FN_8_O_13_S[M + H]^+^: *m*/*z* = 1021.4136; observed: *m*/*z* = 1021.4480.

#### 4.1.31 *Tert*-butyl-17-hydroxy-3,6,9,12,15-pentaoxaheptadecanoate (15b)

Starting from pentaethylene glycol (7.32 g, 31 mmol) and following the general method C, the title compound was obtained. Yield: 600 mg (44%). Analytical data matched with those previously reported.^31^

#### 4.1.32 *Tert*-butyl-17-iodo-3,6,9,12,15-pentaoxaheptadecanoate (16b)

Starting from compound **15b** (400 mg, 1.14 mmol) and following the general method D, the title compound was obtained. Yield: 402 mg (81%). ^1^H NMR (500 MHz, CDCl_3_) δ: 4.05 (s, 2H), 3.81–3.69 (m, 18H), 3.29 (t, *J* = 6.9 Hz, 2H), 1.50 (s, 9H). ^13^C NMR (126 MHz, CDCl_3_) δ: 169.7, 81.5, 72.0, 70.8, 70.7, 70.7, 70.6, 70.3, 69.1, 28.1.

#### 4.1.33 17-(2-(((2*S*,4*R*)-1-((*S*)-2-(1-fluorocyclopropane-1-carboxamido)-3,3-dimethylbutanoyl)-4-hydroxypyrrolidine-2-carboxamido)methyl)-5-(4-methylthiazol-5-yl)phenoxy)-3,6,9,12,15-pentaoxaheptadecanoic acid (17b)

Starting from compound **2** (40 mg, 0.075 mmol, 1 eq.), **16b** (42 mg, 0.09 mmol, 1.2 eq.) and K_2_CO_3_ (31 mg, 0.22, 3 eq.), and following the **general method I**, the titled compound was obtained (29 mg, yield: 48%). ^1^H NMR (500 MHz, CDCl_3_) δ: 8.64 (s, 1H), 7.02 (dd, *J* = 3.6, 8.8 Hz, 1H), 6.94 (dd, *J* = 1.6, 7.7 Hz, 1H), 6.87 (d, *J* = 1.4 Hz, 1H), 4.63 (t, *J* = 8.1 Hz, 1H), 4.54–4.42 (m, 4H), 4.22–4.12 (m, 2H), 3.97 (s, 2H), 3.93–3.83 (m, 3H), 3.75–3.60 (m, 17H), 2.49 (s, 3H), 2.39–2.33 (m, 1H), 2.10–2.06 (m, 1H), 1.43 (s, 9H), 1.31–1.20 (m, 4H), 0.93 (s, 9H); ^13^C NMR (101 MHz, CDCl_3_) δ: 170.8, 170.7, 170.0 (d, *^2^J* = 20.3 Hz), 169.8, 157.0, 150.4, 148.6, 132.4, 131.8, 129.9, 127.1, 122.1, 113.0, 81.7, 79.5, 70.9, 70.7, 70.3, 69.8, 69.2, 68.1, 58.8, 57.5, 56.7, 39.2, 36.6, 35.8, 28.2, 26.5, 16.2, 13.7 (t, *^2^J* = 9.6 Hz). MS: calculated for C_42_H_64_FN_4_O_12_S [M + H]^+^: *m*/*z* = 867.4; observed: *m*/*z* = 867.7.

#### 4.1.34 (2*S*,4*R*)-N-(2-((1-((2-(2,6-dioxopiperidin-3-yl)-1,3-dioxoisoindolin-4-yl)amino)-4-oxo-6,9,12,15,18-pentaoxa-3-azaicosan-20-yl)oxy)-4-(4-methylthiazol-5-yl)benzyl)-1-((*S*)-2-(1-fluorocyclopropane-1-carboxamido)-3,3-dimethylbutanoyl)-4-hydroxypyrrolidine-2-carboxamide (18b)

Starting from compound **17b** (29 mg, 0.033 mmol) and following the **general method B** the deprotected carboxylic acid derivative was obtained in quantitative yield (27 mg). Starting from the crude carboxylic acid (27 mg, 0.033, 1 eq.) and following the general **method H**, the desired compound was obtained (4.2 mg, yield: 11%). ^1^H NMR (500 MHz, CDCl_3_) δ: 8.65 (s, 1H), 7.47 (t, *J* = 7.6 Hz, 1H), 7.31 (d, *J* = 7.6 Hz, 1H), 7.07 (dd, *J* = 1.4, 7.0 Hz, 1H), 7.00 (dd, *J* = 1.4, 8.5 Hz, 1H), 6.94 (d, *J* = 7.6 Hz, 1H), 6.87 (d, *J* = 1.5 Hz, 1H), 4.90–4.82 (m, 1H), 4.63 (dt, *J* = 3.2, 7.8 Hz, 1H), 4.55 (d, *J* = 9.1 Hz, 1H), 4.50–4.42 (m, 3H), 4.21–4.10 (m, 2H), 3.95 (s, 2H), 3.95–3.43 (m, 24H), 2.92–2.62 (m, 3H), 2.50 (s, 3H), 2.39–2.31 (m, 1H), 2.15–2.04 (m, 2H), 1.35–1.18 (m, 4H), 0.94 (d, *J* = 1.8 Hz, 9H). ^13^C NMR (500 MHz, CDCl_3_) δ: 171.4, 171.3, 171.2, 170.9, 170.8, 170.1(d,^2^*J* = 20.5 Hz), 169.5, 168.8, 168.7, 167.7, 157.0, 150.4, 148.7, 147.0, 136.3, 132.7, 132.4, 131.8, 130.1, 127.1, 122.2, 117.0, 113.0, 111.9, 110.5, 79.3, 71.1, 70.9, 70.7, 70.6, 70.5, 70.5, 70.2, 69.8, 68.2, 58.9, 57.6, 56.8, 49.1, 42.1, 39.2, 38.6, 36.8, 35.7, 31.6, 26.5, 22.9, 16.3, 13.8 (d,^2^*J* = 10.2 Hz), 13.7 (d,^2^*J* = 10.2 Hz) HRMS: calculated for C_53_H_70_FN_8_O_15_S[M + H]^+^: *m*/*z* = 1109.4660; observed: *m*/*z* = 1110.4625.

#### 4.1.35 Methyl-5-(2-(((2*S*,4*R*)-1-((*S*)-2-(1-fluorocyclopropane-1-carboxamido)-3,3-dimethylbutanoyl)-4-hydroxypyrrolidine-2-carboxamido)methyl)-5-(4-methylthiazol-5-yl)phenoxy)pentanoate (17c)

Starting from compound **2** (40 mg, 0.075 mmol, 1 eq.), methyl 5-bromobutanoate **16c** (21 mg, 0.113 mmol, 1.5 eq.) and K_2_CO_3_ (31 mg, 0.22, 3 eq.), and following the **general method I**, the titled compound was obtained (30 mg, yield: 62%). ^1^H NMR (500 MHz, CDCl_3_) δ: 8.66 (s, 1H), 7.03 (dd, *J* = 3.5, 8.8 Hz, 1H), 6.92 (dd, *J* = 1.5, 7.7 Hz, 1H), 6.82 (d, *J* = 1.5 Hz, 1H), 4.69 (t, *J* = 7.8 Hz, 1H), 4.54–4.45 (m, 3H), 4.38 (dd, *J* = 5.5, 14.9 Hz, 1H), 4.05–3.96 (m, 2H), 3.96–3.91 (m, 1H), 3.64 (s, 3H), 3.61 (dd, *J* = 4.2, 11.2 Hz, 1H), 2.52–2.45 (m, 4H), 2.40 (t, *J* = 6.8 Hz, 2H), 2.09–2.04 (m, 1H), 1.91–1.78 (m, 4H), 1.33–1.24 (m, 4H), 0.91 (s, 9H); ^13^C NMR (101 MHz, CDCl_3_) δ: 173.9, 171.0, 170.3 (d, *^2^J* = 20.5 Hz), 170.2, 156.8, 150.4, 148.6, 132.4, 131.9, 129.7, 126.4, 121.7, 112.1, 79.5, 70.3, 67.7, 58.6, 57.5, 56.6, 51.7, 38.9, 36.0, 35.5, 33.7, 28.8, 26.4, 21.8, 16.2, 13.8 (d,^2^ *J* = 3.7 Hz), 13.7 (d, *^2^J* = 3.7 Hz). MS: calculated for C_32_H_44_FN_4_O_7_S [M + H]^+^: *m*/*z* = 647.3; observed: *m*/*z* = 647.7.

#### 4.1.36 (2*S*,4*R*)-*N*-(2-((5-((2-((2-(2,6-dioxopiperidin-3-yl)-1,3-dioxoisoindolin-4-yl)amino)ethyl)amino)-5-oxopentyl)oxy)-4-(4-methylthiazol-5-yl)benzyl)-1-((*S*)-2-(1-fluorocyclopropane-1-carboxamido)-3,3-dimethylbutanoyl)-4-hydroxypyrrolidine-2-carboxamide (18c)

Compound **17c** (30 mg, 0.046 mmol, 1 eq.) was dissolved in a mixture of THF (2 mL) and water (0.50 mL). Then LiOH was added (2.2 mg, 0.0928 mmol, 2 eq.) and the mixture was stirred at r.t. for 2 h. LC-MS analysis (acidic method) showed complete conversion of the starting material. A solution of HCl 4 N in dioxane was added to pH < 6 and the mixture was evaporated to dryness to yield the deprotected carboxylic acid derivative (25 mg, yield: quantitative). Starting from the crude carboxylic acid (0.023 mmol, 1 eq.) and following the general **method H**, the desired compound was obtained (5 mg, yield: 22%). ^1^H NMR (500 MHz, CDCl_3_) δ: 8.66 (s, 1H), 7.47 (t, *J* = 7.9 Hz, 1H), 7.29 (dd, *J* = 2.2, 7.7 Hz, 1H), 7.07 (d, *J* = 7.2 Hz, 1H), 6.97 (dd, *J* = 3.1, 8.6 Hz, 1H), 6.95–6.94 (m, 1H), 6.85–6.83 (m, 1H), 4.90–4.80 (m, 1H), 4.68 (ddd, *J* = 7.7, 7.7, 1.9 Hz, 1H), 4.55–4.45 (m, 3H), 4.38–4.30 (m, 1H), 4.05–3.93 (m, 3H), 3.60 (qd, *J* = 1.9, 11.2 Hz, 1H), 3.47–3.36 (m, 4H), 2.85–2.60 (m, 3H), 2.50 (s, 3H), 2.47–2.40 (m, 1H), 2.34–2.25 (m, 2H), 2.11–2.02 (m, 2H), 1.90–1.81 (m, 4H), 1.36–1.17 (m, 4H), 0.90 (d, *J* = 3.1 Hz, 9H); ^13^C NMR (126 MHz, CDCl_3_) δ: 173.9, 171.4, 171.2, 170.5, 170.4, 170.3, 169.7, 169.6, 169.0, 168.9, 167.6, 156.9, 150.4, 148.7, 147.0, 136.4, 132.7, 131.8, 130.2, 130.1, 126.2, 121.8, 121.8, 117.0, 112.3, 112.1, 79.4, 70.3, 67.9, 58.8, 57.7, 56.8, 56.7, 49.2, 49.1, 42.3, 39.3, 39.2, 39.1, 36.4, 36.1, 35.5, 31.6, 28.8, 28.7, 26.5, 22.9, 16.3, 13.9 (d, *^2^J* = 3.7 Hz), 13.8 (d, *^2^J* = 3.7 Hz). HRMS analysis: calculated for C_46_H_56_FN_8_O_10_S [M + H]^+^: *m*/*z* = 931.3819; observed: *m*/*z* = 931.3823.

#### 4.1.37 (2*S*,4*R*)-1-((*R*)-2-(1-fluorocyclopropane-1-carboxamido)-3-mercapto-3-methylbutanoyl)-4-hydroxy-*N*-(4-(4-methylthiazol-5-yl)benzyl)pyrrolidine-2-carboxamide (20)

To a solution of compound **19** (0.042 mmol), HATU (16 mg, 0.042 mmol), HOAT (5.71 mg, 0.042 mmol) 1-fluorocyclopropane-1-carboxylic acid (4.3 mg, 0.042 mmol) in DMF (1 mL), DIPEA (25 µL, 0.141 mmol) was added. The reaction mixture was stirred at r.t. for 30 min. LC-MS analysis (acidic method) showed complete conversion of the starting material. Water (1 mL) was added and the resulting mixture was extracted with DCM (3 × 5 mL). After drying the organic phase over MgSO_4_ and the solvent was removed under reduced pressure to afford the title compound (28.3 mg, 85% yield) which was used without further purification.^1^H NMR (400 MHz, CDCl_3_) *δ* 8.71 (s, 1H), 7.54–7.51 (m, 6H), 7.34–7.31 (m, 3H), 7.25–7.19 (m, 12H), 4.69–4.64 (m, 1H), 4.38–4.36 (m, 1H), 4.32–4.19 (m, 2H), 3.66 (d, *J* = 4.2 Hz, 1H), 3.50 (d, *J* = 11.6 Hz, 1H), 3.26 (dd, *J* = 3.9, 11.6 Hz, 1H), 3.09 (d, *J* = 5.9 Hz, 1H), 2.41–2.33 (m, 1H), 2.14–2.07 (m, 1H), 1.38–1.21 (m, 7H), 0.97 (s, 3H). ^19^F NMR: −197.41. MS analysis: calculated for C_44_H_45_FN_4_O_4_S_2_: 776.3; observed: 777.3 [M + H]^+^.

The trityl protected compound (0.04 mmol) was dissolved in 1.8 mL of DCM. TIPS (0.1 mL) and TFA (0.1 mL) were added, and the mixture was left to react at r.t. for 2 h. LC-MS analysis (acidic method) showed complete conversion of the starting material. Volatiles were removed and the crude was dissolved in MeOH, filtered and purified by preparative HPLC and freeze-dried to give pure deprotected compound as white solid (16 mg, 79% yield). ^1^H NMR (500 MHz, CDCl_3_) δ: 8.72 (s, 1H), 7.44 (br.s, 1H), 7.39 (d, *J* = 9.4 Hz, 2H), 7.36 (d, *J* = 7.8 Hz, 2H), 7.15 (br. s, 1H), 4.71 (t, *J* = 7.9 Hz, 1H), 4.64 (d, *J* = 8.5 Hz, 1H), 4.60–4.55 (m, 2H), 4.36 (dd, *J* = 5.3, 14.8 Hz, 1H), 4.15–4.12 (m, 1H), 3.74 (dd, *J* = 3.5, 11.3 Hz, 1H), 2.70 (s, 1H), 2.60 (s, 1H), 2.53–2.46 (m, 4H), 2.20–2.13 (m, 1H), 1.39–1.30 (m, 10*H*). ^19^F NMR: −197. 80. ^13^C NMR (101 mHz, CDCl_3_): 169.7 (d, *^2^J* = 20.7 Hz), 169.4, 169.2, 169.5, 147.3, 137.0, 130.0, 128.7, 127.2, 77.05 (d, *^1^J* = 232.0 Hz), 69.1, 57.9, 56.6, 55.5, 45.0, 42.3, 35.3, 29.5, 27.7, 14.9, 13.0 (d, *^2^J* = 10.6 Hz), 12.8 (d, *^2^J* = 10.5 Hz). MS: calculated for C_25_H_31_FN_4_O_4_S_2_[M + H]^+^: *m*/*z* = 535.2; observed: *m*/*z* = 535.2.

#### 4.1.38 *Tert*-butyl(*R*)-1-(1-fluorocyclopropyl)-3-((2*S*,4*R*)-4-hydroxy-2-((4-(4-methylthiazol-5-yl)benzyl)carbamoyl)pyrrolidine-1-carbonyl)-4,4-dimethyl-1-oxo-8,11,14-trioxa-5-thia-2-azahexadecan-16-oate (21a)

Starting from compound **20** (20 mg, 0.037 mmol, 1 eq.), **16a** (15 mg, 0.041 mmol, 1.1 eq.) and DBU (6,3 µL, 0.041, 1.1 eq.), and following the **general method G**, compound **21a** was obtained (15 mg, yield: 46%). ^1^H NMR (400 MHz, CDCl_3_) δ: 8.64 (s, 1H), 7.33 (dd, *J* = 8.1, 14.2 Hz, 4H), 4.78 (d, *J* = 8.3 Hz, 1H), 4.74 (t, *J* = 7.6 Hz, 1H), 4.53–4.49 (m, 1H), 4.42 (ddt, *J* = 6.0, 13.9, 13.2 Hz, 2H), 3.96 (s, 2H), 3.96–3.91 (m, 1H), 3.81 (dd, *J* = 4.2, 11.1 Hz, 1H), 3.68–3.43 (m, 10*H*), 2.79–2.64 (m, 2H), 2.49 (s, 3H), 2.44–2.36 (m, 1H), 2.23–2.15 (m, 1H), 1.44 (s, 9H), 1.35–1.24 (m, 10*H*); ^13^C NMR (101 MHz, CDCl_3_) δ: 170.8, 170.2 (d, ^2^*J* = 20.7 Hz), 169.8, 169.6, 150.2, 148.5, 138.1, 131.6, 131.0, 129.5, 128.3, 128.2, 81.7, 79.3, 77.3, 77.0, 76.7, 70.7, 70.5, 70.5, 70.4, 70.2, 69.9, 69.0, 59.1, 56.4, 56.0, 47.9, 43.0, 36.8, 28.4, 28.1, 25.8, 25.1, 16.1, 13.9 (d, ^2^*J* = 4.8 Hz),13.8 (d, ^2^*J* = 4.8 Hz). MS: calculated for C_37_H_54_FN_4_O_9_S_2_ [M + H]^+^: *m*/*z* = 781.3; observed: *m*/*z* = 781.8.

#### 4.1.39 (2*S*,4*R*)-1-((17*R*)-1-((2-(2,6-dioxopiperidin-3-yl)-1,3-dioxoisoindolin-4-yl)amino)-17-(1-fluorocyclopropane-1-carboxamido)-16,16-dimethyl-4-oxo-6,9,12-trioxa-15-thia-3-azaoctadecan-18-oyl)-4-hydroxy-*N*-(4-(4-methylthiazol-5-yl)benzyl)pyrrolidine-2-carboxamide (22a)

Starting from compound **21a** (15 mg, 0.019 mmol) and following the **general method B** the deprotected carboxylic acid derivative was obtained in quantitative yield (13.9 mg). Starting from the crude carboxylic acid (0.019, 1 eq.) and following the general **method H**, the desired compound was obtained (11 mg, yield: 56%). ^1^H NMR (400 MHz, MeOD) δ: 8.86 (s, 1H), 7.55 (dd, *J* = 7.2, 8.5 Hz, 1H), 7.46–7.40 (m, 4H), 7.13 (d, *J* = 9.0 Hz, 1H), 7.05 (d, *J* = 7.5 Hz, 1H), 5.04 (dd, *J* = 5.6, 12.8 Hz, 1H), 4.93 (s, 1H), 4.60 (t, *J* = 8.4 Hz, 1H), 4.54–4.35 (m, 3H), 3.95 (s, 2H), 3.88 (d, *J* = 3.1 Hz, 2H), 3.59–3.45 (m, 14H), 2.89–2.65 (m, 5H), 2.47 (s, 3H), 2.28–2.23 (m, 1H), 2.14–2.06 (m, 2H), 1.42–1.25 (m, 10*H*); ^13^C NMR (101 MHz, MeOD) δ: 174.6, 174.0, 173.5, 171.6, 171.5 (d, ^2^*J* = 21.2 Hz) 171.4, 170.8, 170.6, 169.3, 152.8, 149.1, 148.2, 140.2, 137.3, 134.0, 133.4, 131.6, 130.4, 129.0, 118.1, 112.2, 111.6, 79.1 (d, ^1^*J* = 231.9 Hz), 72.0, 71.7, 71.4, 71.3, 71.1, 71.0, 61.1, 58.1, 57.2, 50.2, 43.7, 42.5, 39.5, 39.0, 32.2, 29.6, 27.0, 25.7, 23.8, 15.9, 14.2, 14.1 (t, ^2^*J* = 9.5 Hz). HRMS analysis: calculated for C_48_H_60_FN_8_O_12_S_2_ [M + H]^+^: *m*/*z* = 1023.3751; observed: *m*/*z* = 1023.3730.

#### 4.1.40 *Tert*-butyl(*R*)-1-(1-fluorocyclopropyl)-3-((2*S*,4*R*)-4-hydroxy-2-((4-(4-methylthiazol-5-yl)benzyl)carbamoyl)pyrrolidine-1-carbonyl)-4,4-dimethyl-1-oxo-8,11,14,17,20-pentaoxa-5-thia-2-azadocosan-22-oate (21b)

Starting from compound **20** (20 mg, 0.037 mmol, 1 eq.), **16b** (19 mg, 0.041 mmol, 1.1 eq.) and DBU (6,3 µL, 0.041, 1.1 eq.), and following the **general method G**, the titled compound was obtained (19.4 mg, yield: 66%). ^1^H NMR (400 MHz, CDCl_3_) δ: 8.65 (s, 1H), 7.34 (dd, *J* = 8.3, 13.6 Hz, 4H), 4.81 (d, *J* = 8.5 Hz, 1H), 4.74 (t, *J* = 7.7 Hz, 1H), 4.53–4.50 (m, 1H), 4.42 (ddt, *J* = 5.8, 15.2, 15.3 Hz, 2H), 3.98 (s, 2H), 3.94–3.80 (m, 2H), 3.70–3.42 (m, 18H), 2.80–2.64 (m, 2H), 2.49 (s, 3H), 2.43–2.36 (m, 1H), 2.24–2.15 (m, 1H), 1.44 (s, 9H), 1.33–1.24 (m, 10*H*); ^13^C NMR (101 MHz, CDCl_3_) δ: 171.0, 170.3 (d, ^2^*J* = 20.5 Hz), 169.9, 169.8, 150.4, 148.6, 138.3, 131.7, 131.1, 129.6, 128.3, 81.7, 79.4, 70.9, 70.7, 70.6, 70.3, 70.0, 69.2, 59.3, 56.5, 56.0, 48.1, 43.2, 37.0, 28.6, 28.3, 25.9, 25.2, 16.2, 14.0 (d, ^2^*J* = 2.9 Hz), 13.9 (d, ^2^*J* = 2.9 Hz). MS: calculated for C_41_H_62_FN_4_O_11_S_2_ [M + H]^+^: *m*/*z* = 869.4; observed: *m*/*z* = 869.3.

#### 4.1.41 (2*S*,4*R*)-1-((17*R*)-1-((2-(2,6-dioxopiperidin-3-yl)-1,3-dioxoisoindolin-4-yl)amino)-17-(1-fluorocyclopropane-1-carboxamido)-16,16-dimethyl-4-oxo-6,9,12-trioxa-15-thia-3-azaoctadecan-18-oyl)-4-hydroxy-*N*-(4-(4-methylthiazol-5-yl)benzyl)pyrrolidine-2-carboxamide (22b)

Starting from compound **21b** (19.4 mg, 0.022 mmol) and following the **general method B** the deprotected carboxylic acid derivative was obtained in quantitative yield (17 mg, yield: quantitative). Starting from the crude carboxylic acid (0.011 mmol, 1 eq.) and following the general **method H**, the desired compound was obtained (7.3 mg, yield: 47%). ^1^H NMR (500 MHz, MeOD) δ: 8.86 (s, 1H), 7.55 (dd, *J* = 7.2, 8.6 Hz, 1H), 7.44 (dd, *J* = 8.5, 11.4 Hz, 4H), 7.14 (d, *J* = 8.6 Hz, 1H), 7.05 (d, *J* = 7.0 Hz, 1H), 5.04 (dd, *J* = 5.4, 12.4 Hz, 1H), 4.94 (s, 1H), 4.61 (t, *J* = 8.4 Hz, 1H), 4.52–4.37 (m, 3H), 3.96 (s, 2H), 3.92–3.86 (m, 2H), 3.57–3.47 (m, 22H), 2.88–2.66 (m, 5H), 2.48 (s, 3H), 2.28–2.24 (m, 1H), 2.14–2.07 (m, 2H), 1.42–1.27 (m, 10*H*); ^13^C NMR (126 MHz, MeOD) δ: 174.7,174.1, 173.6, 171.5 (d, ^2^*J* = 19.8 Hz) 171.5, 170.8, 170.6, 169.2, 152.9, 149.1, 148.1, 140.2, 137.3, 134.0, 133.4, 131.6, 130.5, 130.4, 129.6, 129.0, 118.1, 112.1, 111.5, 79.1 (d, ^1^*J* = 231.2 Hz), 72.0, 71.6, 71.6, 71.5, 71.4, 71.3, 71.1, 71.0, 61.1, 58.1, 57.1, 43.6, 42.5, 39.4, 39.1, 32.2, 29.6, 26.9, 25.7, 23.8, 15.9, 14.2 (d, ^2^*J* = 7.9 Hz), 14.1 (d, ^2^*J* = 7.9 Hz). HRMS: calculated for C_52_H_68_FN_8_O_14_S_2_ [M + H]^+^: *m*/*z* = 1111.4275; observed: *m*/*z* = 1111.4052.

#### 4.1.42 Methyl-5-(((*R*)-3-(1-fluorocyclopropane-1-carboxamido)-4-((2*S*,4*R*)-4-hydroxy-2-((4-(4-methylthiazol-5-yl)benzyl)carbamoyl)pyrrolidin-1-yl)-2-methyl-4-oxobutan-2-yl)thio)pentanoate (21c)

Starting from compound **20** (20 mg, 0.037 mmol, 1 eq.), methyl 5-bromobutanoate **16c** (8 mg, 0.041 mmol, 1.1 eq.) and DBU (6,3 µL, 0.041, 1.1 eq.), and following the **general method G**, compound **21c** was obtained (16.5 mg, yield: 67%). ^1^H NMR (400 MHz, CDCl_3_) δ: 8.65 (s, 1H), 7.33 (dd, *J* = 8.0, 16.3 Hz, 4H), 4.76–4.71 (m, 2H), 4.50 (t, *J* = 6.9 Hz, 1H), 4.42 (d, *J* = 6.1 Hz, 2H), 3.97 (d, *J* = 11.8 Hz, 1H), 3.74–3.69 (m, 1H), 3.62 (s, 3H), 2.54–2.37 (m, 6H), 2.24 (t, *J* = 7.6 Hz, 2H), 2.21–2.16 (m, 1H), 1.67–1.58 (m, 2H), 1.52–1.42 (m, 2H), 1.32–1.24 (m, 10*H*); ^13^C NMR δ: (101 MHz, CDCl_3_) *δ* 173.8, 170.9, 170.5 (d, ^2^*J* = 20.7 Hz), 170.0, 150.5, 148.6, 138.2, 131.7, 131.1, 129.6, 128.2, 79.4, 70.2, 59.2, 56.7, 56.2, 51.7, 48.1, 43.2, 36.9, 33.6, 28.9, 28.0, 25.8, 25.4, 24.4, 16.1, 14.0 (d, ^2^*J* = 3.4 Hz), 13.9 (d, ^2^*J* = 3.4 Hz). MS: calculated for C_31_H_42_FN_4_O_6_S_2_ [M + H]^+^: *m*/*z* = 649.3; observed: *m*/*z* = 649.3.

#### 4.1.43 (2*S*,4*R*)-1-((2*R*)-3-((4-((2-((2-(2,6-dioxopiperidin-3-yl)-1,3-dioxoisoindolin-4-yl)amino)ethyl)amino)-4-oxobutyl)thio)-2-(1-fluorocyclopropane-1-carboxamido)-3-methylbutanoyl)-4-hydroxy-*N*-(4-(4-methylthiazol-5-yl)benzyl)pyrrolidine-2-carboxamide (22c)

Compound **21c** (16.5 mg, 0.025 mmol, 1 eq.) was dissolved in a mixture of THF (1 mL) and water (0.25 mL). Then LiOH was added (1.2 mg, 0.0509 mmol, 2 eq.) and the mixture was stirred at r.t. for 2 h. LC-MS analysis (acidic method) showed complete conversion of the starting material. A solution of HCl 4 N in dioxane was added to pH < 6 and the mixture was evaporated to dryness to yield the deprotected carboxylic acid derivative (16.1 mg, yield: quantitative). Starting from the crude carboxylic acid (0.025 mmol, 1 eq.) and following the general **method H**, the desired compound was obtained (9.2 mg, yield: 39%). ^1^H NMR (500 MHz, MeOD) δ: 8.92 (s, 1H), 7.54 (dd, *J* = 7.2, 8.5 Hz, 1H), 7.46–7.39 (m, 4H), 7.07 (dd, *J* = 7.8, 23.5 Hz, 2H), 5.04 (dd, *J* = 5.6, 12.9 Hz, 1H), 4.91 (d, *J* = 9.2 Hz, 1H), 4.61 (t, *J* = 8.3 Hz, 1H), 4.53–4.36 (m, 3H), 3.90–3.82 (m, 2H), 3.48–3.37 (m, 4H), 2.89–2.68 (m, 3H), 2.56 (t, *J* = 7.4 Hz, 2H), 2.48 (s, 3H), 2.29–2.22 (m, 1H), 2.14–2.08 (m, 4H), 1.67–1.55 (m, 2H), 1.51–1.43 (m, 2H), 1.41–1.25 (m, 10*H*); ^13^C NMR (126 MHz, MeOD) δ: 176.4, 174.6, 174.1, 171.5, 170.9, 170.6, 169.3, 153.0, 148.7, 148.2, 140.3, 137.2, 134.0, 133.6, 131.4, 130.5, 130.4, 129.5, 129.0, 118.0, 112.1, 111.5, 79.1 (d, ^1^*J* = 232.0 Hz) 71.0, 61.1, 58.1, 57.3, 57.2, 43.7, 42.8, 39.8, 39.0, 36.4, 32.2, 30.2, 29.0, 27.1, 26.2, 25.7, 23.8, 15.7, 14.2, 14.1 (dd, ^2^*J* = 9.1 Hz). HRMS: calculated for C_45_H_53_FN_8_O_9_S_2_ [M + H]^+^: *m*/*z* = 933.3434; observed: *m*/*z* = 933.3263.

### 4.2 Biology

#### 4.2.1 Cell culture

HeLa (CCL-2) and HEK293 (CRL-1573) cells were purchased from ATCC and cultured in DMEM medium (Gibco) supplemented with 10% FBS, 100 µg/mL penicillin/streptomycin and l-glutamine. Cells were grown at 37 °C and 5% CO_2_, and were propagated no longer than 30 passages. All cell lines were routinely tested for mycoplasma contamination using MycoAlert kit from Lonza.

#### 4.2.2 Evaluation of cellular activity of PROTACs

HeLa (5 × 10^5^) and HEK293 (1 × 10^6^) cells were seeded in standard 6-well plates (2 mL medium) overnight before treatment with compounds at the desired concentration and a final DMSO concentration of 0.1% v/v. After the appropriate incubation time, cells were washed with DPBS (Gibco) and lysed using 85 µL RIPA buffer (Sigma-Aldrich) supplemented with cOmplete Mini EDTA-free protease inhibitor cocktail (Roche) and benzonase. Lysates were clarified by centrifugation (20000 *g*, 10 min, 4 °C) and the total protein content of the supernatant was quantified using a Bradford colorimetric assay. Samples were prepared using LDS sample buffer (Invitrogen) and equal amounts of total protein.

#### 4.2.3 Immunoblotting

Proteins were separated by SDS-PAGE on NuPage 4–12% Bis-Tris gels and transferred to Amersham Protran 0.45 NC nitrocellulose membranes (GE Healthcare) using wet transfer. Membranes were blocked using 5% w/v milk in Tris-buffered saline (TBS) with 0.1% Tween-20. Blots were probed using anti-VHL (CST-68547), anti-CRBN (Novus, NBP1-91810) and anti-β-tubulin hFAB-rhodamine (BioRad, 12004166) primary antibodies, followed by incubation with secondary anti-Rabbit IRDye 800CW (ab216773) or anti-rabbit HRP-conjugated (CST-7074) antibodies. Blots were developed using a Bio-Rad ChemiDoc MP Imaging System or the Amersham ECL Prime western blotting detection kit and Amersham Hyperfilm ECL film, as appropriate. Band quantification was performed using the ImageJ software. Band intensities were normalized to the β-tubulin loading control and reported as % of the average 0.1% DMSO vehicle intensity. Degradation data was plotted and analysed using Prism (Graphpad, version 7). DC_50_ values (concentration to reach 50% maximal degradation) were estimated by fitting band intensity against log[concentration]. Apparent half-life values (time to reach 50% maximal degradation) were estimated by fitting band intensity against time using a single-phase exponential decay model.
