# Supplementary material for: Cereblon versus VHL: Hijacking E3 ligases against each other using PROTACs
Source: Bioorg Med Chem. 2019 Jun 15;27(12):2466–79. doi: 10.1016/j.bmc.2019.02.048 (PMC6561380; doi:10.1016/j.bmc.2019.02.048)
Supplement: Supplementary data 1 [file mmc1.pdf]

Cereblon versus VHL: Hijacking E3 ligases against each other using PROTACs

Miriam Girardini[a,1], Chiara Maniaci[a,1,2], Scott J. Hughes[a,1], Andrea Testa[a], Alessio Ciulli[a,*]

[a]*Division of Biological Chemistry and Drug Discovery, School of Life Sciences, University of Dundee, Dow Street, Dundee DD1 5EH, Scotland, United Kingdom*

[1] These authors contributed equally to this work

[2] Current address: University of Oxford, Chemistry Research Laboratory, 12 Mansfield Road, Oxford OX1 3TA, United Kingdom

*Corresponding author: a.ciulli@dundee.ac.uk

Supporting Information

Table of Contents

Supporting Figures 2

Supporting Tables 4

Supporting Schemes 6

Supporting synthetic procedures 7

Supporting NMR spectra 10

Analytical HPLC traces of final compounds 23

Supporting References 26

Supporting Figures

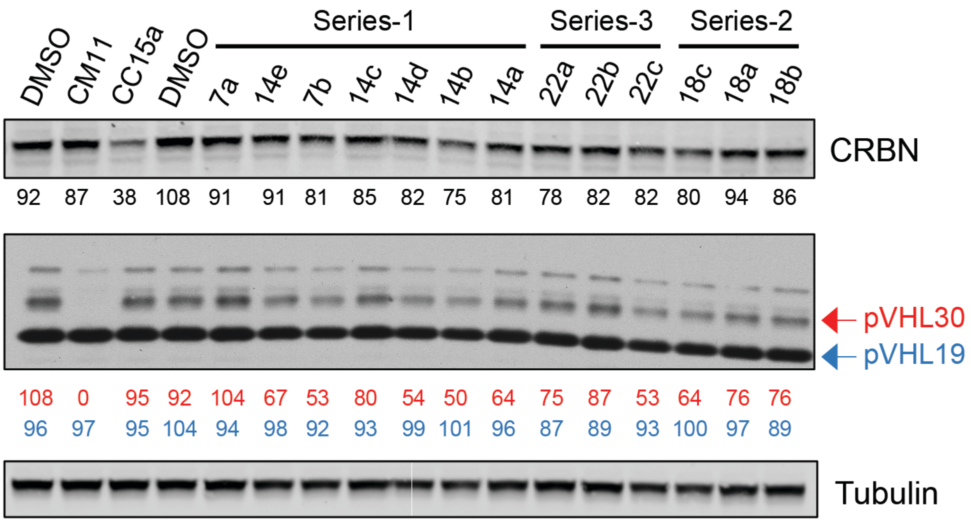

Figure S1. Screening of VHL-CRBN hetero-PROTACs. Western blot analysis of CRBN and VHL levels following 4 h treatment of HeLa cells with 10 nM compound. Values reported below each lane indicate protein abundance relative to the average 0.1% DMSO vehicle.

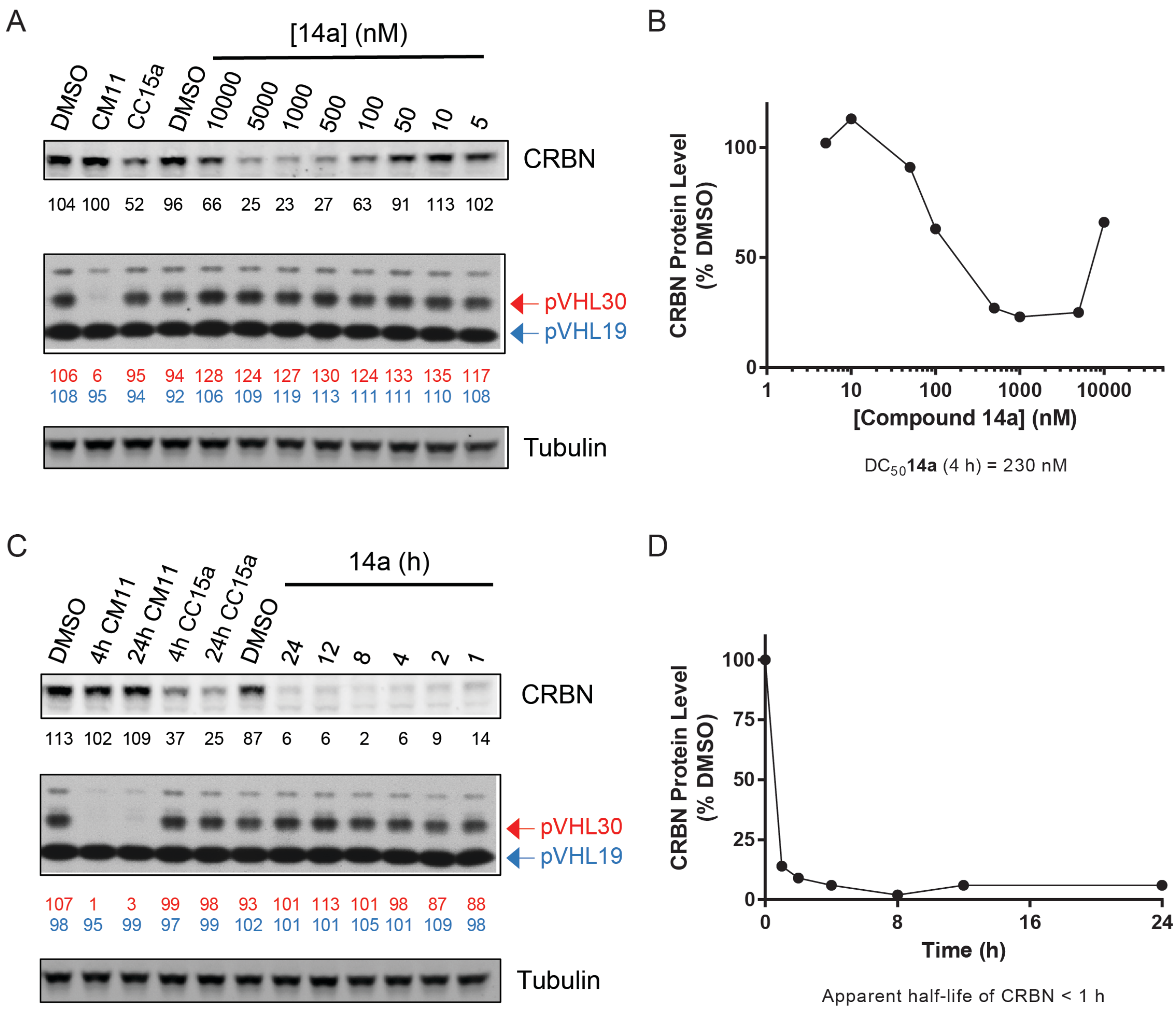

Figure S2. Compound 14a induces rapid depletion of CRBN, but not of VHL. **(A)** Western blot analysis of CRBN and VHL levels following 4 h treatment of HEK293 cells with the indicated concentrations of **14a**. **(B)** Quantification of CRBN levels following concentration-dependent assessment. **(C)** Western blot analysis of CRBN and VHL levels following treatment of HEK293 cells with 1 μM **14a** for the indicated time points. **(D)** Quantification of CRBN levels following treatment with 1 μM **14a** for 6 time points. Values reported below each lane indicate protein abundance relative to the average 0.1% DMSO vehicle. DC_{50} and half-lives were determined as described in the Experimental Section.

Supporting Tables

Table S1. Chemical structures of the CBRN-VHL PROTACs studied in this work.

Compound	Chemical structure
**7a**	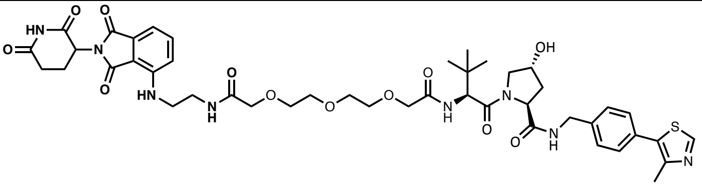
**14e**	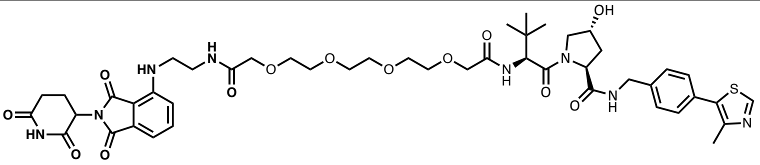
**7b**	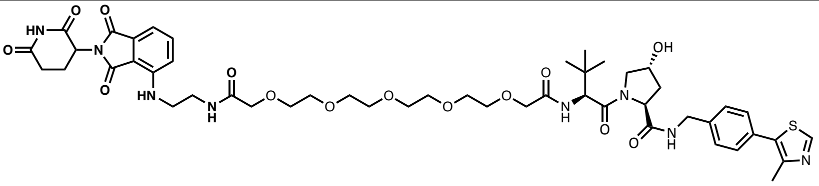
**14c**	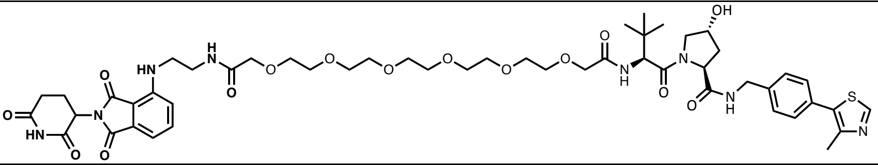
**14d**	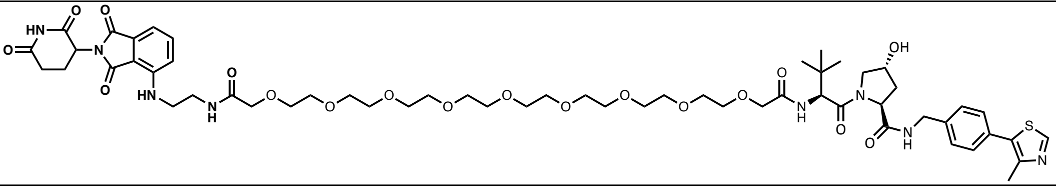
**14b**	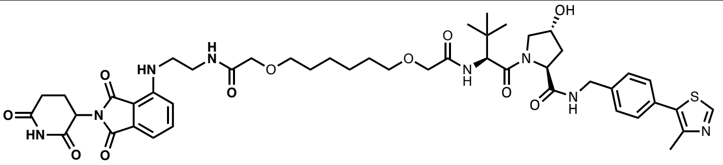
**14a**	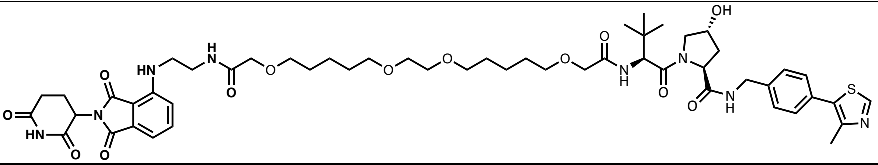
**18a**	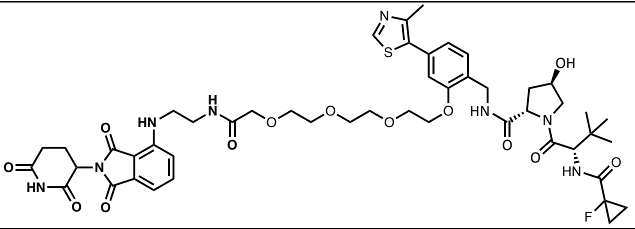
**18b**	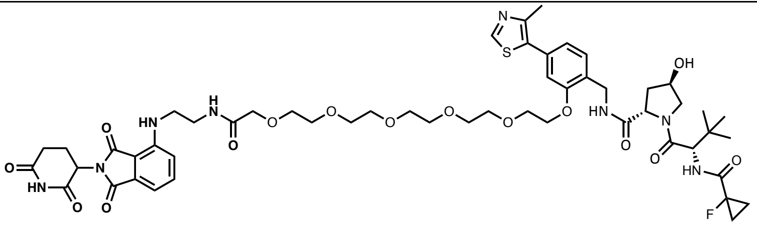

**18c**	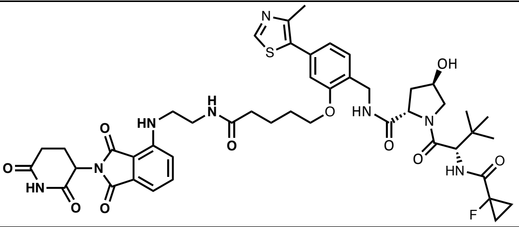
**22a**	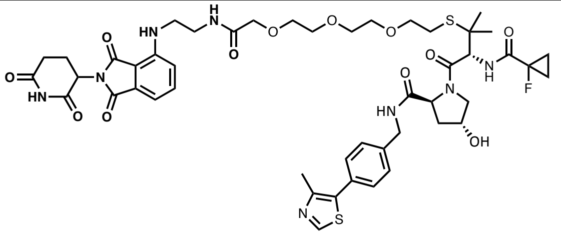
**22b**	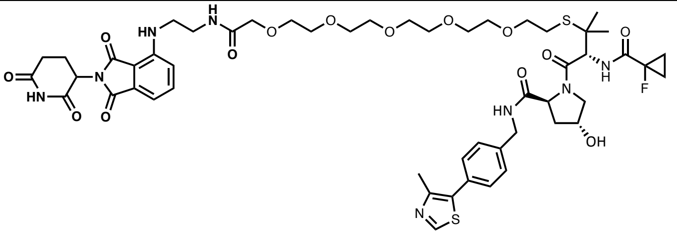
**22c**	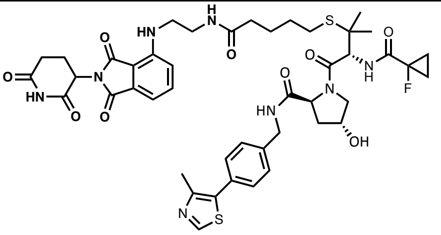

Supporting Schemes

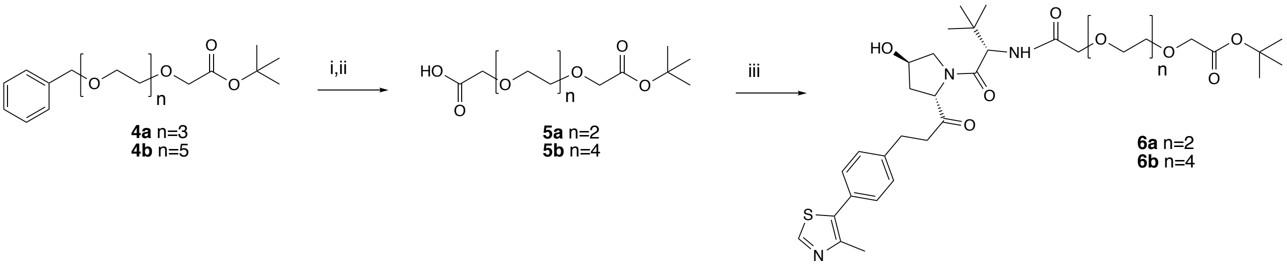

SI Scheme 1: Synthesis of compounds **6a-b**. i. H_2, Pd/C, ethanol, r.t; ii. BIAB, TEMPO, ACN/H_2O 1:1, r.t; iii. HATU, HOAt, **1**, DIPEA, DMF, r.t.

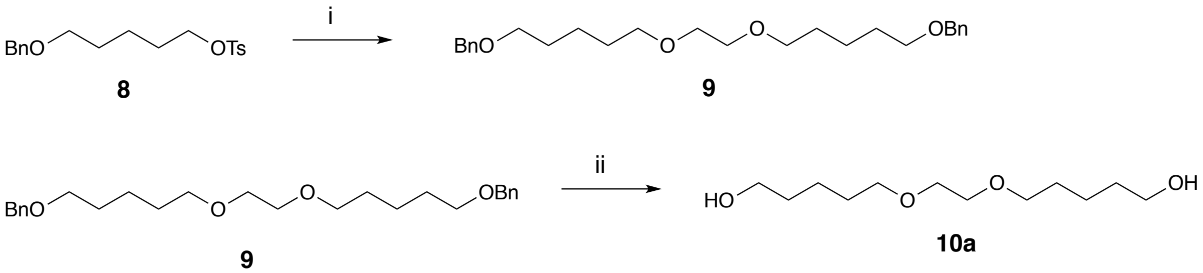

SI Scheme 2: Synthesis of diol **10a**. i. ethylene glycol, NaH 60% dispersion in mineral oil, DMF dry, r.t to 50°C, O/N, 79%; ii. H_2, Pd/C, ethanol, r.t., quantitative.

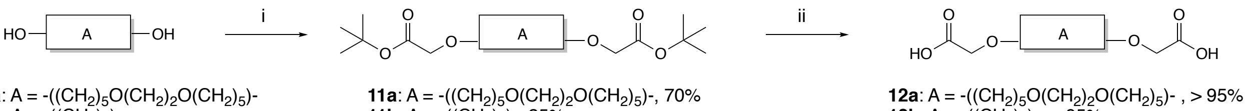

10a: A = -((CH_2)$_5$O(CH_2)$_2$O(CH_2)$_5$)-
10b: A = -((CH_2)$_6$)-
10c: A = -(CH_2CH_2(OCH_2CH_2)$_4$)-
10d: A = -(CH_2CH_2(OCH_2CH_2)$_7$)-
10e: A = -(CH_2CH_2(OCH_2CH_2)$_2$)-

11a: A = -((CH_2)$_5$O(CH_2)$_2$O(CH_2)$_5$)-, 70%
11b: A = -((CH_2)$_6$)-, 25%
11c: A = -(CH_2CH_2(OCH_2CH_2)$_4$)-, 76%
11d: A = -(CH_2CH_2(OCH_2CH_2)$_7$)-, 74%
11e: A = -(CH_2CH_2(OCH_2CH_2)$_2$)-,19%

12a: A = -((CH_2)$_5$O(CH_2)$_2$O(CH_2)$_5$)- , > 95%
12b: A = -((CH_2)$_6$)-, > 95%
12c: A = -(CH_2CH_2(OCH_2CH_2)$_4$)-, > 95%
12d: A = -(CH_2CH_2(OCH_2CH_2)$_7$)-, > 95%
12e: A = -(CH_2CH_2(OCH_2CH_2)$_2$)-, > 95%

SI Scheme 3: Synthesis of linkers **12a-e**. i. *Tert*-butyl bromoacetate, TBABr, NaOH 37%, DCM, r.t, overnight; ii. 1:1 TFA/DCM, r.t, 1h.

Supporting synthetic procedures

tert-butyl 1-phenyl-2,5,8,11-tetraoxatridecan-13-oate **(4a)**

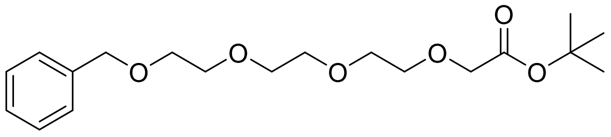

Compound **4a** was prepared as previously reported.[1]

13,13-dimethyl-11-oxo-3,6,9,12-tetraoxatetradecanoic acid (5a)

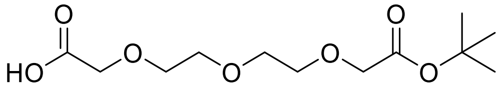

Compound **4a** (551 mg, 1.56 mmol, 1 eq.) was dissolved in 10 mL of ethanol, Pd/C (10 wt %) was added and the resulting mixture was place under hydrogen and stirred at room temperature until complete conversion of the starting material was observed by TLC (100% ethyl acetate). The reaction mixture was filtered through celite and the celite pad washed few times using ethanol. The filtrate was concentrated in vacuum to give an oil (381 g, yield: 93%). BAIB (1.01 g, 3.16 mmol, 2.2 eq.) and TEMPO (49 mg, 0.32 mmol, 0.22 eq.) were added to a solution of ACN/H_2O 1:1 (3 mL) containing the previous obtained oil (380 mg, 1.44 mmol, 1 eq.) The resulting mixture was stirred at room temperature until complete conversion of the starting material was observed by TLC (100% ethyl acetate). The crude was purified using ISOLUTE® PE-AX anion exchange column equilibrated with methanol. The reaction mixture was poured on the column and let it adsorb in the pad. The column was washed with methanol (×3) to elute all the unbound material. Then, the titled product was eluted using a 5% solution of formic acid in methanol. The organic phase was evaporated to dryness to afford the title compound as oil (210 mg, yield: 52%). ^{1}H-NMR (400 MHz, $CDCl_3$) δ 4.09 (s, 2H), 3.95 (s, 2H), 3.72 - 3.64 (m, 8H), 1.41 (s, 9H).

5-(Benzyloxy)pentyl 4-methylbenzenesulfonate **(8)**

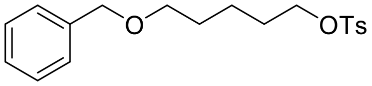

Compound **8** was prepared as previously reported. Analytical data matched those previously reported.[2]

5,5'-(ethane-1,2-diylbis(oxy))bis(pentan-1-ol) **(9)**

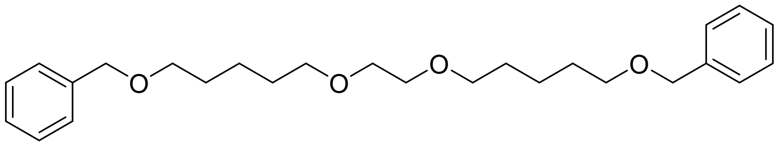

A solution of ethylenglycol (178 mg, 160 μL, 2.87 mmol, 1 eq.) and DMF dry (24.5 mL) was chilled to 0°C. NaH 60% dispersion in mineral oil (459 mg, 11.5 mmol, 4 eq.) was added and the mixture was left to stir for 1h at room temperature. Then, compound **8** (2.0 g, 5.7396 mmol, 2 eq.) was added and the mixture was stirred at 50°C overnight. After reaction completion (TLC monitoring, 30% ethyl acetate in heptane), NH_4Cl (saturated solution) was added to pH=7, the mixture was dried under reduced pressure and partitioned between water and DCM. The aqueous phase was extracted with DCM (x2) and the organic layers were collected, dried over $MgSO_4$ and purified by flash chromatography eluting from 0% to 30% v/v ethyl acetate in heptane to yield the desired product (700 mg, yield: 79%).^{1}H-NMR (400 MHz, $CDCl_3$) δ: 7.35 - 7.23 (m,

10H), 4.47 (s, 4H), 3.54 (s, 4H), 3.45 (t, *J*=6.5 Hz, 4H), 3.44 (t, *J*=6.5 Hz, 4H), 1.66 - 1.54 (m, 8H), 1.44 - 1.36 (m, 4H).

***5,5'-(ethane-1,2-diylbis(oxy))bis(pentan-1-ol)* (10a)**

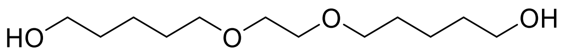

Compound **9** (500 mg, 1.21 mmol) was dissolved in EtOH (15 mL) in a dried round bottom flask under nitrogen atmosphere. Pd/C was added and the flask was evacuated and backfield with argon. The flask was evacuated again before a hydrogen balloon was connected to the reaction mixture. The reaction mixture was stirred for 6h. Complete conversion of the starting material was observed by TLC (30 % ethyl acetate in heptane). The reaction mixture was filtered on celite and dried under reduced pressure. The product was obtained in quantitative yield without any further purification (268 mg). ^{1}H-NMR (500 MHz, $CDCl_3$) δ: 3.45 (t, *J*=6.6 Hz, 4H), 3.43 (s, 4H), 3.34 (t, *J*=6.6 Hz, 4H), 1.50 - 1.40 (m, 8H), 1.31 - 1.21 (m, 4H);^{13}C-NMR (101 MHz, $CDCl_3$) δ: 71.2, 70.0, 62.1, 32.3, 29.2, 22.3.

***Di-tert-butyl 3,9,12,18-tetraoxaicosanedioate* (11a)**

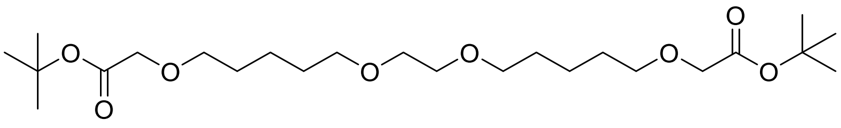

Starting from compound **10a** (118 mg, 0.50 mmol), tert-butyl bromoacetate (786 mg, 4.00 mmol, 8 eq.), TBABr (184 mg, 0.55 mmol, 1.1 eq.) and following the **general method A,** compound **11a** was obtained (168 mg, yield 70%), after flash chromatography eluting from 0% to 50% v/v ethyl acetate in heptane.^{1}H-NMR (500 MHz, $CDCl_3$) δ: 3.87 (s, 4H), 3.49 (s, 4H), 3.44 (t, *J*=6.7 Hz, 4H), 3.39 (t, *J*=6.7 Hz, 4H), 1.61 - 1.50 (m, 8H), 1.41 (s, 18H), 1.39 - 1.31 (m, 4H).

***Di-tert-butyl 2,2'-(hexane-1,6-diylbis(oxy))diacetate* (11b)**

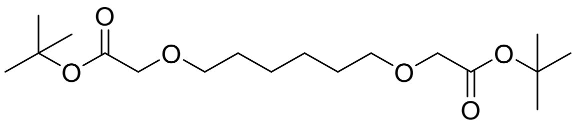

Starting from the commercially available 1,6-hexandiol (250 mg, 2.2 mmol), tert-butyl bromoacetate (3.3 g, 17.6 mmol, 8 eq.), TBABr (750 mg, 2.4 mmol, 1.1 eq.) and following the **general method A** the titled compound was obtained (185 mg, yield 25%), after flash chromatography eluting from 0% to 10% v/v ethyl acetate in heptane.^{1}H-NMR (400 MHz, $CDCl_3$) δ: 3.92 (s, 4H), 3.49 (t, *J*=6.6 Hz, 4H), 1.64 - 1.56 (m, 4H), 1.46 (s, 18H), 1.41 - 1.34 (m, 4H).

***di-tert-butyl 3,6,9,12,15,18-hexaoxaicosanedioate* (11c)**

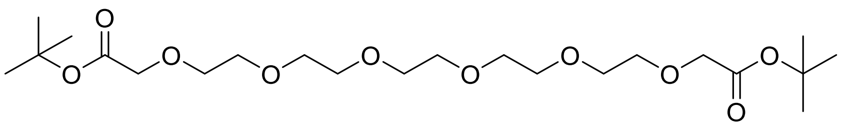

Starting from the commercially available pentaethylene glycol **10c** (201 mg, 0.84 mmol), tert-butyl bromoacetate (1.318g, 6.7 mmol, 8 eq.), TBABr (308 mg, 0. 92 mmol, 1.1 eq.) and following the **general method A** compound **11c** was obtained (300 mg, yield 76%), after flash chromatography eluting from 0% to 20% v/v methanol in dichloromethane. Analytical data matched those previously reported.[3]

***Di-tert-butyl 3,6,9,12,15,18,21,24,27-nonaoxanonacosanedioate* 11d**

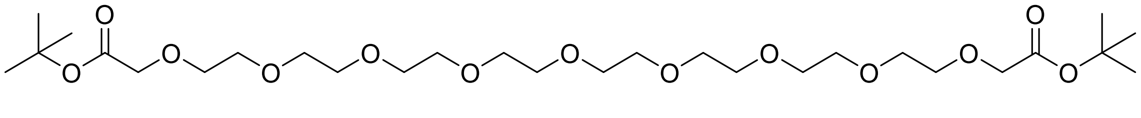

Starting from commercially available octaethylene glycol **10d**, (200 mg, 0.54 mmol) tert-butyl bromoacetate (842 mg, 4.32 mmol, 8 eq.), TBABr (191 mg, 0.59 mmol, 1.1 eq.) and following the **general method A** compound **11d** was obtained (240 mg, yield 74%), after flash chromatography eluting from 0% to 20% v/v methanol in dichloromethane.^{1}H-NMR (400 MHz, $CDCl_3$) δ: 4.02 (s, 4H), 3.73 - 3.64 (m, 32H), 1.48 (s, 18H);^{13}C-NMR (101 MHz, $CDCl_3$) δ: 169.7, 81.5, 70.8, 70.7, 69.2, 28.2.

Supporting NMR spectra

7a ^{1}H-NMR (500 MHz, CDCl3)

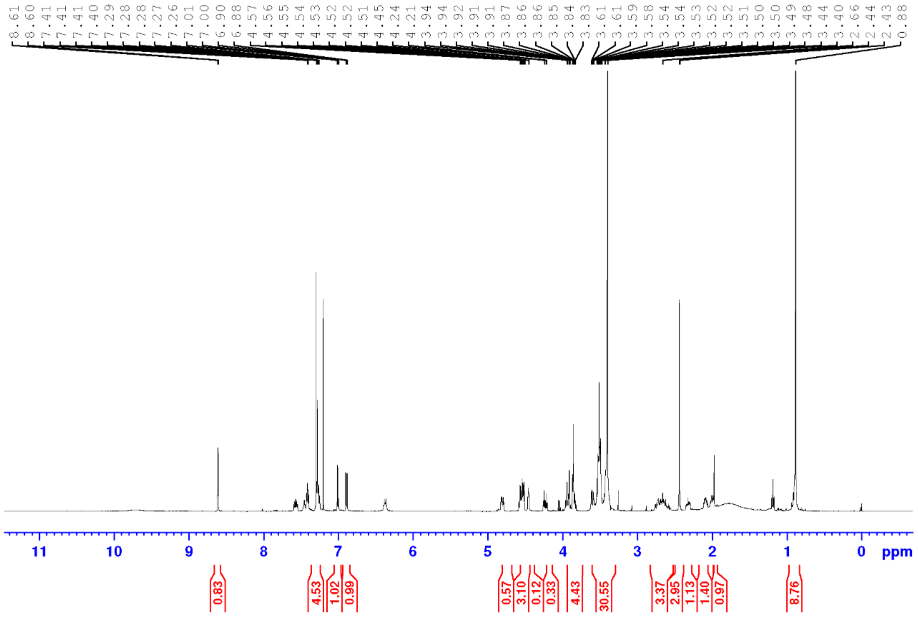

7a ^{13}C-NMR (500 MHz, CDCl3)

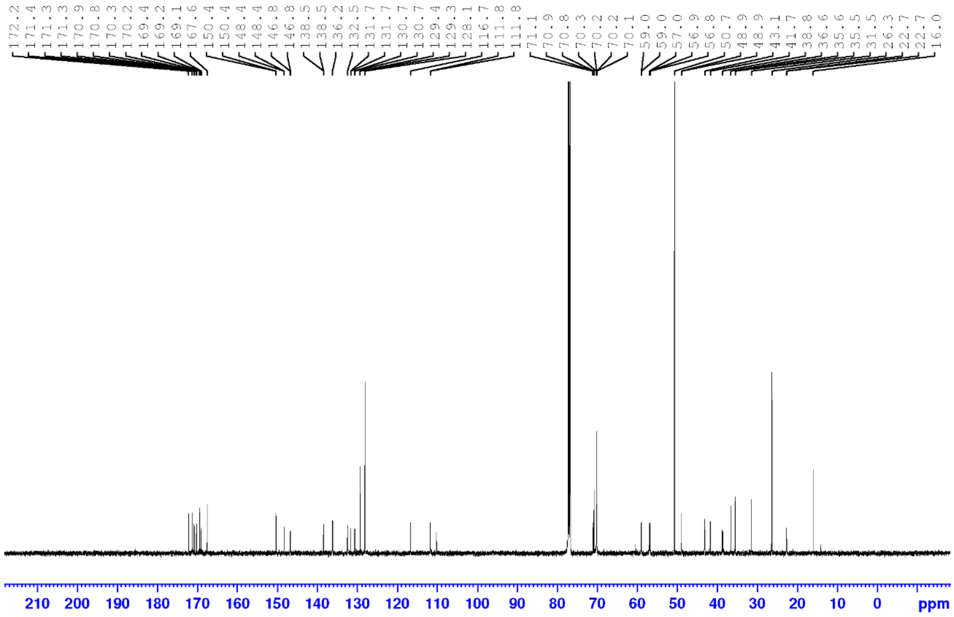

7b ^{1}H-NMR (500 MHz, CDCl3)

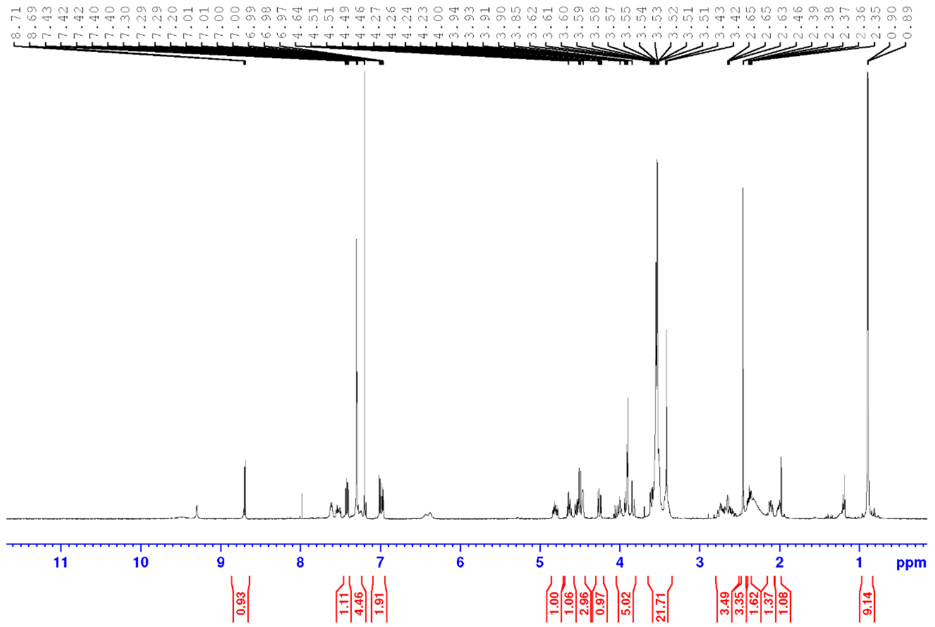

7b ^{13}C-NMR (500 MHz, CDCl3)

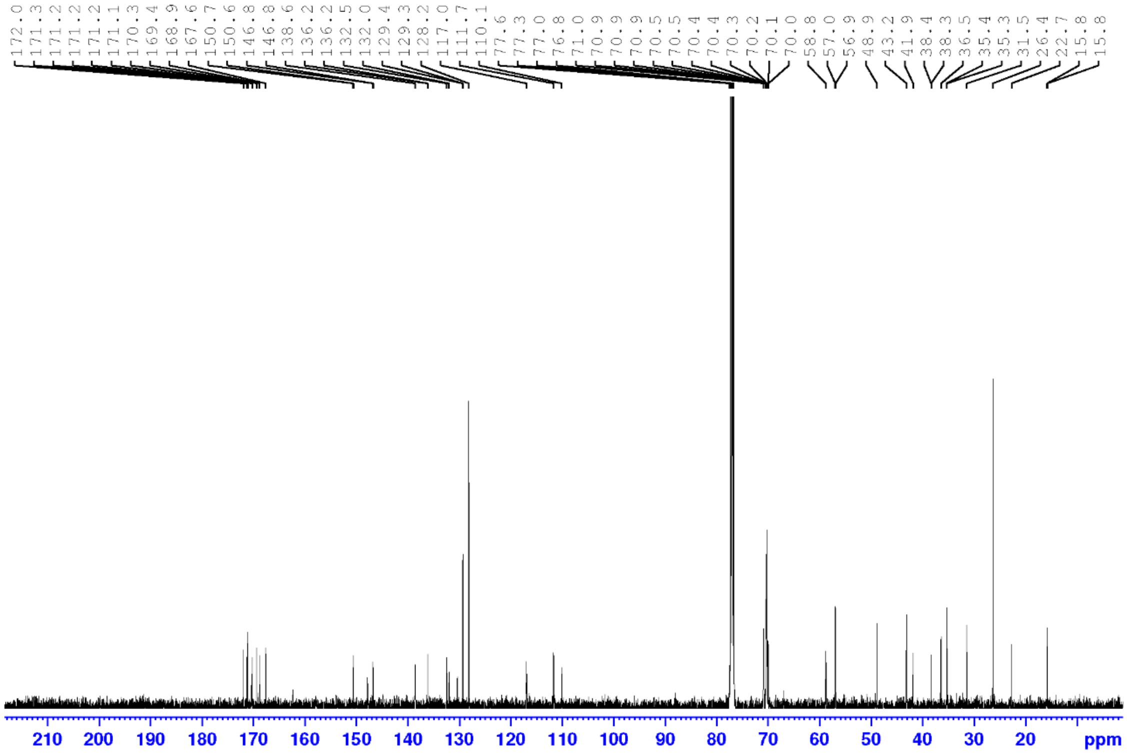

14a ^{1}H-NMR (500 MHz, MeOD)

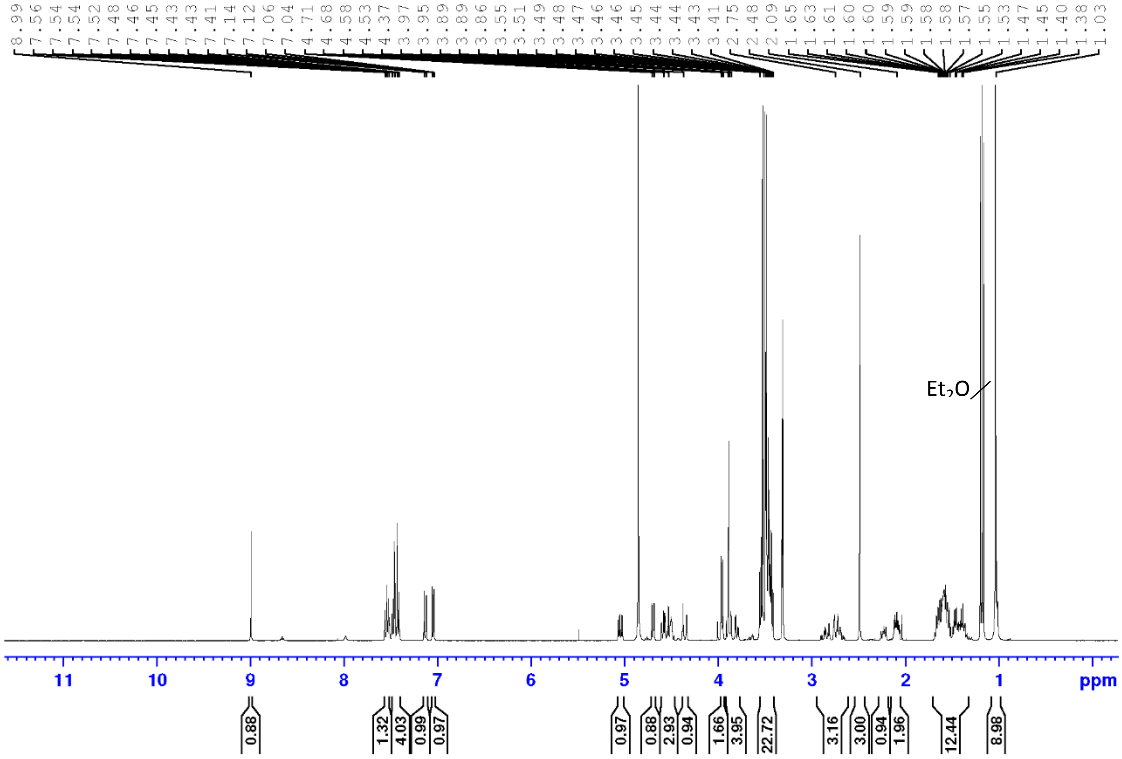

14a ^{13}C-NMR (101 MHz, MeOD)

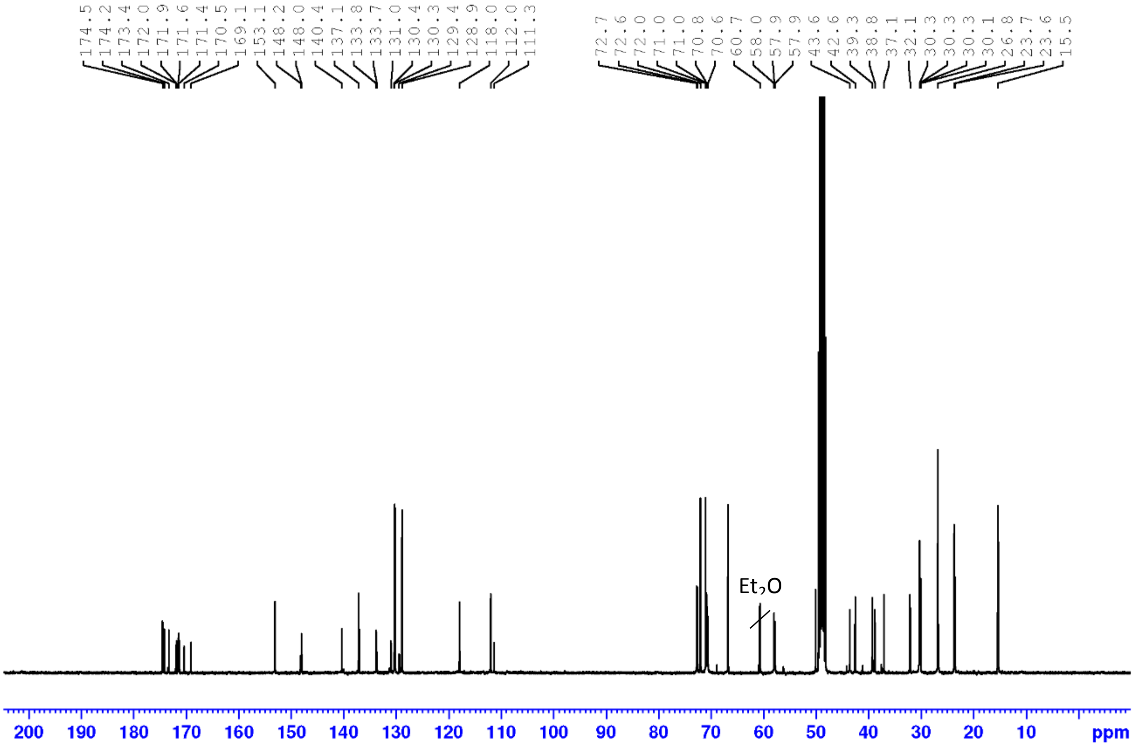

14b ^{1}H-NMR (400 MHz, MeOD)

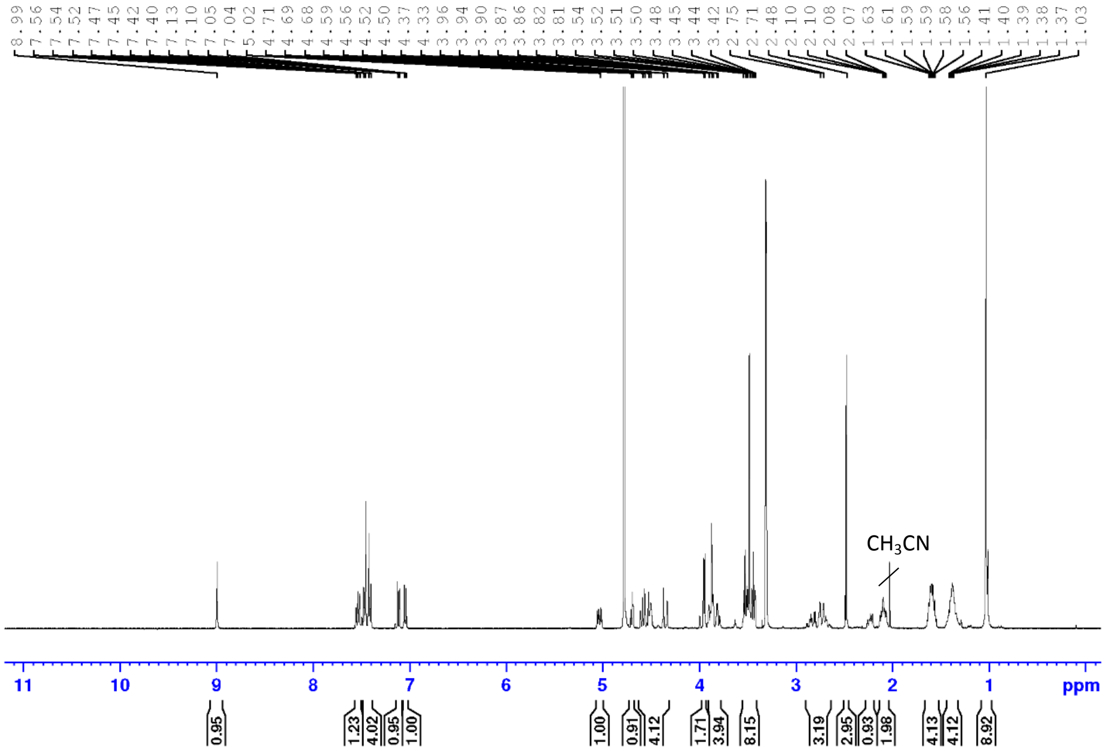

14b ^{13}C-NMR (101 MHz, MeOD)

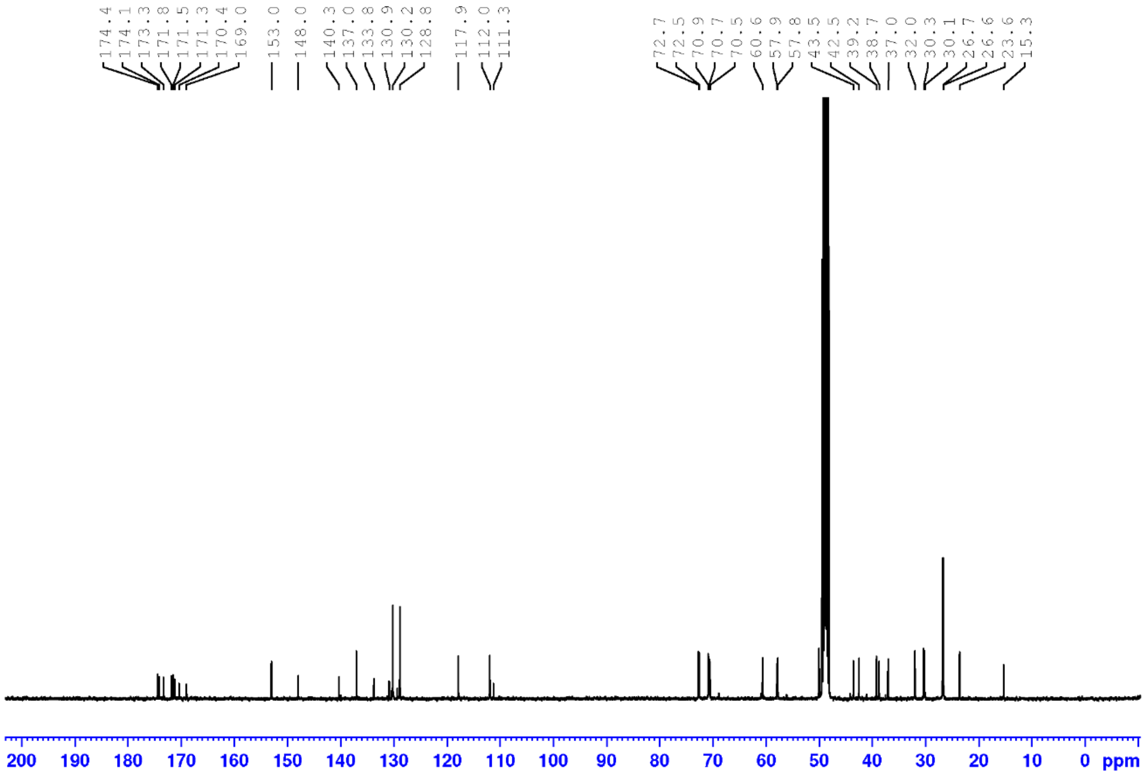

14c ^{1}H-NMR (400 MHz, MeOD)

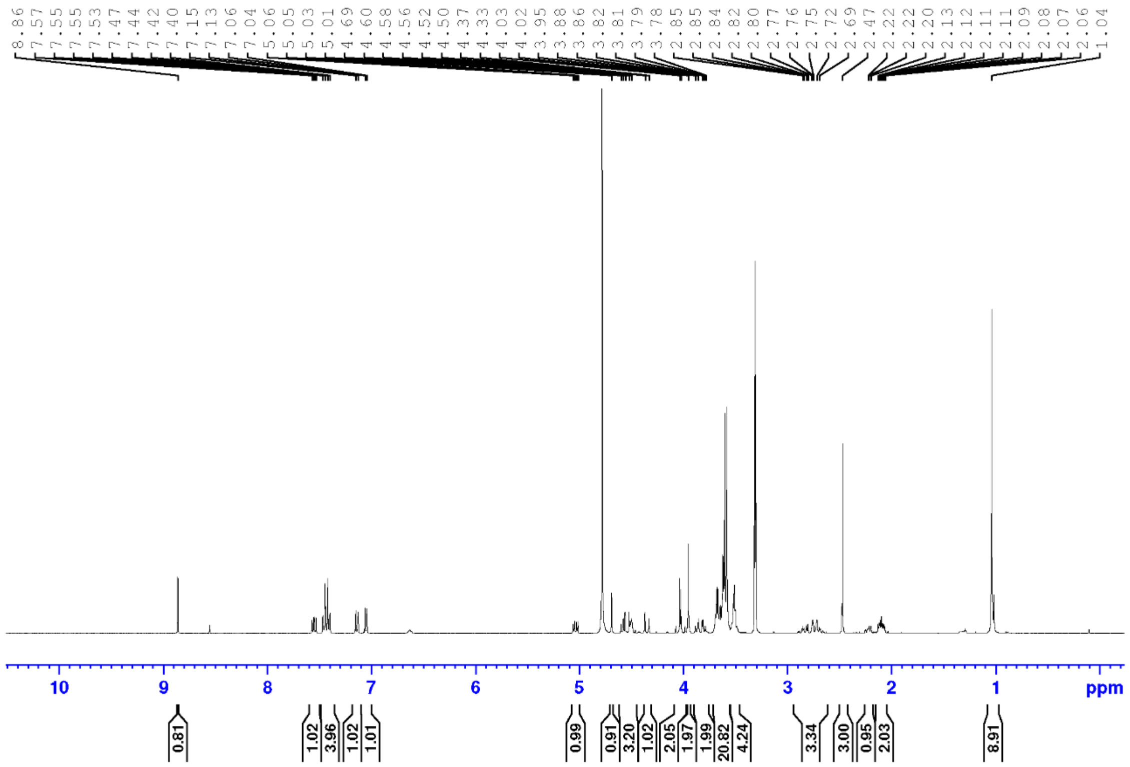

14c ^{13}C-NMR (101 MHz, MeOD)

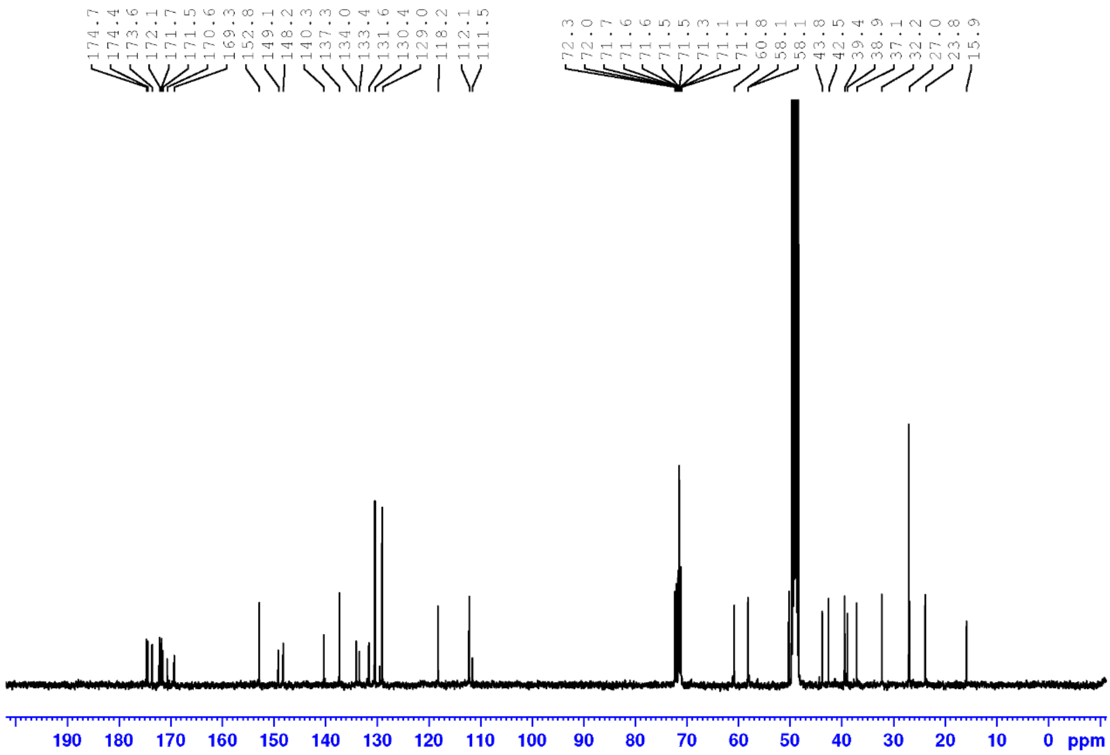

14d ^{1}H-NMR (500 MHz, MeOD)

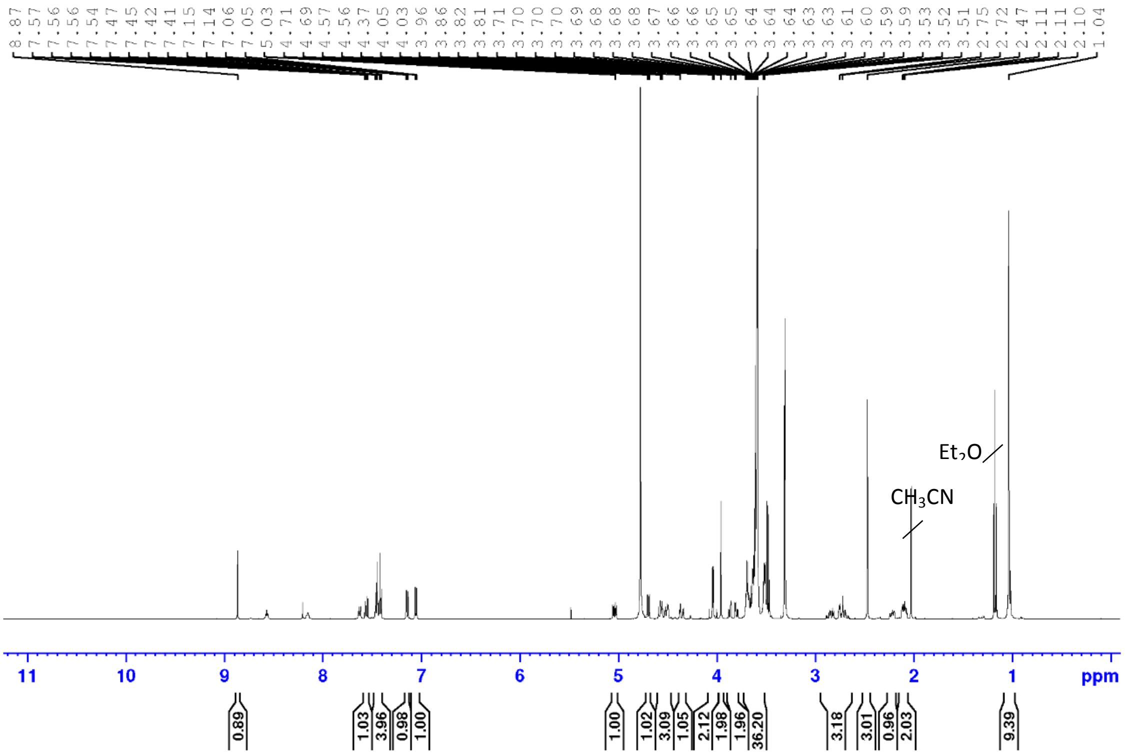

14d ^{13}C-NMR (126 MHz, MeOD)

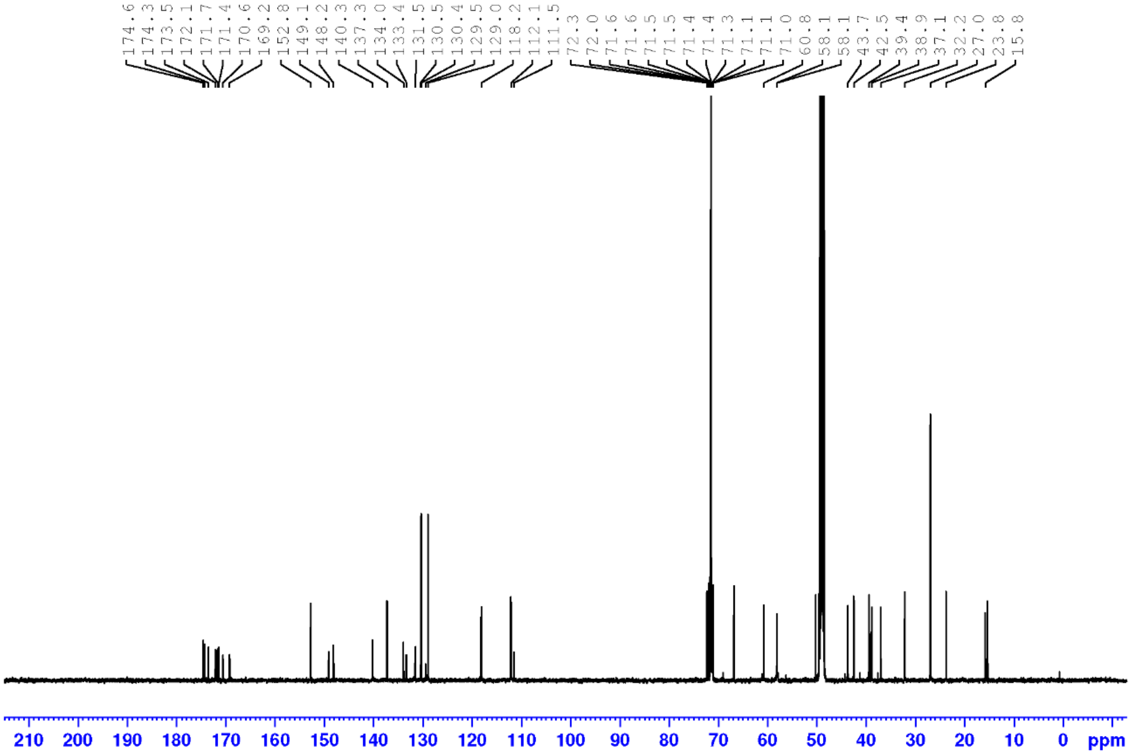

14e ^{1}H-NMR (400 MHz, MeOD)

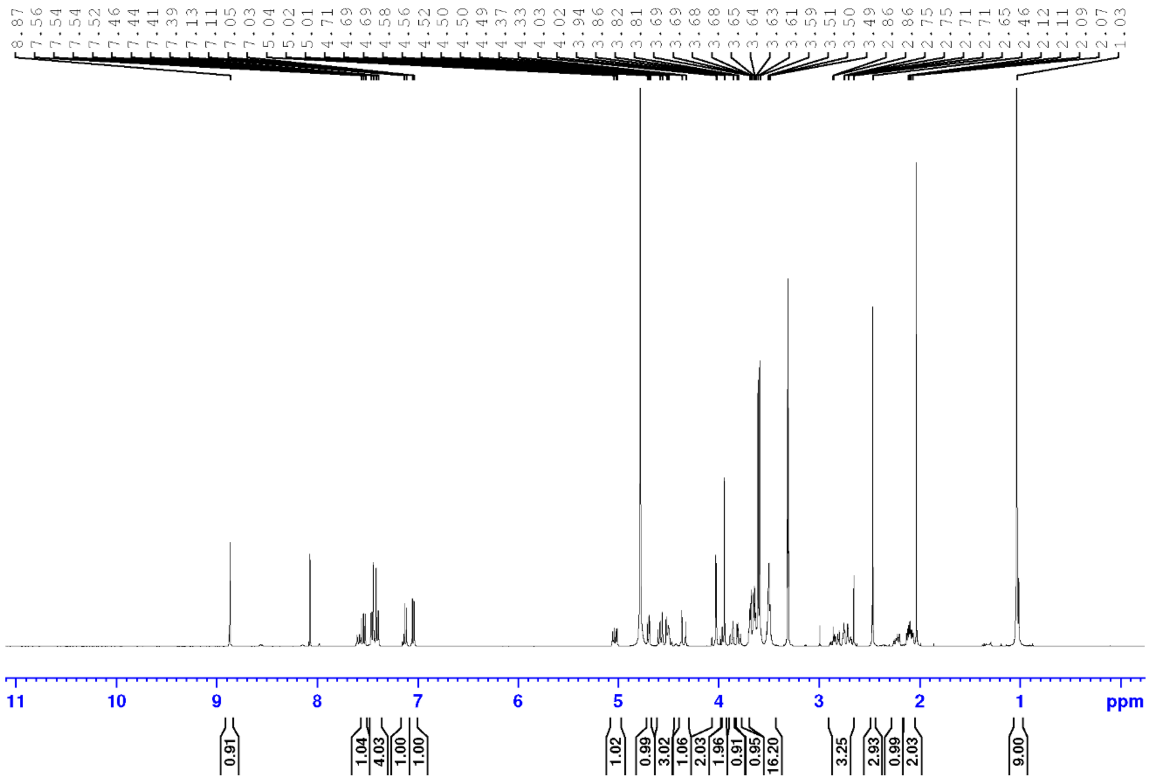

14e ^{13}C-NMR (101 MHz, MeOD)

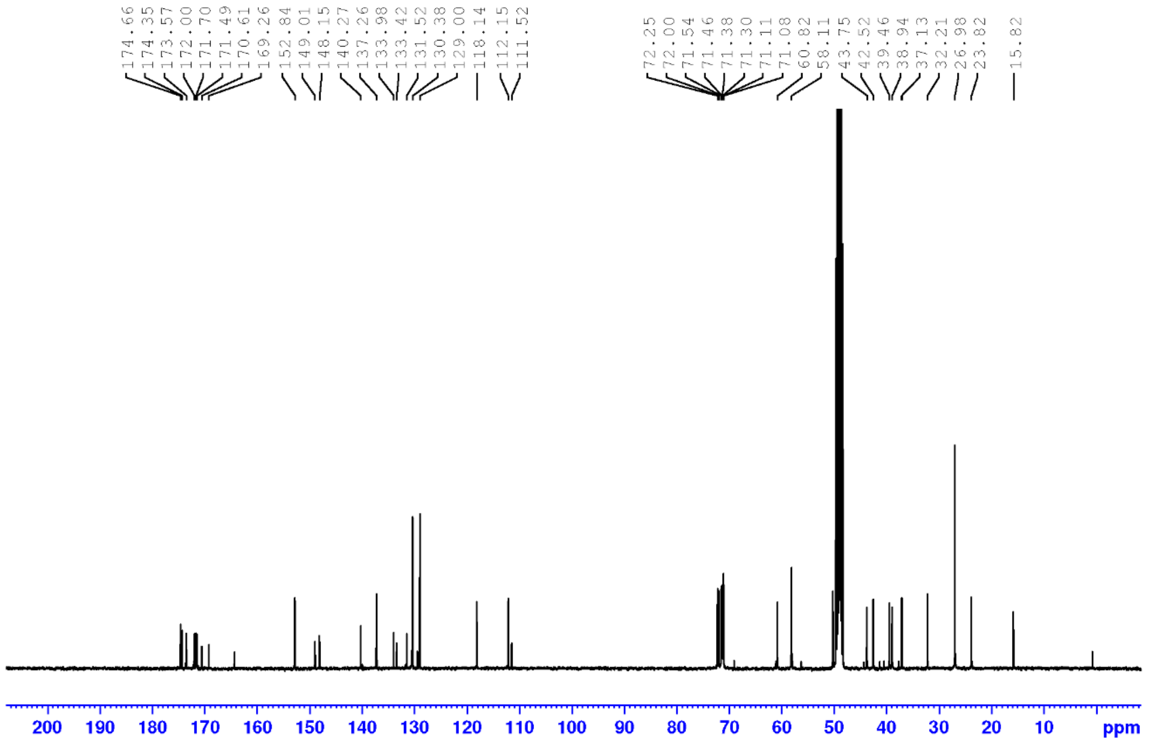

18a ^{1}H-NMR (500 MHz, $CDCl_3$)

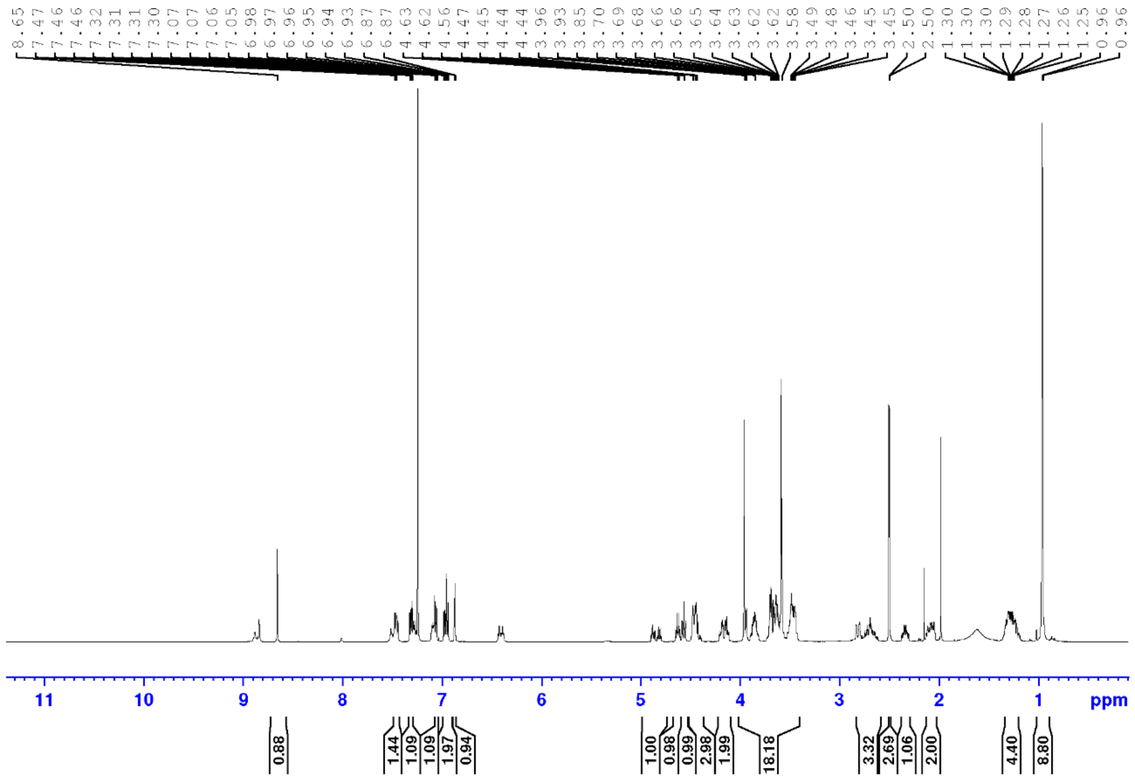

18a ^{13}C-NMR (126 MHz, $CDCl_3$)

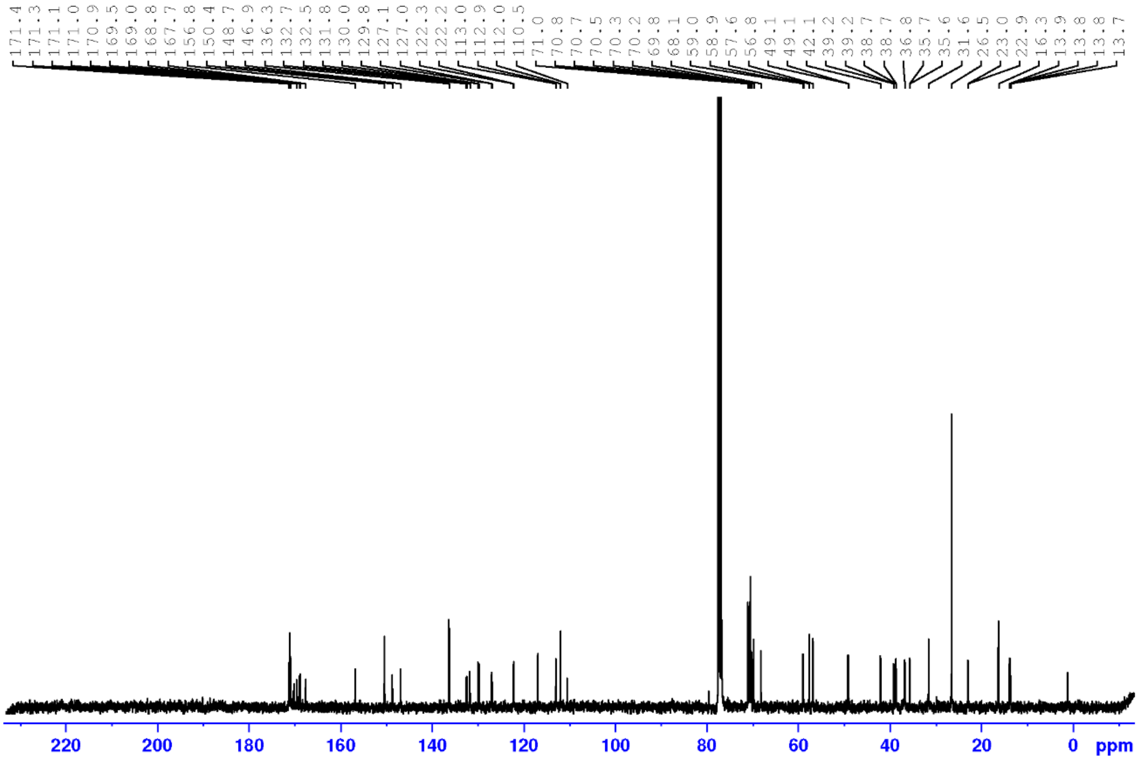

18b ^{1}H-NMR (500 MHz, $CDCl_3$)

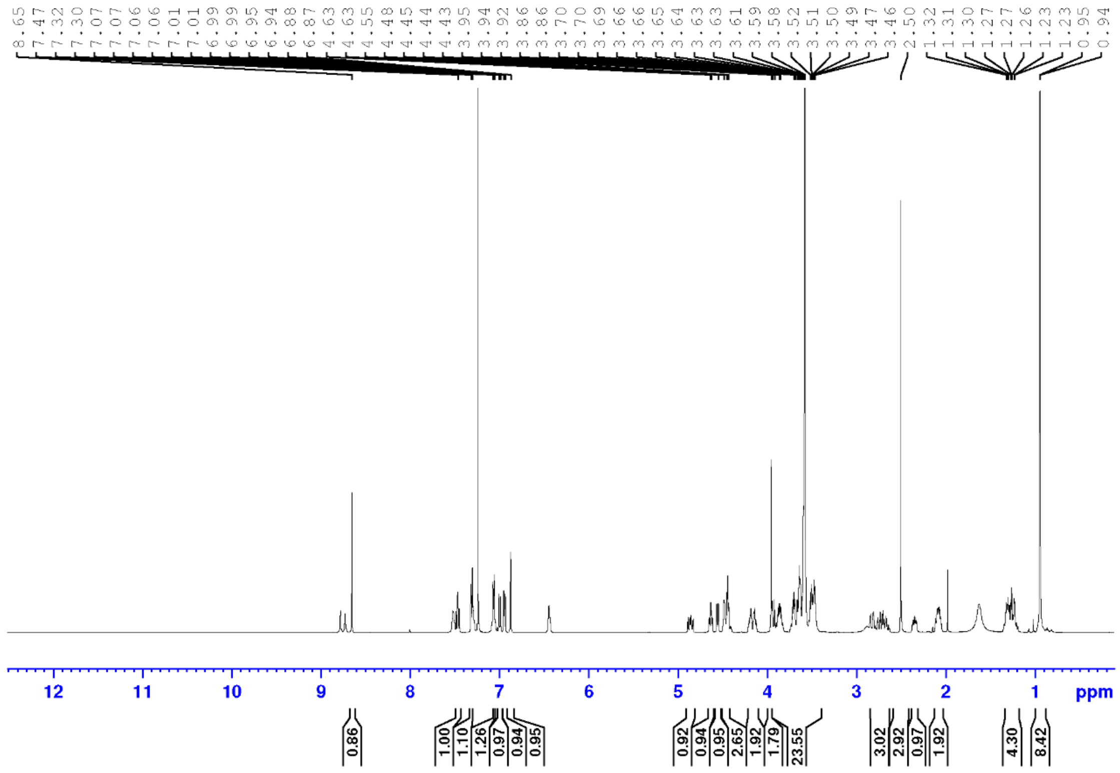

18b ^{13}C-NMR (126 MHz, $CDCl_3$)

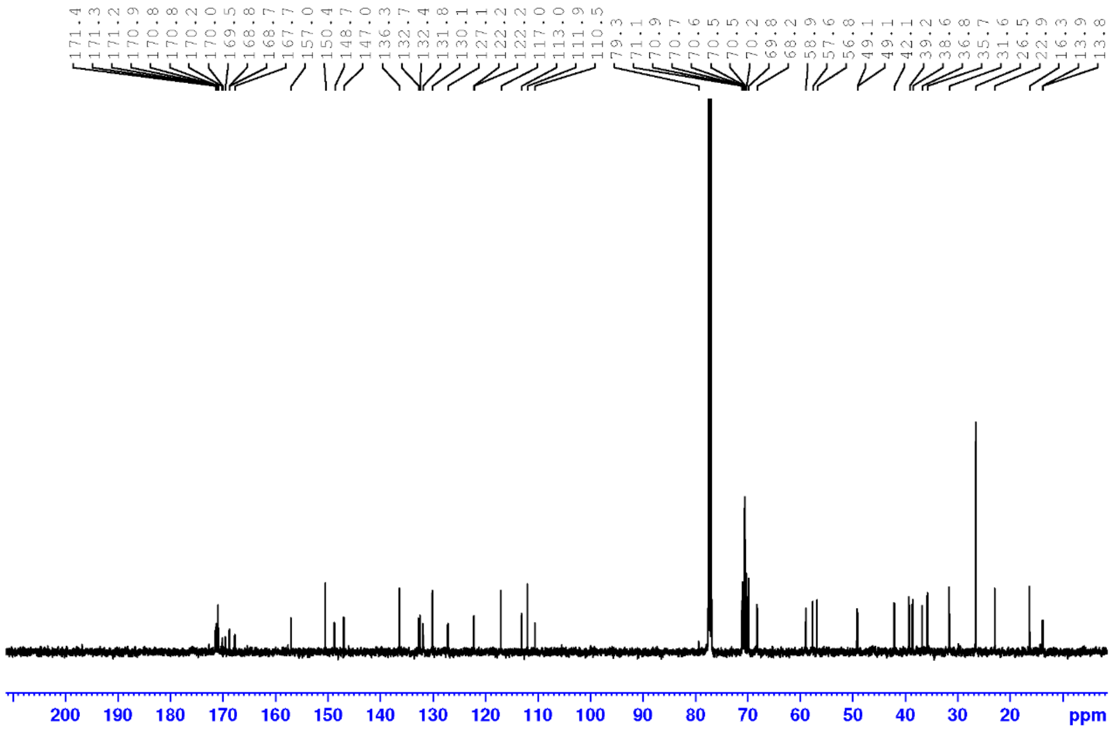

18c ^{1}H-NMR (500 MHz, $CDCl_3$)

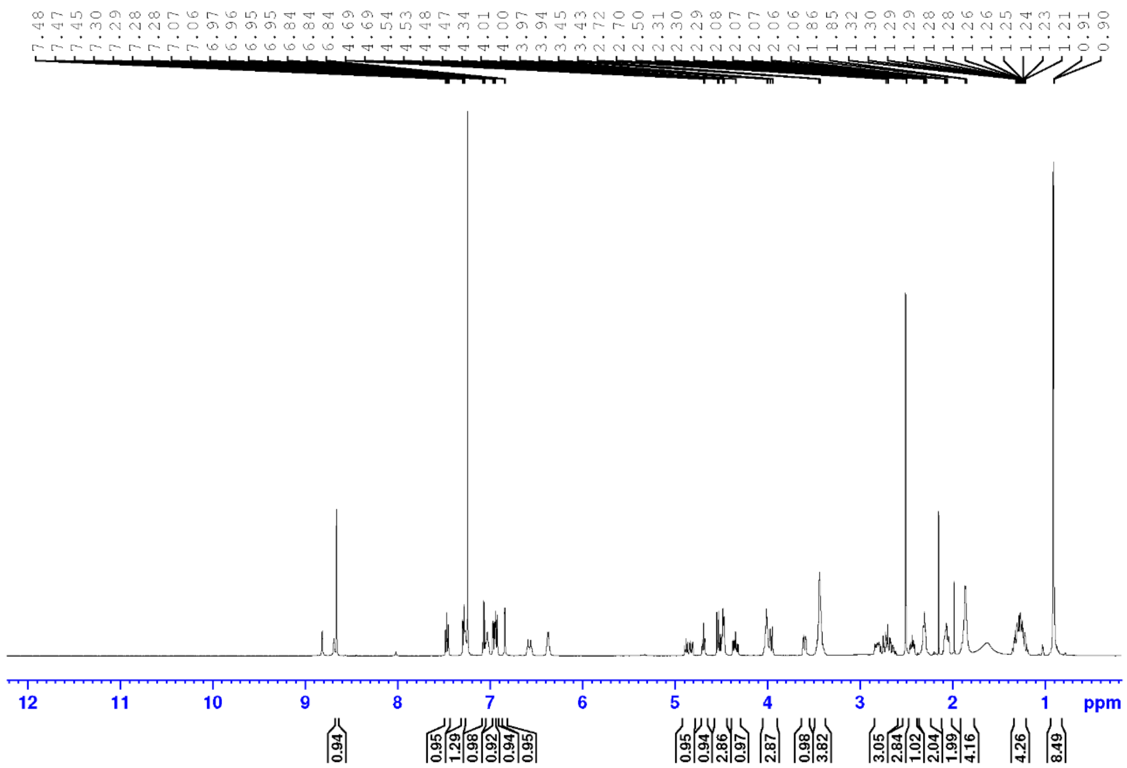

18c ^{13}C-NMR (126 MHz, $CDCl_3$)

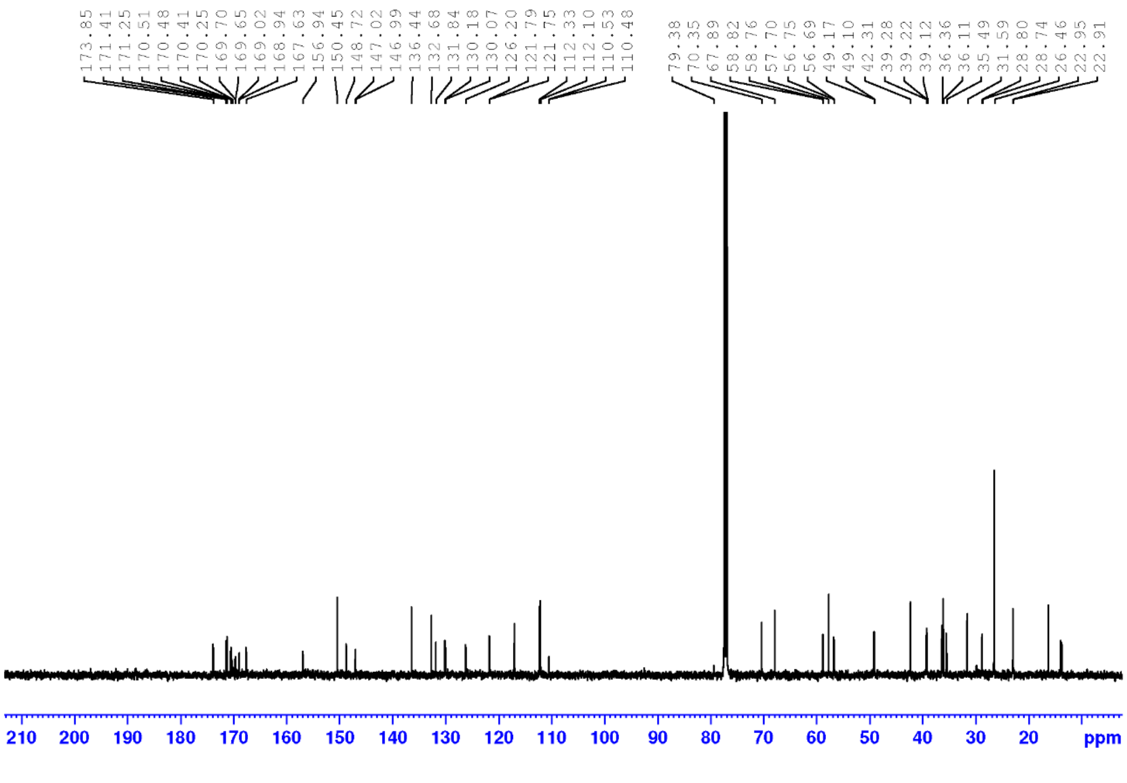

22a ^{1}H-NMR (400 MHz, MeOD)

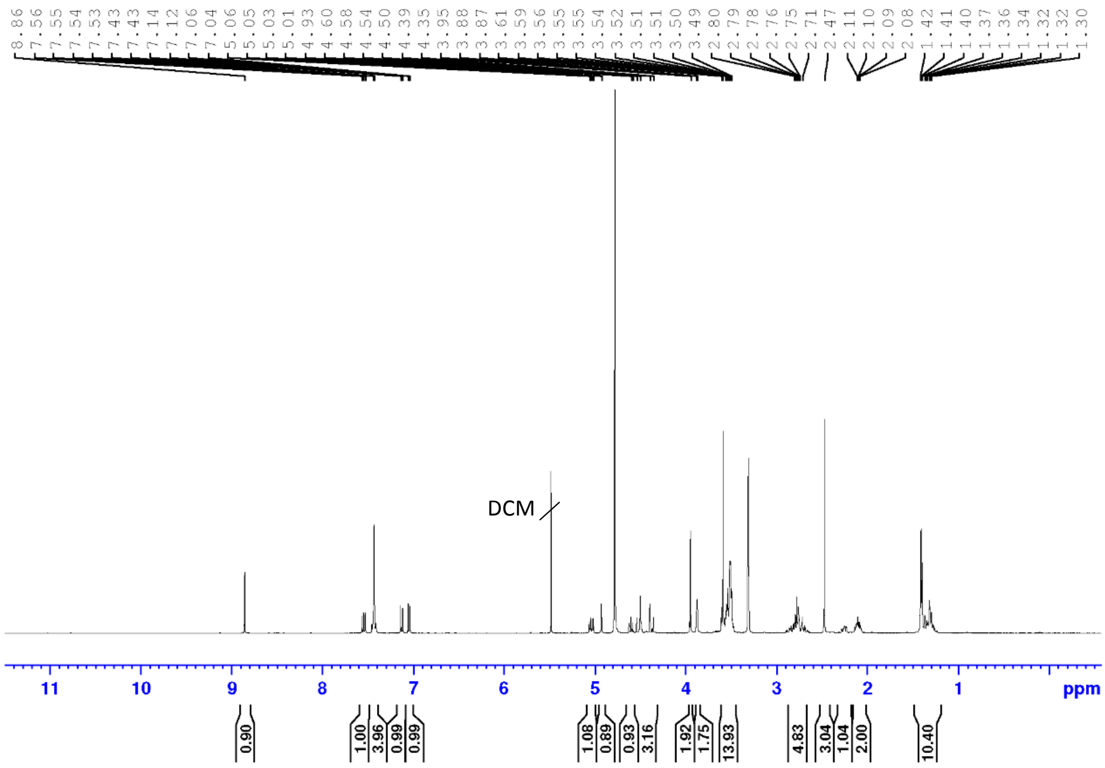

22a ^{13}C-NMR (101 MHz, MeOD)

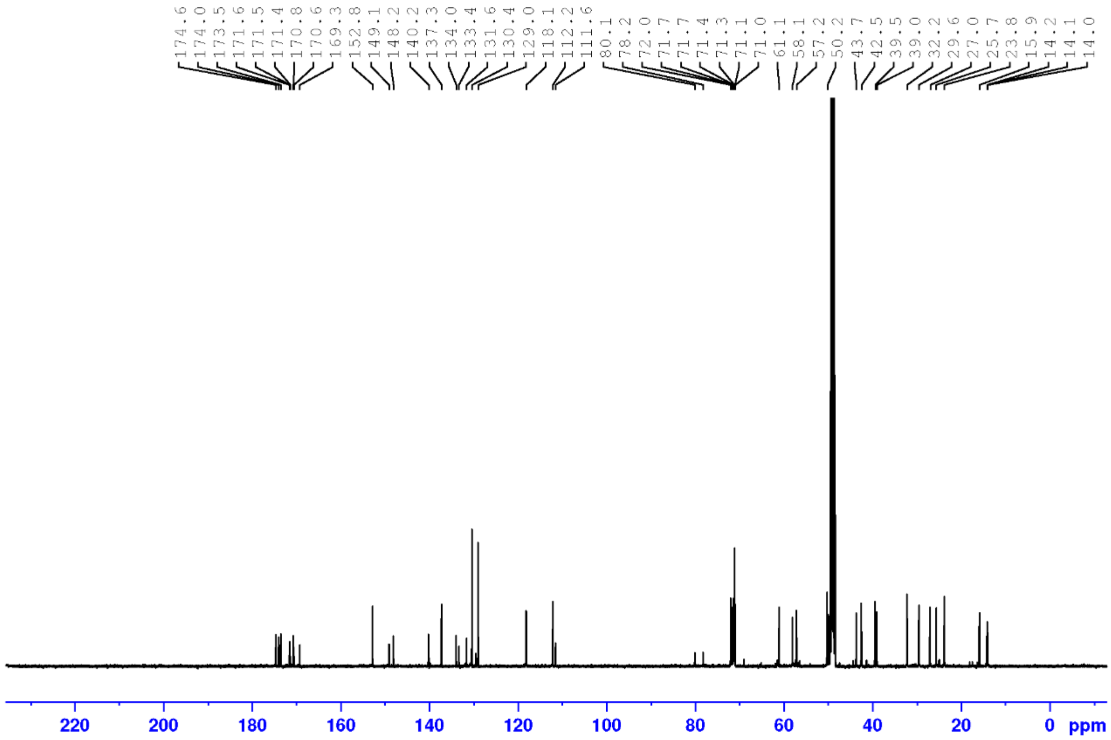

22b ^{1}H-NMR (500 MHz, MeOD)

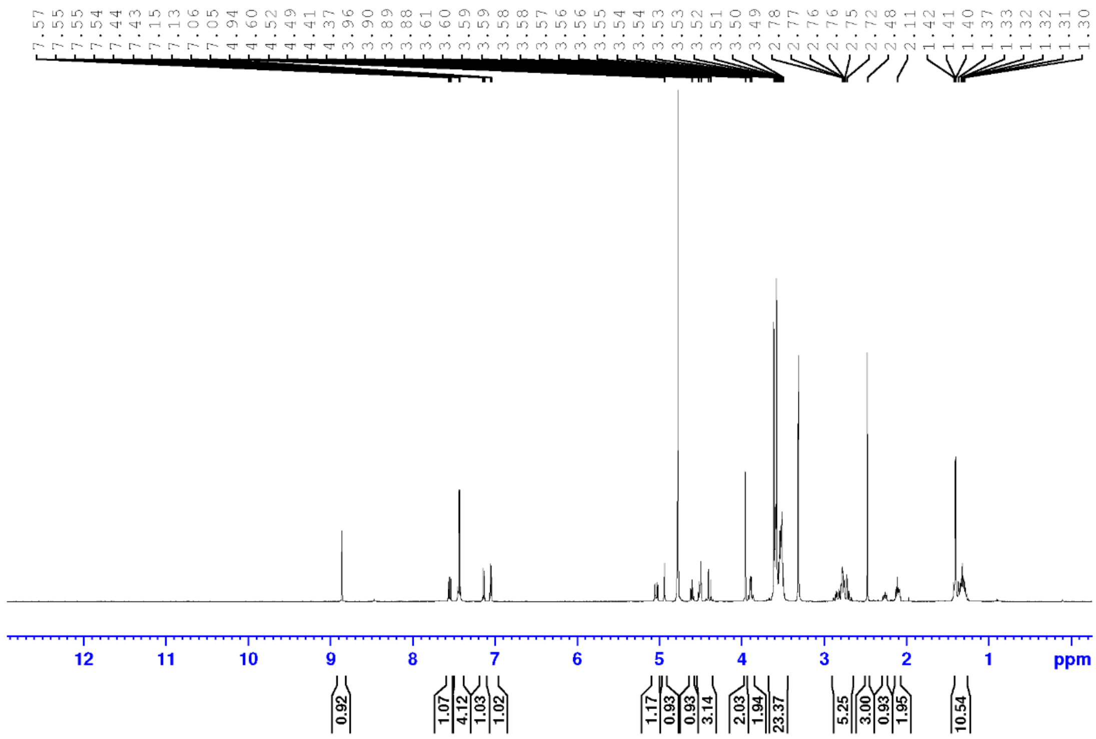

22b ^{13}C-NMR (126 MHz, MeOD)

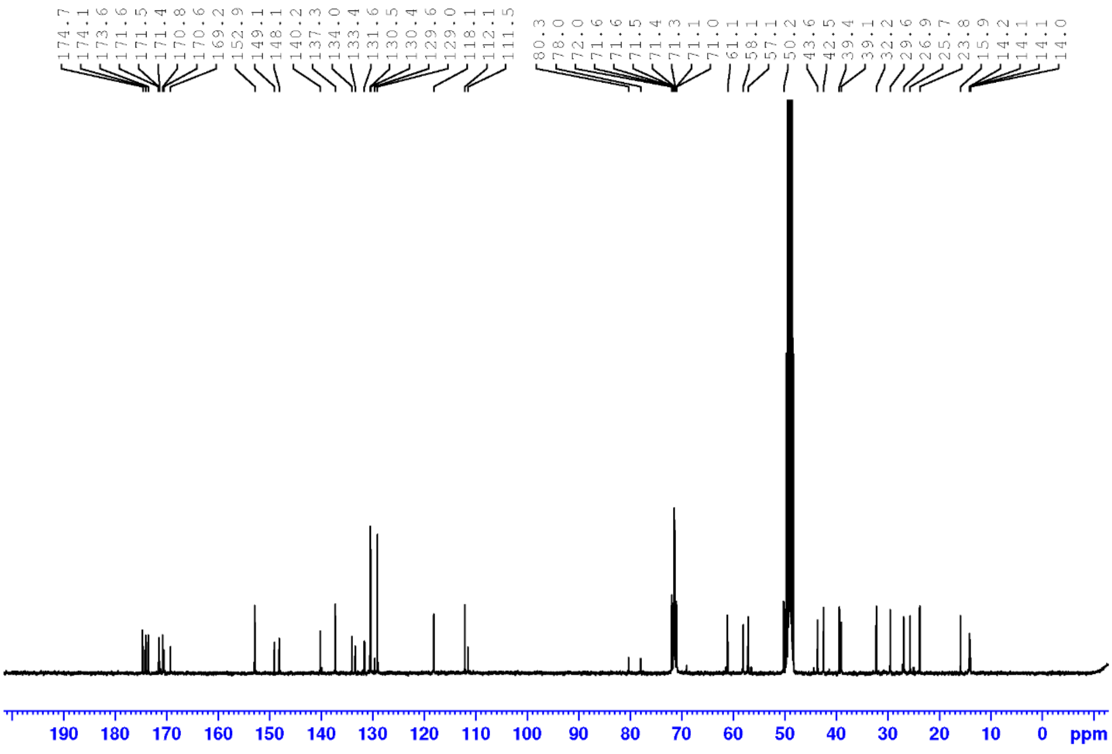

22c ^{1}H-NMR (500 MHz, MeOD)

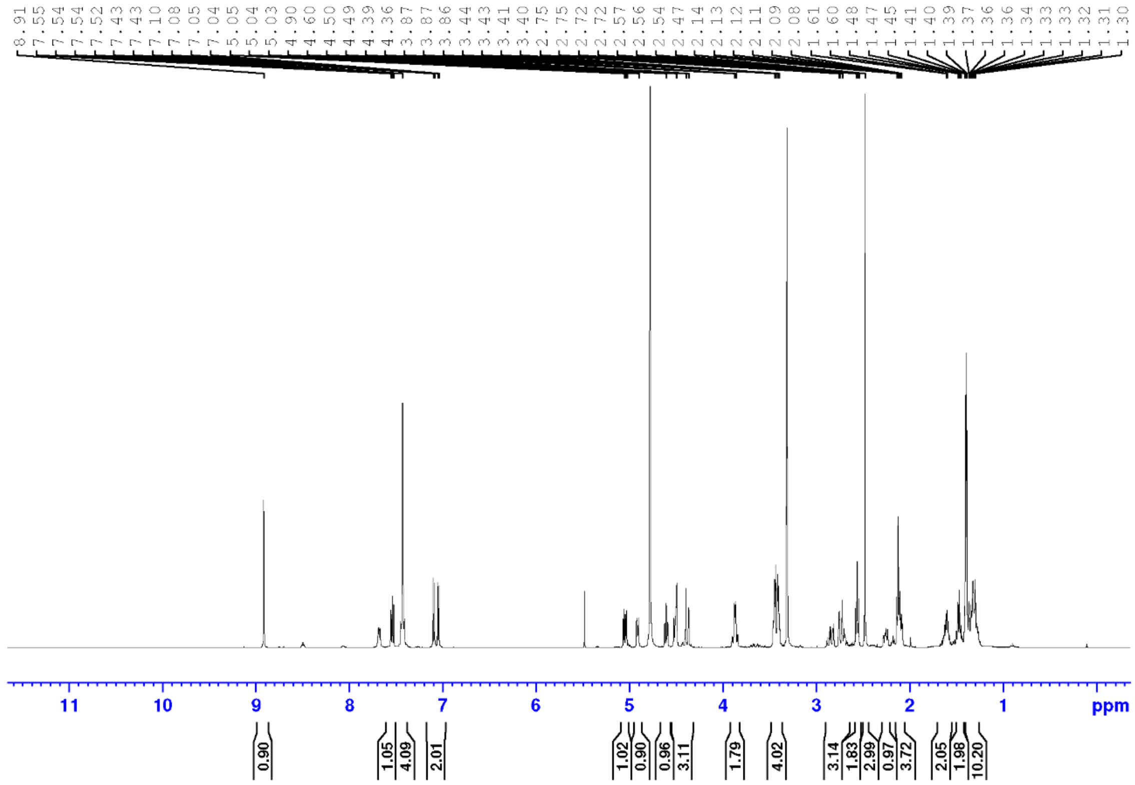

22c ^{13}C-NMR (126 MHz, MeOD)

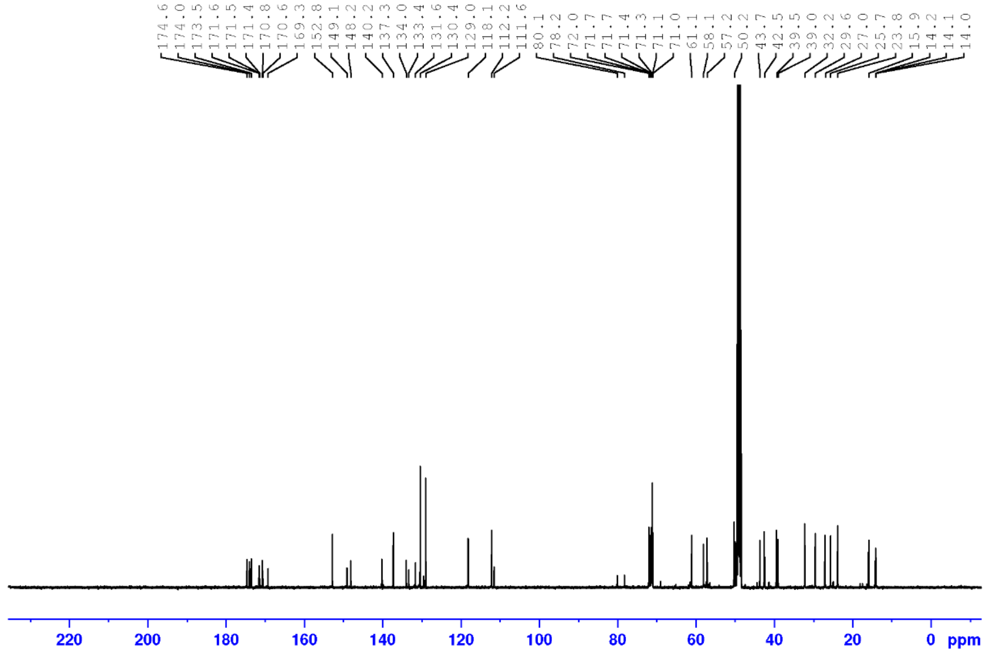

Analytical HPLC traces of final compounds

7a

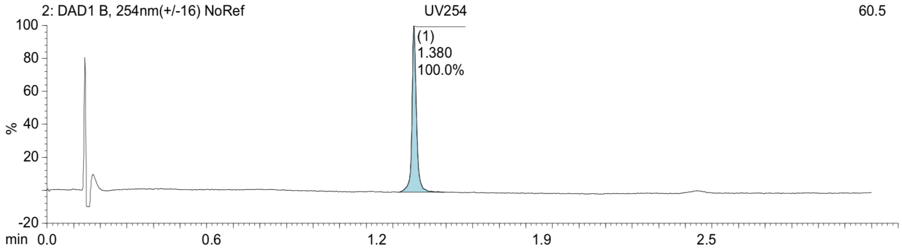

7b

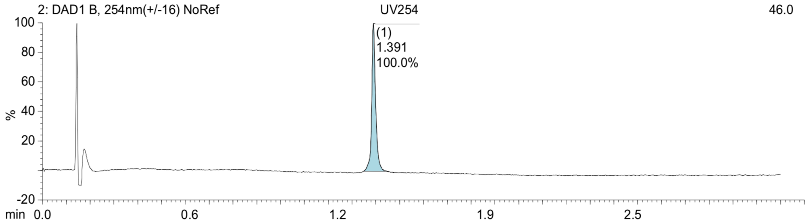

14a

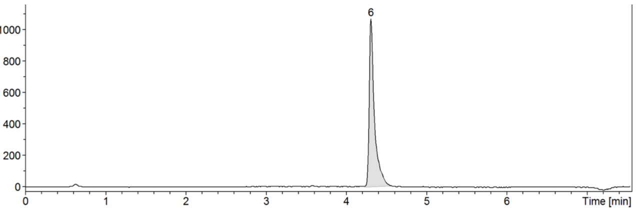

- **14b**

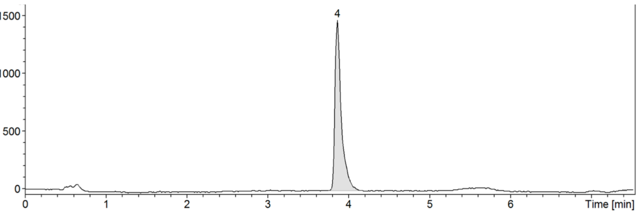

14c

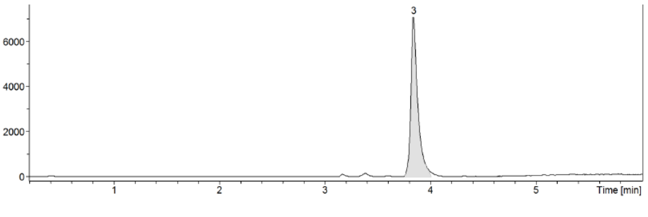

14d

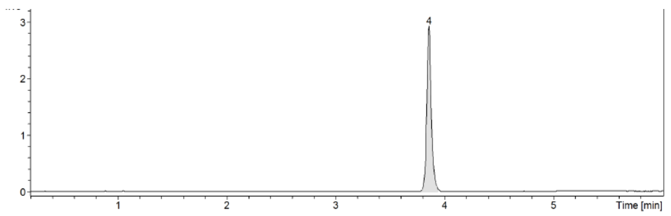

14e

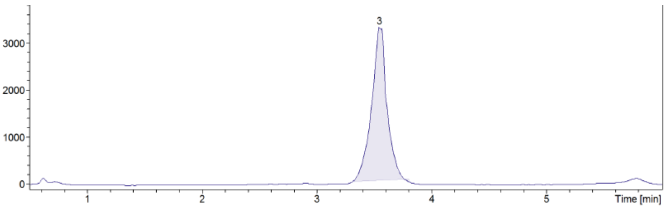

18a

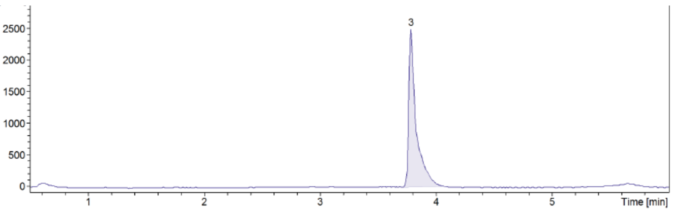

18b

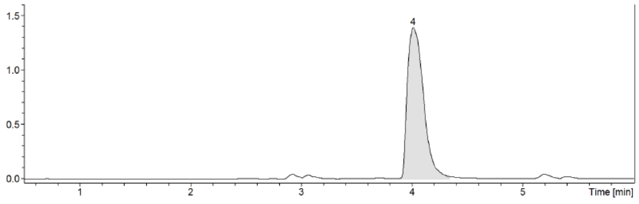

18c

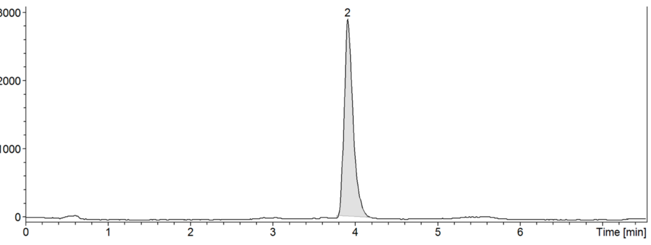

22a

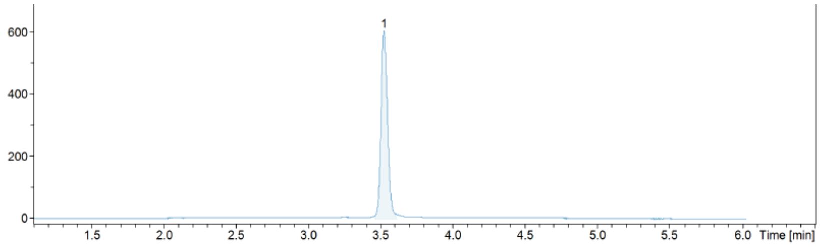
1
600
400
200
0
1.5
2.0
2.5
3.0
3.5
4.0
4.5
5.0
5.5
6.0
Time [min]

22b

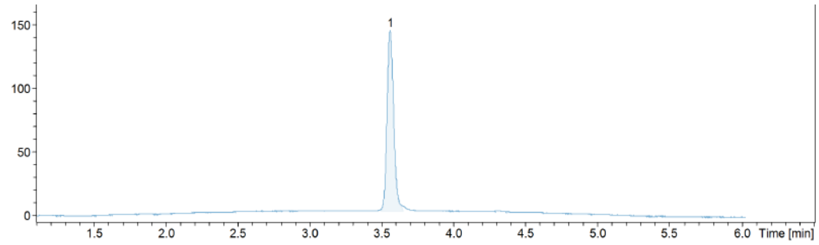
1
150
100
50
0
1.5
2.0
2.5
3.0
3.5
4.0
4.5
5.0
5.5
6.0
Time [min]

22c

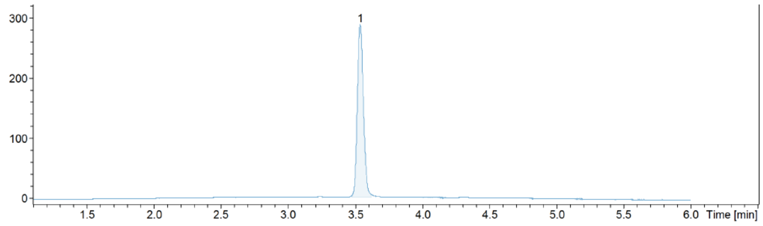
1
300
200
100
0
1.5
2.0
2.5
3.0
3.5
4.0
4.5
5.0
5.5
6.0
Time [min]

Supporting References

1. Zhang, X.; Du, X.; Huang, X.; Lv, Z. *J Am Chem Soc* **2013**, *135*, 9248–9251.
2. Paramanik, M.; Singh, R.; Mukhopadhyay, S.; Ghosh, S. K. *Journal of Fluorine Chemistry* **2015**, *178*, 47–55.
3. Wittmann, V.; Takayama, S.; Gong, K. W.; Weitz-Schmidt, G.; Wong, C.-H. *J Org Chem* **1998**, *63*, 5137–5143.
